# Effects of methylphenidate on the human vascular endothelium

**DOI:** 10.1038/s41398-026-04237-6

**Published:** 2026-07-17

**Authors:** Wenjie Cai, MaiBritt Giacobini, Cecilia Österholm, Catharina Lavebratt

**Affiliations:** 1 Karolinska Institutet, Department of Molecular Medicine and Surgery, Stockholm, Sweden; 2 Karolinska University Hospital Solna, Center for Molecular Medicine, Stockholm, Sweden; 3 PRIMA Child and Adult Psychiatry, Stockholm, Sweden

**Keywords:** ADHD, Physiology

## Abstract

Methylphenidate (MPH) is a widely used psychostimulant for treating attention-deficit/hyperactivity disorder (ADHD). Emerging epidemiological findings suggest that MPH treatment increases the risk of cardiovascular events. However, as causality could not be inferred, further investigation of the potential impact of MPH on human vascular endothelial function is warranted. We investigated the effects of MPH on human brain microvascular endothelial cells (HBEC) and human aortic endothelial cells (HAEC) across a range of concentrations, including those corresponding to therapeutic plasma levels, at 24 and 48 h. Gene expression profiling of twenty-nine endothelial function-related genes was performed. Protein expression was assessed by ELISA, Western blotting, immunofluorescence and FACS, and barrier function was evaluated using FITC-dextran permeability and electric cell-substrate impedance sensing assays. Key endothelial markers with differential levels were analysed also in plasma from ADHD children (*n* = 87) and adults (*n* = 102), and matched controls (*n* = 4 and *n* = 44, respectively). Exposure to MPH altered levels of several endothelial function-related genes. Across all concentrations tested, MPH increased the secretion of von Willebrand factor (vWF) and tissue plasminogen activator, whereas supratherapeutic concentrations 50 µM and/or 100 µM MPH decreased Claudin-5 expression and impaired endothelial barrier integrity. In clinical plasma samples, children receiving MPH showed higher vWF levels compared with medication-naïve children, but this was not observed in adult samples. Our findings suggest that acute exposure to therapeutic MPH concentration was associated with endothelial activation, and that supratherapeutic concentrations (50 µM and/or 100 µM) may further compromise barrier integrity. These experimental outcomes may propose potential effects on vascular health associated with MPH use. Further studies are needed to confirm these findings.

## Introduction

Methylphenidate (MPH) is one of the most prescribed psychostimulants for the treatment of attention-deficit/hyperactivity disorder (ADHD). As a dopamine and norepinephrine reuptake inhibitor, MPH effectively improves attention and behavioural control and is widely used as a long-term therapeutic agent in children and adults. Elevated blood pressure and heart rate have been reported in some studies as potential side effects, as have effects on sleeping and appetite [1, 2], but MPH-treatment in children for 2 years is considered safe with no adverse effect on growth, psychiatric or neurological symptoms [3]. However, recent large-scale meta analyses of epidemiological population-based studies have raised concerns about potential modest cardiovascular risks associated with long-term MPH, although inconsistencies remain [4–6]. In addition, a Swedish nationwide study of a mixed-age population with ADHD revealed a modest increase in cardiovascular disease risk, particularly hypertension and arterial disease, associated with long-term MPH use, also in those younger than 25 years [7]. In another Swedish register study of a mixed-aged cohort with ADHD, short-term MPH use was associated with a 10% increased rate of cardiovascular events six months after the initiation of MPH, in comparison with one year before treatment commencement [8]. Further, in Swedish females aged 5–30 years, MPH was more common among those with a cardiac event [9]. In a nationwide Danish case-control study, Eroglu et al. reported higher use of MPH among those with out-of-hospital cardiac arrest compared with the general adult population [10]. However, causality cannot be inferred from the aforementioned studies, and the biological basis of the associations is not yet understood.

The vascular endothelium forms a selective barrier that regulates the exchange of molecules, immune surveillance, and haemostasis. Disruption of endothelial function is a critical early event in the development of cardiovascular disease [11–13]. Our previous research found elevated plasma levels of soluble ICAM1 and VCAM1 (endothelial adhesion molecules associated with vascular inflammation) in pediatric ADHD patients undergoing psychostimulant medication treatment (mostly MPH), compared to those who were not on medication [14]. In the central nervous system, brain vascular endothelium and intercellular tight junction proteins are essential for maintaining the integrity of the blood-brain barrier (BBB), which in turn plays a crucial role in maintaining central nervous system homeostasis.

Current clinical practice for MPH treatment in ADHD typically involves dosing in the range of 0.5–1.5 mg/kg. Clinically relevant peak plasma concentrations of MPH following oral administration typically range from approximately 2 to 25 μg/L under prescribed dosing conditions [15–18]. An ADHD adolescent cohort treated with high-dose osmotic release oral system (OROS)-MPH, had mean plasma concentrations ranging from 15.5 to 49.0 μg/L [19]. Rats orally given MPH 5 mg/kg are reported to cause oxidative stress, inflammation, and BBB hyperpermeability [20]. Acute exposure to 1–300 μM MPH induced concentration-dependent neurotoxicity in mouse neural networks in vitro [21]. Orally administered 5–20 mg/kg MPH caused accumulation of astrocytes in the brain capillary wall in the rat brain [22]. Only one study has reported on the effects of MPH on human vascular endothelium: supratherapeutic concentrations (100 μM) of MPH were found to increase the permeability of brain endothelial cells through Rac-1 and NADPH oxidase (NOX)-driven oxidative stress-induction of Cav1-phosphorylation, causing caveolae-mediated transcytosis [23]. However, no study has, to our knowledge, reported on the MPH effect on a more comprehensive list of endothelial function markers.

To address these gaps, the present study investigated the effects of MPH on human vascular endothelial cells, including both brain microvascular endothelial cells (HBEC) and aortic endothelial cells (HAEC) and a larger set of vascular endothelial function genes. Using a comprehensive approach combining gene expression profiling, protein-level validation in cells and clinical samples, and functional barrier assays across a range of MPH concentrations (10 μg/L, 50 μg/L, 50 μM and/or 100 μM), we aimed to characterise the molecular and functional consequences of acute MPH exposure to the vascular endothelium.

## Materials and methods

### Cell culture and treatments

Human Aortic Endothelial Cells (HAEC, Cat.#C0065C, Thermo Fisher Scientific, Waltham, MA, USA) and Primary Human Brain Microvascular Endothelial Cells (HBEC, Cat.#ACBRI 376, Cell Systems, Kirkland, WA, USA) were cultured in EGM-2 Endothelial Cell Growth Medium-2 BulletKit (Cat.#CC-3162, Lonza, Basel, Switzerland) and maintained at 37 °C with 5% CO_2_ (Supplementary Information: Method 1). Five concentrations of MPH were included (Supplementary Table S1): negative control (0 μg/L MPH), 10 and 50 μg/L MPH, which present the average and highest peak plasma concentrations of MPH in humans in the current literature [15–19], as well as 50 μM MPH (13,488 μg/L) and 100 μM MPH (26,976 μg/L), which are commonly used in mouse and cell experiments [21, 23]. The concentrations of MPH used in the experiments did not impact cell proliferation and viability (Supplementary Method 2, Supplementary Figure S1). MPH was delivered once during the 24 or 48 h incubation, representing acute exposure conditions.

### Study participants and blood sampling

All participants were recruited between January 2016 and June 2018 at a psychiatric clinic in Stockholm, Sweden, and through local newspaper advertisements for a synbiotic intervention study in ADHD (ISRCTN57795429), approved by the Regional Ethics Review Board in Stockholm (number 2015/808-31/2 and 2017/91-31), as previously described [24–26]. Written informed consent was required for participation. Plasma samples collected before intervention from 87 children and 102 adults with ADHD, as well as 4 screened control children and 44 screened control adults without ADHD, were included in the analysis (Supplementary Method 3). The clinical characteristics of all the participants were collected through interviews and psychiatric scale scoring by psychiatric nurses (Table 1). None of the participants had autism, intellectual disability, eating disorder, diabetes, gastrointestinal diagnosis, symptoms of infection nor were on antibiotics.

**Table 1:** Clinical characteristics of the study participants.

	Children (*n* = 91)					Adults (*n* = 146)				
	Control (*n* = 4)	ADHD (*n* = 87)			*p*	Control (*n* = 44)	ADHD (*n* = 102)			*p*
		MPH (*n* = 32)	Non-MPH medicated (*n* = 23)	Medication-naive (*n* = 32)			MPH (*n* = 37)	Non-MPH medicated (*n* = 23)	Unmedicated (*n* = 42)	
Age [years]	13 ± 0.82	12.2 ± 3.0	12.9 ± 2.2	12.6 ± 2.8	0.67	37.9 ± 6.7	35.5 ± 8.6	32.1 ± 6.6	35.1 ± 7.8	0.18
Sex										
Female	1 (25)	10 (31)	6 (26)	11 (34)	0.80	26 (59)	22 (59)	8 (35)	33 (79)	0.002
Male	3 (75)	22 (69)	17 (74)	21 (66)		18 (41)	15 (41)	15 (65)	9 (21)	
BMI	−1.15 ± 0.58	0.15 ± 1.1	−0.33 ± 1.48	0.34 ± 1.41	0.50	24.76 ± 4.28	25.45 ± 4.73	25.02 ± 3.61	24.90 ± 4.47	0.92
Most recent time of ADHD medication										
Current	0 (0)	32 (100)	23 (100)	0 (0)		0 (0)	29 (78)	21 (91)	0 (0)	
In the last 3 months	0 (0)	0 (0)	0 (0)	0 (0)		0 (0)	8 (22)	2 (9)	0 (0)	
3 months to 2 years ago	0 (0)	0 (0)	0 (0)	0 (0)		0 (0)	0 (0)	0 (0)	16 (38)	
No medication the last 2 years	0 (0)	0 (0)	0 (0)	0 (0)		0 (0)	0 (0)	0 (0)	26 (62)	
Medication-naïve				32 (100)					0 (0)	
ADHD medication type										
Methylphenidate	0 (0)	32 (100)	0 (0)	0 (0)		0 (0)	37 (100)	0 (0)	0 (0)	
Lisdexamphetamine	0 (0)	0 (0)	19 (83)	0 (0)		0 (0)	3 (8)	20 (87)	0 (0)	
Dexamphetamine	0 (0)	0 (0)	1 (4)	0 (0)		0 (0)	0 (0)	8 (35)	0 (0)	
Atomoxetine	0 (0)	4 (13)	4 (17)	0 (0)		0 (0)	1 (3)	0 (0)	0 (0)	
Other medication										
Antidepressant	0 (0)	0 (0)	0 (0)	0 (0)		0 (0)	10 (27)	6 (26)	12 (29)	
Antipsychotic	0 (0)	0 (0)	0 (0)	0 (0)		0 (0)	1 (3)	2 (9)	0	
Anxiolytic	0 (0)	0 (0)	0 (0)	0 (0)		1 (2)	2 (3)	4 (17)	4 (10)	
Statin	0 (0)	0 (0)	0 (0)	0 (0)		0 (0)	0 (0)	0 (0)	1 (2)	
ADHD symptom score	0.32 ± 0.21	1.55 ± 0.58	1.32 ± 0.59	1.73 ± 0.46	0.025	1.25 ± 0.47	2.37 ± 0.53	2.26 ± 0.49	2.49 ± 0.70	0.16
Inattention symptom score	0.50 ± 0.32	1.66 ± 0.58	1.63 ± 0.71	1.94 ± 0.47	0.084	1.35 ± 0.56	2.72 ± 0.56	2.59 ± 0.52	2.72 ± 0.72	0.50
Hyperactivity symptom score	0.12 ± 0.15	1.44 ± 0.71	1.00 ± 0.65	1.52 ± 0.69	0.018	1.15 ± 0.58	2.01 ± 0.65	1.93 ± 0.76	2.25 ± 0.83	0.19

Data are presented as mean ± SD and N (%). For adults with ADHD, unmedicated means no ADHD medication currently or within the last 3 months. BMI in adults were in kg/m^2^. BMI values in children were standardized for age and sex and expressed as BMI standard deviation scores (BMI SDS) based on the growth reference from the World Health Organization (WHO). Those with ADHD had a confirmed ICD-10 F90 diagnosis. Controls had no ADHD diagnosis. None of the participants had autism, intellectual disability, eating disorder, diabetes or any gastrointestinal diagnosis. None had changed medication within the past 4 weeks, none had taken antibiotics in the past 6 weeks, and none had any symptoms of ongoing infection.

In children, current ADHD medication was as follows: *n* = 28 were on Methylphenidate (MPH) only; *n* = 4 on MPH and Atomoxetine; *n* = 18 on Lisdexamphetamine only; *n* = 1 on Dexamphetamine only; *n* = 3 on Atomoxetine only; and *n* = 1 was on Lisdexamphetamine and Atomoxetine.

In adults, current and/or in the last 3 months ADHD medication was as follows: *n* = 33 were on MPH only; *n* = 3 on MPH and Lisdexamphetamine; *n* = 1 on MPH and Atomoxetine; *n* = 16 on Lisdexamphetamine only; *n* = 3 on Dexamphetamine only; and *n* = 5 were on Lisdexamphetamine and Dexamphetamine.

Other medication included those current and/or in last 3 months.

ADHD (and inattention and hyperactivity) symptom scores were assessed using parent-reported Swanson, Nolan and Pelham scale (SNAP-IV) for children, and self-reported Adult ADHD Self-Report Scale (ASRS) for adults. Values are based on the individual’s mean of scale item scores.

Group differences were assessed using one-way ANOVA for normally distributed continuous variables, the Kruskal–Wallis test for non-normally distributed continuous variables, and Fisher’s exact test for categorical variables.

### Gene expression analysis

Gene expression analysis of biomarkers related to vascular endothelial cell function (Supplementary Table S2) was performed using the nCounter Analysis System (NanoString Technologies, Seattle, WA, USA) directly from cell lysates without RNA extraction (Supplementary Method 4). Genes were tested statistically using paired t-tests, followed by False Discovery Rate (FDR) correction. Only genes with *P*_*FDR*_ ≤ 0.05, and fold-change ≥ 1.5 or ≤ 0.67 were selected as biomarkers for subsequent protein verification (Supplementary Method 4).

### Enzyme-linked immunosorbent assay (ELISA)

Concentrations of von Willebrand factor (vWF) and tissue plasminogen activator (tPA) in cell culture supernatants and blood plasma were determined using a commercially available 96-well-format ELISA kit (Human vWF ELISA Kit, Cat.#EHVWF, Human tPA ELISA Kit, Cat.#BMS258-2, Thermo Fisher Scientific), according to the manufacturer’s protocol (Supplementary Method 5). The sample size needed to detect difference in means between two group at delta = 25%, alpha 0.01 and power 80% were estimated to be 35 per group for vWF [27], and tPA [28].

### Western blot analysis

After cell lysis and protein quantification, equal amounts of the total protein were separated by electrophoresis, transferred onto polyvinylidene difluoride membranes (Cat.#1704156, Bio-Rad) and blocked in 5% non-fat milk. The membranes were incubated with primary antibodies overnight (CLDN5, Cat.#341600, 1:500, Rabbit Polyclonal, Thermo Fisher Scientific; PECAM1, Cat.#AF806, 1:400, Sheep Polyclonal, R&D systems, Minneapolis, MN, USA; β-actin, Cat.#sc-47778, 1:3000, Mouse monoclonal, Santa Cruz, Dallas, TEX, USA), followed by incubation with respective secondary antibodies for one hour (anti-rabbit, Cat.#NA934VS, 1:10000, Cytiva, Chicago, USA; anti-sheep, Cat.#HAF016, 1:1000, R&D systems) (Supplementary Method 6). Protein signals were visualised with enhanced chemiluminescence (Cat.#1705062, Bio-Rad) and detected using the ChemiDoc imaging system (Bio-Rad). Western blot analysis was used as a semi-quantitative method to assess relative protein expression levels rather than absolute quantification. Uncropped western blot images are provided in Supplementary file 1.

### Immunocytochemistry

Cells were fixed with 4% paraformaldehyde or 95% ethanol for PECAM1 and CLDN5, respectively, and then blocked with 5% donkey serum for one hour. The cells were incubated with primary antibodies overnight (CLDN5, Cat.#341600, at 1:100 dilution, Rabbit Polyclonal, Thermo Fisher Scientific; PECAM1, Cat.#AF806, 1:100, Sheep Polyclonal, R&D systems), followed by incubation with fluorophore-conjugated secondary antibodies for one hour (Alexa Fluor 488, Cat.# A32790TR, Alexa Fluor 594, Cat.#A11016, at 1:500 dilution, Thermo Fisher Scientific). Cell nuclei were counterstained with DAPI (Cat.#62248, 1:500, Thermo Fisher Scientific) for 5 min (Supplementary Method 7). Images were acquired using LSM 900 Confocal microscope (Carl Zeiss, Oberkochen, Germany).

### Fluorescence-activated cell sorting (FACS)

After collection, the cells were incubated with primary antibodies (CLDN5, Cat.#MABT1528, 1:100, mouse monoclonal, Sigma-Aldrich, St. Louis, MO, USA; PECAM1, 1:100, Sheep Polyclonal, Cat.#AF806, R&D Systems) for 30 min. The cells were then stained with fluorophore-conjugated secondary antibodies (Alexa Fluor 488, Cat.#A11015, Alexa Fluor 647, Cat.#A31571, 1:500) for 30 min. After washing, cells were resuspended in staining buffer (PBS containing 3% FBS) and analysed with a fluorescence-activated cell sorter (FACSverse, BD Biosciences, San Jose, CA, USA) (Supplementary Method 8). Data analysis was performed using FlowJo software (version 10.10.0, BD Biosciences).

### Permeability assessment in transwell plates in vitro

In vitro paracellular permeability was assessed using Transwells (0.4 µm, Cat.#3470, Corning). Following an acute 24-h treatment with MPH, the 70 kDa FITC-dextran (Cat.#90718, Sigma-Aldrich) was added to the upper chamber. Following one-hour incubation at 37 °C, 100 μL of the medium was collected from the lower chamber (Supplementary Method 9). Fluorescence intensity was measured using a fluorescence microplate reader (SpectraMax iD3, Molecular Devices, San Jose, CA, USA, 493 and 520 nm excitation and emission, respectively). Permeability was quantified by calculating the fluorescence intensity of treatment groups relative to control groups.

### Electric cell-substrate impedance sensing (ECIS) assay

ECIS electrode arrays (Cat.#8W10E, Applied BioPhysics) were pre-stabilised with 10 mM L-cysteine in sterile water and then coated with 0.2% gelatin. Cells were seeded onto the array at a 70,000 cells/well density and inserted into the ECIS station in the incubator. Once the cells were evenly attached to the bottom of the assays, the medium was replaced with fresh medium containing different concentrations of MPH. The impedance values of the cellular monolayer were monitored in real-time using an ECIS system (ECIS Zθ, Applied BioPhysics, Troy, NY, USA) over 24 h.

### Statistical analyses

All in vitro experiments were performed with at least three independent biological replicates, as specified in each figure legend. Biological replicates were defined as independent experiments performed on different days using independently prepared cell cultures. For most experiments, measurements were conducted in technical triplicates or duplicates as specified in the figure legends. Technical replicates were averaged to obtain a single value per biological replicate.

Normality and homogeneity were assessed using Shapiro-Wilk and Levene’s tests. Group differences were analysed by one-way ANOVA or Kruskal-Wallis test, followed by Tukey’s, Dunnett’s, or Dunn’s multiple comparisons. The specific statistical methods used for each analysis are stated in the figure legends.

Plasma levels of vWF and tPA were further analysed using multivariable linear regression models. Due to right-skewed distributions, biomarker levels were log-transformed prior to analysis. The unmedicated group was used as the reference category.

Statistical analyses and graphical representations were performed using GraphPad Prism (version 9.5.1, GraphPad Software, San Diego, CA, USA) and R statistical software (version 4.4.3). Differences with a *p*-value < 0.05 were considered statistically significant.

Detailed data can be found in Supplementary file 2.

## Results

### Effects of methylphenidate on the mRNA expression in human vascular endothelial cells

**Fig. 1:**
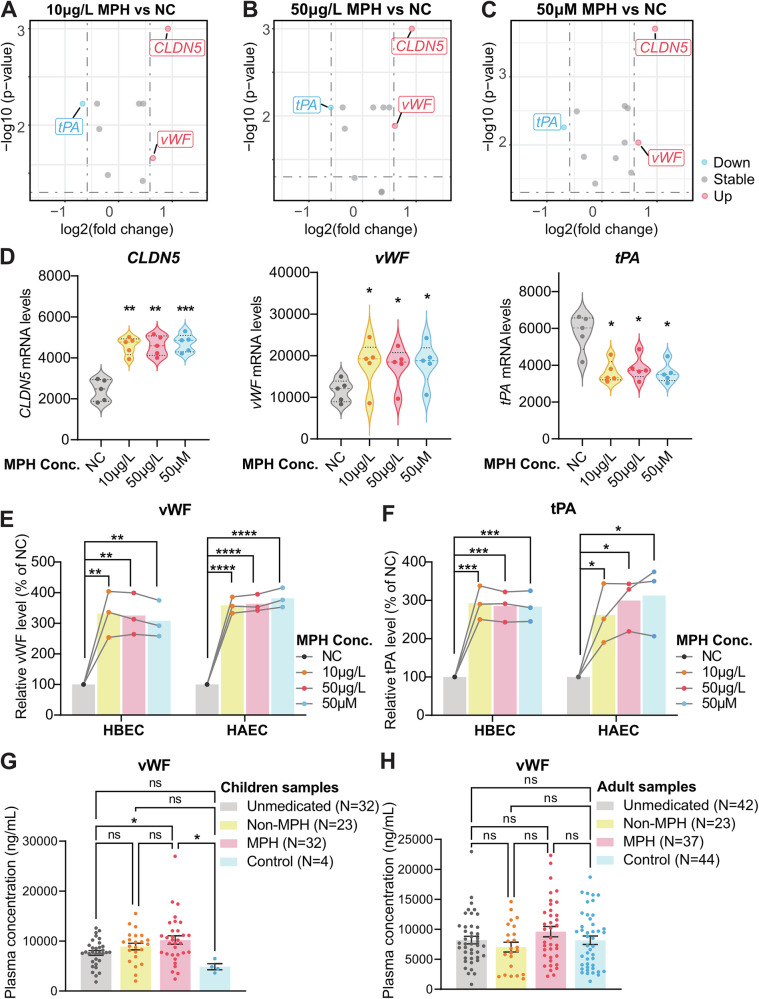
Effects of MPH on mRNA expression and protein secretion in human vascular endothelial cells and in plasma from ADHD children. **A**–**C** Volcano plots showing differentially expressed endothelial function-related genes in human brain microvascular endothelial cells (HBEC) after treatment with 10 μg/L (**A**), 50 μg/L (**B**), and 50 μM (**C**) MPH for 24 h compared to untreated controls (NC, 0 μg/L MPH). Genes for downstream analyses were selected based on FDR-adjusted *p*-value ≤ 0.05 and fold-change ≥ 1.5 or ≤ 0.67 and indicated with red and blue colours. Red dots represent upregulated genes, blue dots represent downregulated genes, and grey dots represent unselected genes. **D** Violin plots showing absolute mRNA expression readout of *CLDN5*, *vWF*, and *tPA* across the three MPH concentrations for 24 h. RNA sequencing was performed on five independent biological replicates per group (*n* = 5), with no technical replicates. Each dot represents one independent biological replicate. Statistical comparisons between each treatment group and NC were performed using paired *t*-tests, followed by FDR correction. **E,** **F** Quantification of vWF (**E**) and tPA (**F**) protein levels in cell supernatants from HBEC and human aortic endothelial cells (HAEC) after 24-h treatment with three concentrations of MPH. Protein levels are normalised to the corresponding NC within each experiment. Each dot represents one independent biological replicate, based on technical triplicates. Each grey line connects data points obtained from the same independent experiment (*n* = 3). Statistical analysis was performed using one-way ANOVA following Dunnett’s multiple comparisons test, comparing each treatment group to NC. Absolute concentration values are provided in the supplementary raw data. **G** Plasma protein levels of vWF in children with ADHD and controls. Each dot represents one independent participant’s sample measured in technical duplicate. Bars represent mean and error bars represent SD. Statistical analysis was performed using one-way ANOVA, followed by Dunn’s multiple comparisons test. Unmedicated: all children were ADHD medication-naïve, while 26 of the adults had no ADHD medication within the last 2 years, and the remaining 16 had no ADHD medication in the last 3 months. Non-MPH: the ADHD participants were currently on ADHD medication other than MPH, including Lisdexamphetamine only (*n* = 18), Dextroamphetamine only (*n* = 1), Atomoxetine only (*n* = 3) and *n* = 1 was on Lisdexamphetamine and Atomoxetine. MPH: the ADHD participants were currently on MPH (*n* = 28 took MPH only and *n* = 4 took MPH and Atomoxetine). Control: the participants without ADHD. **H** Plasma protein levels of vWF in adults with ADHD and controls. Each dot represents one independent participant’s sample. Bars represent mean and error bars represent SEM. Statistical analysis was performed using one-way ANOVA, followed by Dunn’s multiple comparisons test. Unmedicated: the ADHD participants had no ADHD medication in the last 3 months. Non-MPH: the ADHD participants were on ADHD medication other than MPH currently or in the last 3 months, including Lisdexamphetamine only (*n* = 15), Dexamphetamine only (*n* = 3) and *n* = 5 used both Lisdexamphetamine and Dexamphetamine. MPH: the ADHD participants use MPH currently or in the last 3 months (*n* = 33 use MPH only, *n* = 3 use MPH and Lisdexamphetamine and *n* = 1 uses MPH and Atomoxetine). Control: the participants without ADHD. *adjusted *P* < 0.05, ** adjusted *P* < 0.01, *** adjusted *P* < 0.001, **** adjusted *P* < 0.0001, ns: not significant.

Among the 29 endothelial function-related markers initially measured, 8 markers (*AngII, CD40L, IL1α, IL1β, MadCAM-1, NOX2, TF, and TNF-α*) were excluded from subsequent analysis since over 30% of samples had values below the LLOD (Supplementary Table S2). In HBEC, the mRNA expression levels of 7 markers were differentially expressed (compared to NC) in a similar way for all three MPH concentrations: *CLDN5*, *JAM-A*, *PAI-1*, *PECAM-1*, *TGFβ-1*, *tPA* and *vWF* (*P*_*FDR*_ ≤ 0.05; Table 2). Three of those, *CLDN5*, *vWF*, and *tPA* had fold change ≥ 1.5 or ≤ 0.67 across the three MPH concentrations (Table 2, Fig. 1A–C). The mRNA expression level of *CLDN5* was consistently upregulated across all treatments, with fold changes ranging from 1.89 to 1.95 (*P*_*FDR*_ = 0.0004–0.001, Fig. 1D, **left**). The mRNA expression level of *vWF* was also upregulated (*P*_*FDR*_ = 0.018–0.050, Fig. 1D, **middle**), and that of *tPA* showed downregulation across all treatments (*P*_*FDR*_ = 0.011–0.015, Fig. 1D, **right**).

**Table 2:** Effects of methylphenidate (MPH) treatment on mRNA expression of vascular endothelial function-related genes in human vascular endothelial cells.

Marker	HBEC						HAEC			
	10 μg/L MPH		50 μg/L MPH		50 μM MPH		50 μg/L MPH		50 μM MPH	
	Fold change	Adjusted *P*-value	Fold change	Adjusted *P*-value	Fold change	Adjusted *P*-value	Fold change	Adjusted *P*-value	Fold change	Adjusted *P*-value
*CAV1*	0.99	0.65	0.97	0.32	0.97	0.37	0.87	0.40	0.88	0.21
*CLDN5*	**1.89**	**0.001**	**1.90**	**0.002**	**1.95**	**0.0004**	**1.98**	**0.045**	**2.01**	0.18
*EDN1*	1.20	0.34	1.22	0.22	1.20	0.19	1.17	0.57	1.11	0.61
*eNOS*	1.00	0.98	1.05	0.27	1.03	0.32	0.94	0.83	0.94	0.71
*ICAM-1*	1.36	0.089	1.28	0.14	1.28	0.14	1.02	0.93	1.06	0.78
*IL6*	0.76	0.25	0.81	0.22	0.75	0.11	0.87	0.40	0.78	0.18
*IL8*	0.85	0.34	0.85	0.32	0.74	0.11	**0.63**	0.40	**0.58**	0.21
*JAM-A*	1.38	**0.013**	1.42	**0.015**	1.38	**0.006**	1.28	0.43	1.29	0.37
*MCP*	0.87	0.077	0.90	0.098	0.92	0.071	0.90	0.57	0.93	0.65
*NOX1*	**2.37**	0.45	0.96	0.83	0.98	0.81	1.16	0.57	1.32	0.21
*OCLN*	1.09	0.62	1.28	0.14	1.45	**0.049**	1.37	0.40	1.32	0.21
*PAI-1*	0.76	**0.013**	0.78	**0.015**	0.73	**0.006**	0.73	0.19	0.71	0.19
*PECAM-1*	1.30	**0.013**	1.31	**0.015**	1.33	**0.005**	1.42	0.10	1.45	0.19
*RAC1*	1.00	0.96	1.00	0.83	0.99	0.72	0.95	0.57	0.94	0.21
*SELE*	0.79	0.34	0.72	0.38	0.88	0.77	1.02	0.88	1.06	0.78
*SELP*	1.47	0.34	1.33	0.22	1.33	**0.018**	1.27	0.66	1.40	0.61
*TGFβ*	0.78	**0.026**	0.80	**0.026**	0.80	**0.029**	0.85	0.15	0.85	0.18
*tPA*	**0.63**	**0.013**	**0.66**	**0.015**	**0.62**	**0.011**	0.88	0.17	0.89	0.21
*VCAM1*	0.74	0.33	0.84	0.38	**0.60**	0.14	**0.49**	0.19	**0.54**	0.21
*vWF*	**1.56**	**0.050**	**1.52**	**0.026**	**1.58**	**0.018**	**1.60**	0.19	**1.62**	0.21
*ZO1*	1.09	0.34	1.15	**0.015**	1.19	**0.030**	1.09	0.66	1.07	0.77

The difference in expression level for each marker between the negative control (NC, 0 μg/L MPH) and MPH treatment groups was analysed by paired t-test, followed by FDR correction. FDR-adjusted *p* values ≤ 0.05 were considered significant and are highlighted in bold.

Fold change is the MPH treatment group mean divided by the NC group mean. Markers with fold change ≥ 1.5 or ≤ 0.67 are highlighted in bold.

*JAM-A* and *PECAM-1* belong to the immunoglobulin superfamily and were significantly increased following treatment with all three concentrations of MPH (*P*_*FDR*_ < 0.015, Table 2, Supplementary Fig. S2). *PAI-1* and *TGFβ-1* were significantly decreased following treatment with all three concentrations of MPH (*P*_*FDR*_ < 0.029, Table 2, Supplementary Fig. S2). *ZO1* showed a modest increase, reaching statistical significance at 50 μg/L and 50 μM (*P*_*FDR*_ < 0.030, Table 2, Supplementary Fig. S2). *OCLN* reached statistical significance only at 50 μM (adjusted *P*_*FDR*_ = 0.049, Table 2, Supplementary Fig. S2). *SELP* mRNA expression was significantly elevated only at 50 μM (*P*_*FDR*_ = 0.018, Table 2, Supplementary Fig. S2). It is co-stored with vWF in Weibel-Palade bodies following synthesis and is co-released upon endothelial stimulation. We examined the correlation between *SELP* and *vWF* mRNA expression levels in all samples from the treatment groups (10 µg/L, 50 µg/L and 50 µM MPH), and observed a strong positive correlation (*P* = 0.002, R² = 0.61, Supplementary Fig. S3).

Although statistical significance was difficult to achieve in HAECs due to the sample size (*n* = 3), size and direction of fold changes in all MPH-affected markers were consistent with those observed in HBEC (Table 2, Supplementary Fig. S4).

### Methylphenidate alters secreted levels of vWF and tPA in human vascular endothelial cells and human plasma samples

Alongside collecting cell samples for mRNA analysis, cell supernatants from the same samples were collected to assess extracellular secretion of vWF and tPA. In both cell types, treatment with all three concentrations of MPH (10 µg/L, 50 µg/L and 50 µM) significantly increased vWF secretion by more than threefold (adjusted *P* < 0.007, Fig. 1E). In contrast to the detected downregulation of tPA at the mRNA level, a more than twofold increase in tPA secretion was observed in both cell types across all three MPH concentrations (adjusted *P* < 0.049, Fig. 1F). A moderate positive correlation was observed between vWF mRNA expression and protein levels (R² = 0.353, *P* = 0.005), whereas tPA showed a weaker, non-significant association (R² = 0.086, *P* = 0.197) (Supplementary Fig. S5).

We further investigated the secretion concentration of vWF and tPA in plasma from children and adults with ADHD. In pediatric participants with ADHD, children on MPH treatment demonstrated significantly higher plasma vWF levels, compared to those medication-naive (adjusted *P* = 0.028) (Fig. 1G), while children with ADHD on non-MPH medications did not show plasma vWF levels different from those medication-naive (adjusted *P* = 0.58). Moreover, multivariable linear regression analyses demonstrated that children receiving MPH were significantly associated with higher plasma vWF levels, compared with the medication-naive group. This association was observed in both Model 1 (adjusted for ADHD symptom score; 95% CI, 0.025–0.493; *P* = 0.031) and Model 2 (further adjusted for sex and age; 95% CI, 0.037–0.503; *P* = 0.024) (Supplementary Table S3). However, there was no linear correlation for vWF to plasma levels of neither sICAM1, sVCAM1 nor CRP in ADHD children (R < 0.20, *P* > 0.18, *n* = 48, Supplementary Fig. S6). The MPH association with vWF levels was not observed in adult ADHD samples (adjusted *P* > 0.35; Fig. 1H, Supplementary Fig. S7A), including in regression analyses (Supplementary Table S4). Levels of tPA in the plasma of children were not significantly different for any of the groups (adjusted *P* > 0.96, Supplementary Fig. S7B). This remained consistent in regression analyses (Supplementary Table S3) and therefore was not assessed in plasma from adults.

### Methylphenidate at supratherapeutic concentrations reduces CLDN5 protein expression in human vascular endothelial cells

**Fig. 2:**
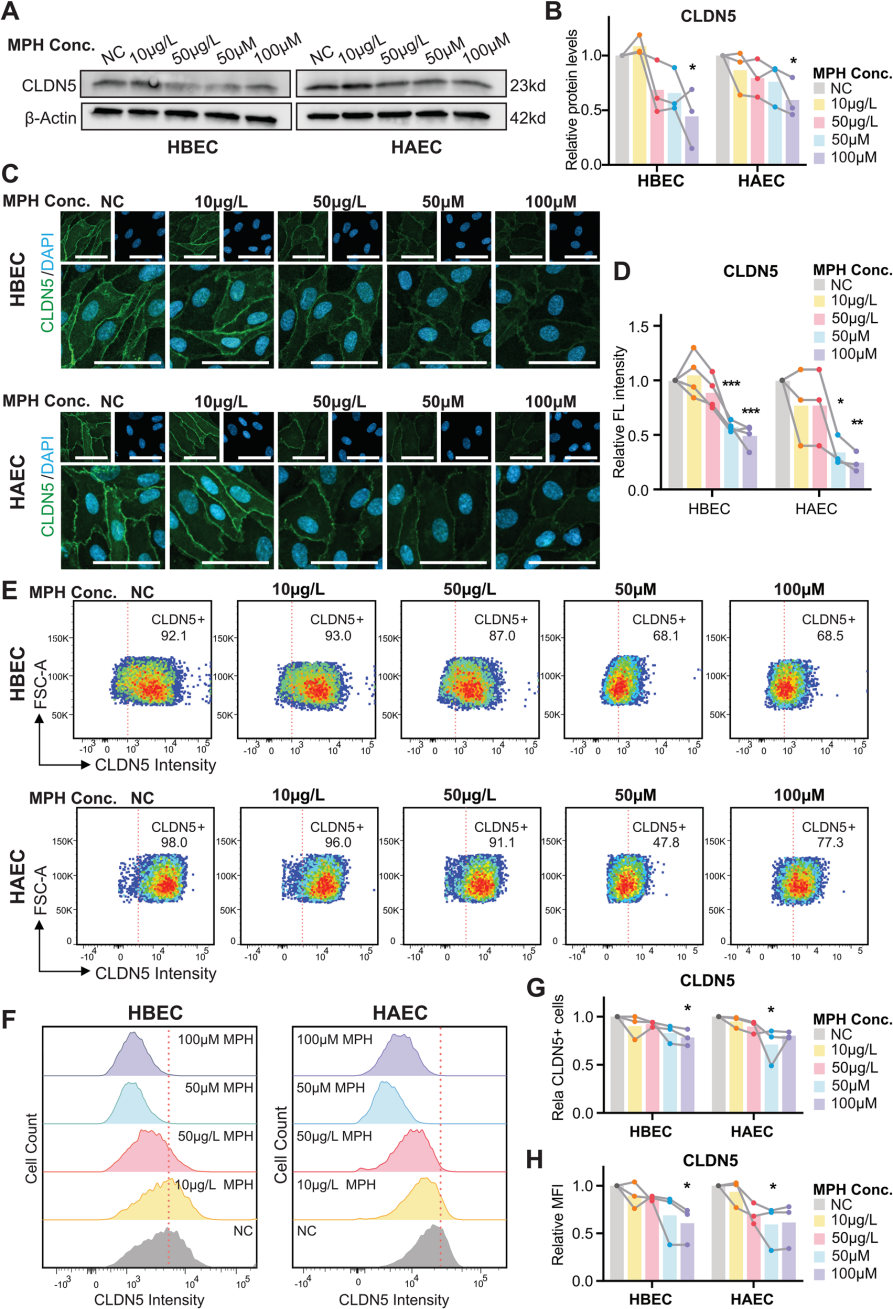
MPH at supratherapeutic concentrations reduces CLDN5 protein expression in human vascular endothelial cells. **A** Representative Western blot images of total CLDN5 protein level in human brain microvascular endothelial cells (HBEC) and human aortic endothelial cells (HAEC) following 24-h treatment with MPH. **B** Quantification of relative CLDN5 protein levels from Western blots, normalised to β-actin and negative control (NC, 0 µg/L MPH) within each experiment. Each dot represents an independent biological replicate, with no technical replicate. Each grey line connects data points obtained from the same independent experiment (*n* = 3). **C** Representative immunocytochemistry images of CLDN5 (green) and nuclei (DAPI, blue) in HBEC and HAEC following 24-h treatment with MPH. Scale bars: 50 μm. **D** Quantification of the total fluorescence intensity of CLDN5 membrane staining, normalised to the cell number and NC within each experiment. Each dot represents an independent biological replicate. Each grey line connects data points obtained from the same independent experiment (*n* = 4 for HBEC and *n* = 3 for HAEC) with technical duplicates. **E** FACS analysis of membrane CLDN5 in HBEC and HAEC following 24-h treatment with MPH. Percentages represent the proportion of CLDN5-positive cells. **F** Histogram plots showing the distribution of CLDN5 signal intensity in HBEC and HAEC. **G** Quantification of CLDN5-positive cell populations (normalised to the corresponding NC within each experiment) based on FACS data. Each dot represents an independent biological replicate with technical duplicates. Each grey line connects data points obtained from the same independent experiment (*n* = 3). **H** Relative medium fluorescence intensity (MFI) of CLDN5 in HBEC and HAEC (normalised to the corresponding NC within each experiment). Each dot represents an independent biological replicate. Each grey line connects data points obtained from the same independent experiment (*n* = 3). Statistical analysis was performed using one-way ANOVA followed by Dunnett’s post hoc test for multiple comparisons, comparing each treatment group to NC. *adjusted *P* < 0.05, **adjusted *P* < 0.01, *** adjusted *P* < 0.001. Absolute values are provided in the supplementary raw data.

We assessed the protein expression of membrane-bound CLDN5 in both cell types after treatment with four different concentrations of MPH (10 µg/L, 50 µg/L, 50 and 100 µM) for 24 h. The results of Western blot indicated that the total protein amount of CLDN5 decreased in both cell types in a concentration-dependent manner, reaching statistical significance at 100 μM (HBEC: adjusted *P* = 0.016; HAEC: adjusted *P* = 0.046; Fig. 2A, B). Consistent with the Western blot analysis, total CLDN5 protein expression of immunocytochemistry showed a concentration-dependent decrease, achieving statistical significance particularly at the two supratherapeutic concentrations, for both cell types (adjusted *P* < 0.021; Fig. 2C, D). To quantify membrane-localised CLDN5 in particular, we performed FACS using an antibody targeting the extracellular domain of CLDN5. The results demonstrated that MPH at supratherapeutic concentrations reduced membrane CLDN5 protein in both HBEC and HAEC (adjusted *P* < 0.048; Fig. 2E–H). Additionally, we observed similar results in HBEC and HAEC at 48 h after adding MPH (Supplementary Fig. S8). However, there were no significant changes in PECAM-1 protein expression after MPH acute treatment either 24 or 48 h (Supplementary Fig. S9).

### Methylphenidate at supratherapeutic concentrations impairs the human vascular endothelial cell barrier

**Fig. 3:**
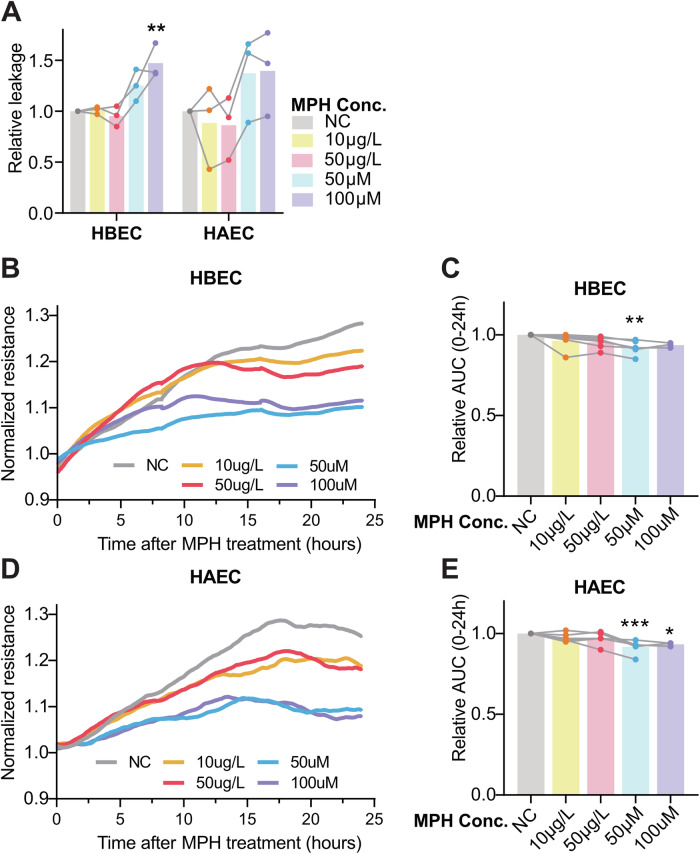
MPH at supratherapeutic concentrations impairs the human vascular endothelial cell barrier. **A** Quantification of paracellular permeability (70 kDa FITC-dextran) assessment using transwell plates in HBEC and HAEC after 24-h treatment with MPH. Leakage levels were normalised to the corresponding negative control (NC, 0 μg/L MPH) within each experiment. Each dot represents an independent biological replicate. Each grey line connects data points obtained from the same independent experiment (*n* = 3) with technical triplicates. **B,** **D** Representative resistance tracing in HBEC (**B**), and HAEC (**D**) over 24 h following the incubation with MPH. Resistance values were normalized by that at the time of adding MPH. **C,** **E** Quantification of barrier disruption by calculating the area under the curve (AUC) of normalised resistance from 0–24 h in HBEC (**C**) and HAEC (**E**). Resistance levels were normalised to the corresponding NC within each experiment. Each dot represents an independent biological replicate, with two technical replicates. Each grey line connects data points obtained from the same independent experiment (*n* = 6 for NC, 10 μg/L, 50 μg/L and 50 μM; *n* = 3 for 100 μM). Statistical analysis was performed using one-way ANOVA followed by Dunnett’s post hoc test for multiple comparisons, comparing each treatment group to NC. *adjusted *P* < 0.05, **adjusted *P* < 0.01, *** adjusted *P* < 0.001. Absolute values are provided in the supplementary raw data.

To assess the impact of MPH on endothelial barrier integrity, we conducted permeability assays in vitro. Results from the permeability assessment demonstrated that 100 μM MPH treatment for 24 h significantly increased the flux of 70 kDa FITC-dextran across HBEC monolayers (adjusted *P* = 0.0016, Fig. 3A, Supplementary Fig. S10). Additionally, we employed ECIS to evaluate the impedance of endothelial monolayers cultured on gold electrodes in real-time. While 10 and 50 μg/L MPH had minimal effects, 50 μM and/or 100 μM MPH significantly reduced the electrical resistance, indicating impaired barrier function (HBEC: adjusted *P* = 0.0047, Fig. 3B, C; HAEC: adjusted *P* < 0.015, Fig. 3D, E).

## Discussion

Although MPH has been widely used for decades in the treatment of ADHD, with the known side effects of increased blood pressure and heart rate, emerging epidemiological evidence has raised concern about potentially worse adverse effects on the cardiovascular system. However, most previous research has been largely descriptive, leaving the biological basis of this association uncertain. Our study addresses this gap by novelly integrating transcriptomic analysis, protein quantification and localisation, and barrier-functional assays on two human primary vascular endothelial cell types (from brain and aorta), with clinical plasma readouts. In this study, we demonstrated that acute exposure to MPH, even at common therapeutic plasma concentrations, alters the expression and secretion of key endothelial activation markers. However, only supratherapeutic concentrations of MPH reduce both total and membrane-bound CLDN5 protein expression and compromise vascular barrier integrity. Protein secretion findings of cells were further supported by analyses of plasma samples from ADHD patients. To our knowledge, few studies have comprehensively examined the effects of various concentrations of MPH on a larger set of endothelial function genes in human vascular endothelial cells from both the brain and the periphery, and assessed these key vascular endothelial markers in plasma from ADHD patients.

We systematically screened well-established functional markers of the vascular endothelium. Following translation from mRNA, vWF proteins are transported and stored in vascular endothelial cell-specific organelles, Weibel-Palade bodies, and are rapidly released into the extracellular space in response to endothelial cell dysfunction (e.g., inflammation, injury, or hypoxia) [29]. In our experiments, all three MPH concentrations used significantly increased vWF secretion in both HBEC and HAEC. Elevated circulating vWF is a well-established biomarker of endothelial dysfunction [30–32], and has been observed in patients with hypertension, diabetes, peripheral vascular disease, thromboembolism, and inflammatory vascular diseases [33]. Notably, in our pediatric ADHD cohort, plasma vWF levels were significantly elevated in MPH-treated children, compared with medication-naïve children. The higher plasma vWF levels in MPH-treated children might align with a direct or indirect MPH-induced endothelial cell activation, or endothelial inflammatory activation [14], although not linearly correlating with the adhesion molecules sICAM-1 and sVCAM-1. Also, higher vWF secretion might indirectly follow an unmeasured MPH-induced increase in blood pressure [34]. MPH is known to increase synaptic catecholamine levels and enhance sympathetic activity, which can result in modest increases in blood pressure and heart rate. Such hemodynamic and neurohumoral changes are well-established contributors to endothelial activation and dysfunction. In this context, the observed increases in vWF secretion may reflect endothelial responses that are consistent with sympathetic vascular stress. However, as most of our experiments were conducted in a simplified in vitro system without systemic regulatory inputs, these findings should be interpreted cautiously. In adults, however, no difference in plasma vWF levels was observed between medicated (*n* = 60), unmedicated (*n* = 42), or healthy controls (*n* = 44), possibly reflecting greater heterogeneity in unmeasured health status or medication in adults.

tPA promotes the dissolution of blood clots by inducing fibrin degradation. Endothelial cells store tPA as a pre-synthesised protein in vesicles, which are rapidly released upon stimulation [35]. In our experiments, all three concentrations of MPH treatment significantly increased the secretion of tPA in both HBEC and HAEC. Thus, these findings suggest that MPH possibly suppresses tPA transcription, while also triggering the rapid release of vesicle-stored tPA into the extracellular space. However, we detected no statistically significant difference in tPA levels in pediatric plasma between those on MPH and those not, and therefore did not measure tPA in adult plasma. The regulation of secretory pathways under pharmacological stress is complex and requires further investigation to elucidate the underlying mechanisms.

As the most abundant tight junction protein in brain microvascular endothelial cells, CLDN5 contains four transmembrane domains and forms tight junction complexes, essential for maintaining blood-brain barrier integrity [36, 37]. It is implicated in multiple central nervous system disorders, including neurodegenerative diseases, neuroinflammatory conditions, and psychiatric disorders [38, 39]. Both Western blot and immunocytochemistry demonstrated a progressive reduction in total CLDN5 protein levels with increasing MPH concentrations. The function of CLDN5 is closely related to its subcellular localization. To specifically quantify membrane-localised CLDN5, an antibody targeting the extracellular domain of CLDN5 was used in Flow cytometry. This analysis confirmed a reduction in membrane-localised CLDN5, indicating disruption of barrier structure and function. Functional permeability assays supported these findings: supratherapeutic MPH concentration (≥50 μM) disrupted endothelial barrier integrity, as evidenced by increased FITC-dextran leakage and reduced transendothelial electrical resistance. Importantly, these alterations were observed in both HBEC and HAEC, suggesting that supratherapeutic concentrations of MPH may affect vascular integrity. *CLDN5* mRNA expression was upregulated, while total and membrane proteins were decreased, which might be a compensatory increase in transcription due to the protein depletion. Children with ADHD on MPH displayed higher plasma levels of the adhesion molecules sICAM1 and sVCAM1 compared to medication-naïve children with ADHD [14]. Higher levels of these molecules are proinflammatory and promote leukocytes to attach and transmigrate through the vascular endothelial barrier. The transmigration happens through endothelial signalling that alters the localisation and destabilises tight junction proteins such as CLDN5 [40]. Ubiquitination at the L199 residue targets CLDN5 for proteasomal degradation, leading to reduced protein levels [41]. It is unknown if MPH may accelerate CLDN5 turnover by enhancing ubiquitination-dependent degradation.

No clear concentration-response was observed for tPA and vWF secretion, whereas total and membrane CLDN5 protein levels declined progressively with increasing MPH concentration, reaching significance at the highest concentration. This observation may be related to the known storage of tPA and vWF in endothelial vesicular compartments, where small stimuli can trigger rapid release [42]. Moreover, the elevated vWF levels indicate a sustained endothelial activation rather than a transient response, as they were observed not only in cells but also in the plasma of ADHD children receiving MPH treatment. In contrast, tight junction proteins exhibit more complex regulatory dynamics involving not only protein synthesis and degradation but also membrane redistribution, cytoskeletal remodelling, and reorganisation of intercellular junctions. Low-concentration MPH may exert only subtle effects on tight junction proteins, but as the concentration increases, additional signalling pathways are likely activated, ultimately leading to significant junctional disruption. Interestingly, we observed altered CLDN5 mRNA expression without clear concentration dependency, indicating that even lower concentrations of MPH still have some effects on endothelial cells. Given the limited longevity of primary human cells in vitro, our experiments were restricted to acute exposure conditions. Whether chronic low-concentration exposure may eventually lead to structural alterations in vascular tight junctions remains to be investigated.

JAM-A and PECAM-1 are adhesion molecules on the surface of endothelial cells and belong to the immunoglobulin superfamily. They facilitate leukocyte-endothelial adhesion and transmigration and are typically upregulated during endothelial activation or dysfunction [30–32, 43, 44]. *JAM-A* and *PECAM-1* mRNA levels were significantly increased across all three concentrations. However, protein-level validation of PECAM-1 revealed no significant changes in PECAM-1 protein. RAC1, NOX and CAV1 showed no MPH-induced changes at mRNA level; however their protein activity may still be modulated by MPH, consistent with a previous reported showing upregulation by 100 µM MPH [23] (Supplementary Fig. S2). The L-arginine/nitric oxide (Arg/NO) pathway serves as a central regulator of endothelial homeostasis, where NO generated by eNOS maintains vascular tone. The Arg/NO pathway was reported altered in ADHD [45, 46], and proposedly improved by MPH [45]. However, we detected no effect of MPH on eNOS mRNA levels (Supplementary Fig. S2).

To date, a single report showed that supratherapeutic concentration (100 μM) of MPH increased brain endothelial permeability [23]. However, they focused narrowly on barrier permeability-related proteins rather than broader vascular function markers, using one brain endothelial cell type and one MPH concentration (100 μM, corresponding to 26,976 μg/L) based on blood concentrations in MPH-treatment mouse models [21, 47], being substantially higher than those in humans [15–19]. The metabolic differences between rodents and humans limit accurate replication of human pharmacokinetics.

## Limitations

It is challenging to precisely simulate intravascular MPH concentrations. MPH exposure in this study was conducted under static in vitro conditions at peak plasma concentrations without metabolic clearance, which may result in greater cumulative exposure than the pharmacokinetic profiles occurring in medicated patients. In addition, the experimental design reflects acute exposure rather than the chronic exposure observed clinically. However, the average daily dose of MPH across different age groups is relatively consistent, approximately 1.0 mg/kg body weight [18]. Moreover, there are no significant differences in MPH pharmacokinetics between children and adults [48, 49]. Based on the current literature, we selected both an average peak plasma concentration and a reported upper-limit peak plasma concentration. Plasma concentrations around 50 μg/L are not typically achieved under standard FDA-approved dosing regimens. Therefore, findings observed at 50 μg/L primarily reflect endothelial responses under conditions of higher-than-normal clinical exposure. Some effects, particularly on CLDN5 expression and barrier function, were observed mainly at supratherapeutic concentrations, which may limit direct clinical extrapolation. While our cellular models offer some mechanistic insights into how MPH affects endothelial cell function, they cannot fully recapitulate the complexity of the vascular system in vivo, particularly the highly specialised blood-brain barrier architecture. HBECs were obtained from a healthy male pediatric donor, while the HAECs were derived from a 56-year-old male donor. However, it is unknown if they had ADHD or the adult had cardiovascular disease, which may represent a potential limitation. In addition, both HBECs and HAECs were derived from a single donor for each cell type. Therefore, although multiple independent experiments were performed, donor-level biological variability could not be assessed. ELISA data of the supernatants were not normalised to the final cell number. Although equal numbers of cells were initially seeded and no major differences in cell viability were observed, the lack of normalisation may have introduced minor variability between conditions. Plasma samples were available from only four children without ADHD, which limited the characterisation of plasma marker levels in healthy control children. Some of the screened healthy controls were either family or friends of a participant with ADHD. Given the high heritability of ADHD, shared genetic or familial factors may represent a potential source of confounding. Our primary analyses focused on comparisons within the ADHD population according to treatment status (MPH vs. unmedicated), rather than comparisons with healthy controls. Therefore, the potential impact of familial relatedness on the main findings is likely limited. The lack of adult tPA measurements represents a limitation and warrants further investigation.

Patient’s blood pressure data and cardiovascular disease history were unavailable. Information on the dosage and duration of MPH treatment was not available in the present cohort, which limits the ability to assess cumulative exposure effects. Clinical relevance needs to be interpreted with caution. Larger longitudinal cohorts are needed to assess the long-term vascular impact of MPH treatment to validate our findings.

## Conclusions

In our study MPH exposure was associated with endothelial activation and supratherapeutic concentrations further compromised barrier integrity in both human brain and peripheral vascular endothelial cells. Our findings contribute to the epidemiological reports on potential cardiovascular risks associated with MPH exposure, particularly in individuals undergoing long-term MPH treatment. These results underscore the need for further in vivo studies and longitudinal clinical investigations to assess the long-term vascular effects of MPH treatment and their possible clinical relevance.

## Supplementary information

Supplementary figures, tables and methods

Supplementary file 1_WB raw pictures

Supplementary file 2_raw data

## Data Availability

All raw data generated or analysed during this study, including gene expression, ELISA, flow cytometry, immunocytochemistry, Western blot raw pictures and quantification data, and function assays, are provided in the accompanying supplementary files.
