# Supplementary material for: Effects of methylphenidate on the human vascular endothelium
Source: Transl Psychiatry. 2026 Jul 17;16:369. doi: 10.1038/s41398-026-04237-6 (PMC13379382; doi:10.1038/s41398-026-04237-6)
Supplement: Supplementary file 1 — Supplementary figures, tables and methods [file 41398_2026_4237_MOESM1_ESM.pdf]

Supplementary Information

Title: Effects of methylphenidate on the human vascular endothelium

Authors: Wenjie Cai[1,2], MaiBritt Giacobini[1,3] Cecilia Österholm[1], Catharina Lavebratt[1,2,*]

Affiliations

[1] Karolinska Institutet, Department of Molecular Medicine and Surgery, Stockholm, Sweden;

[2] Karolinska University Hospital Solna, Center for Molecular Medicine, Stockholm, Sweden.

[3]RIMA Child and Adult Psychiatry, Stockholm, Sweden.

Corresponding author: Catharina Lavebratt, Karolinska University Hospital, House L8:00, 171 76 Stockholm, Sweden, catharina.lavebratt@ki.se +46739310710

Supplementary Figures

Supplementary Figure S1. Effects of MPH on human vascular endothelial cell proliferation and viability.

Supplementary Figure S2. Effects of MPH on mRNA expression of vascular endothelial function-related genes in human brain microvascular endothelial cells (HBEC).

Supplementary Figure S3. Correlation between *vWF* and *SELP* mRNA expression levels in all samples from the treatment group in human brain microvascular endothelial cells (HBEC).

Supplementary Figure S4. Effects of MPH on mRNA expression of vascular endothelial function-related genes in human aortic endothelial cells (HAEC).

Supplementary Figure S5. Correlation between mRNA readouts and secreted protein levels, including all samples of human brain microvascular endothelial cells (HBEC) and human aortic endothelial cells (HAEC).

Supplementary Figure S6. Correlation between plasma levels of vWF and sICAM1, sVCAM1 and CRP in ADHD children (n=48).

Supplementary Figure S7. Plasma protein levels of tPA and vWF in participants.

Supplementary Figure S8. MPH at supratherapeutic concentrations reduces CLDN5 protein expression in human vascular endothelial cells after 48 hours.

Supplementary Figure S9. MPH treatment does not significantly alter PECAM1 protein expression in human vascular endothelial cells.

Supplementary Figure S10. MPH at supratherapeutic concentrations impairs the human vascular endothelial cell barrier (pilot study of Figure 3A, with only NC, 10μg/L, 50μg/L and 100μM).

Supplementary Figure S11. Effect of a single freeze-thaw cycle on plasma von Willebrand factor (vWF) and tissue plasminogen activator (tPA) concentrations.

Supplementary Tables

Supplementary Table S1. MPH concentrations and references behind their selection in this study.

Supplementary Table S2. List of all genes in the nCounter panel studied for expression.

Supplementary Table S3. Associations between MPH exposure and plasma vWF and tPA in multivariable linear regression models in children.

Supplementary Table S4. Associations between MPH exposure and plasma vWF and tPA in multivariable linear regression models in adults.

Supplementary Methods

1. Cell culture and treatments.
2. Cell proliferation and viability assays.
3. Study participants and blood sampling.
4. Gene expression analysis.
5. Enzyme-linked immunosorbent assay (ELISA).
6. Western blot analysis.
7. Immunocytochemistry.
8. Fluorescence-activated cell sorting (FACS).
9. Permeability assessment in transwell plates *in vitro*.
10. Multiplex immunoassay for plasma levels of sICAM-1, sVCAM-1 and CRP.

Supplementary Figures

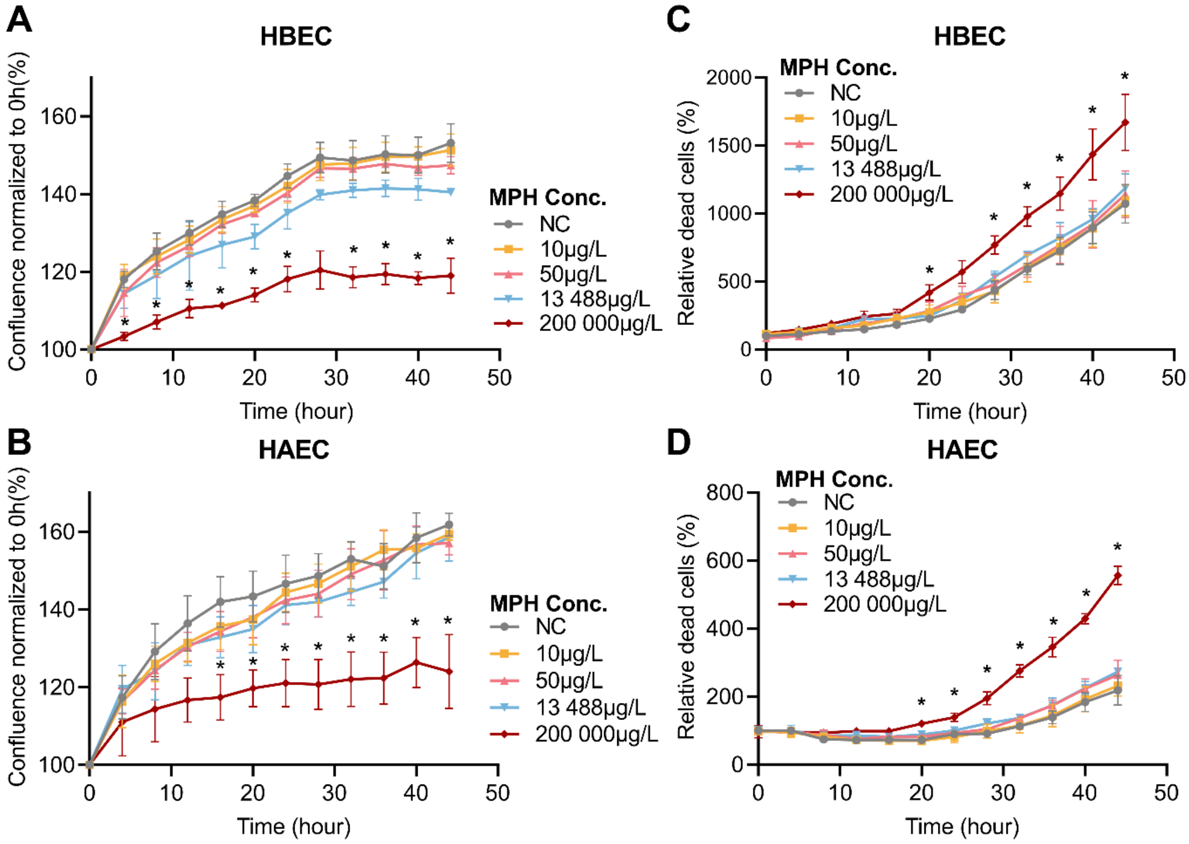

Supplementary Figure S1. Effects of MPH on human vascular endothelial cell proliferation and viability. (**A, B**) Real-time monitoring of cell confluence in human brain microvascular endothelial cells (HBEC) (A) and human aortic endothelial cells (HAEC) (B) over 48 hours following treatment with different concentrations of MPH. Confluence was normalised to the value at 0 hours. (**C, D**) Quantification of dead cell counts in HBEC (C) and HAEC (D) under the same treatment conditions, normalised to the value at 0 hours and NC.

Data represent mean ± SD from three independent experiments (n=3). Absolute values are provided in the supplementary raw data. Statistical analysis was performed using two-way ANOVA followed by Dunnett's post hoc test for multiple comparisons, compared to NC at each time point. *adjusted $P<0.05$.

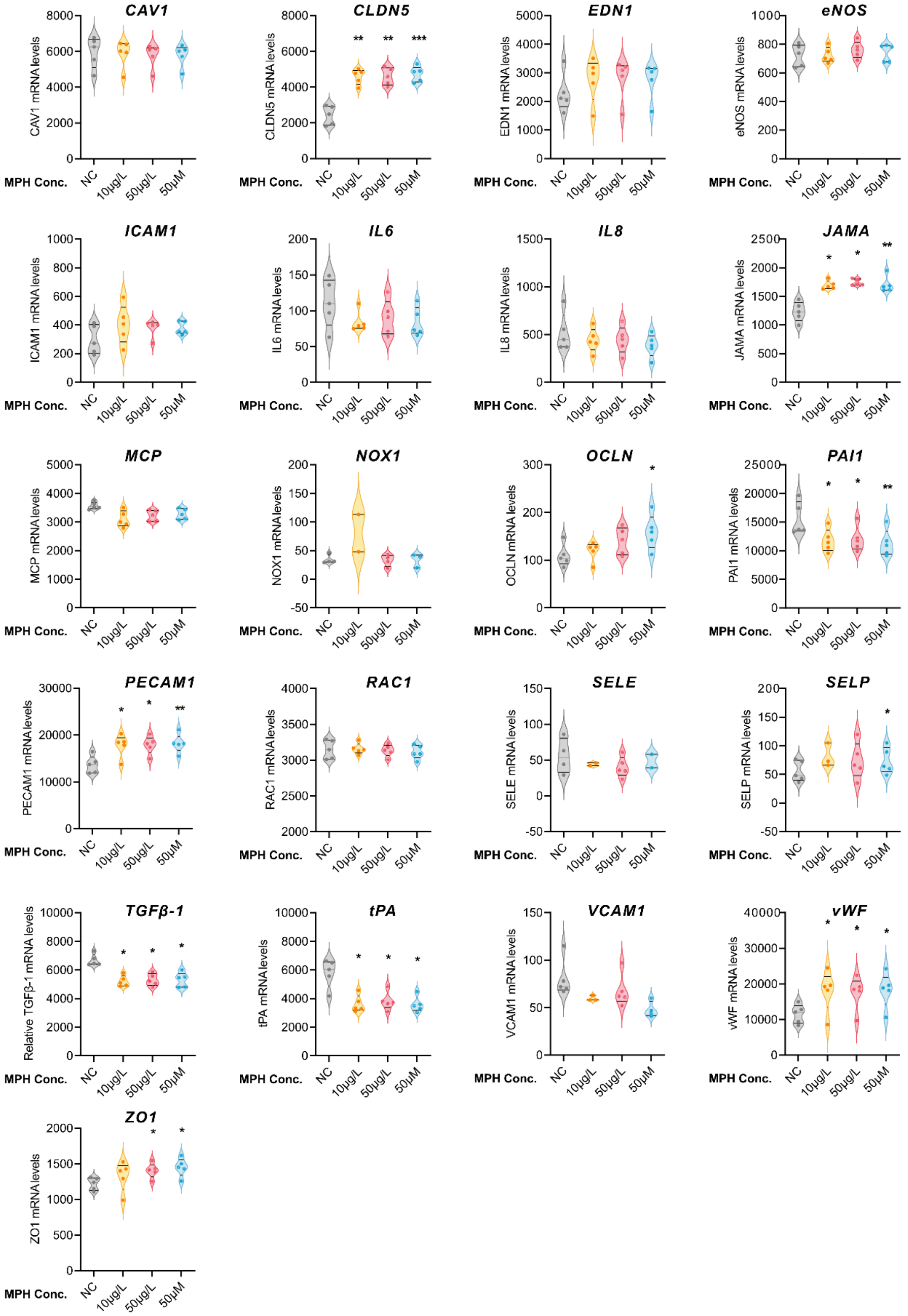

Supplementary Figure S2. Effects of MPH on mRNA expression of vascular endothelial function-related genes in human brain microvascular endothelial cells (HBEC). Violin plots showing absolute mRNA expression readout of 21 endothelial markers (*CAV1, CLDN5, EDN1, eNOS, ICAM1, IL6, IL8, JAMA, MCP, NOX1, OCLN, PAI1, PECAM1, RAC1, SELE,*

SELP, TGFβ-1, tPA, VCAM1, vWF, and *ZO1*) in HBEC following 24 h treatment with 10 μg/L, 50 μg/L, or 50 μM MPH. RNA sequencing was performed on five independent biological replicates per group (n = 5), with each replicate derived from an independent experiment. No technical replicates were included. Each dot represents one independent biological replicate. Statistical comparisons between each treatment group and NC were performed using paired *t*-tests, followed by FDR correction. Adjusted *p*-values are indicated. *adjusted $P < 0.05$, ** adjusted $P < 0.01$, *** adjusted $P < 0.001$.

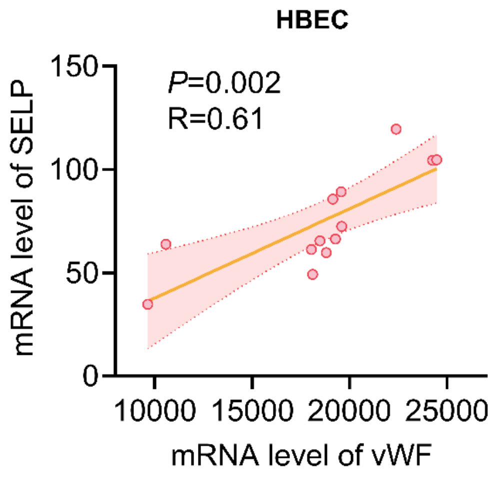

Supplementary Figure S3. Correlation between *vWF* and *SELP* mRNA expression levels in all samples from the treatment group in human brain microvascular endothelial cells (HBEC). Each dot represents an independent biological replicate (N=13) measured using nCounter Analysis System, including samples exposed to MPH at 10 µg/L (n = 3; 2 samples were excluded due to >30% of *SELP* values below the LLOD), 50 µg/L (n = 5), and 50 µM (n = 5) for 24 hours.

The correlation was assessed using Pearson correlation analysis. The orange line represents the best-fit linear regression, and the shaded area indicates the 95% confidence interval.

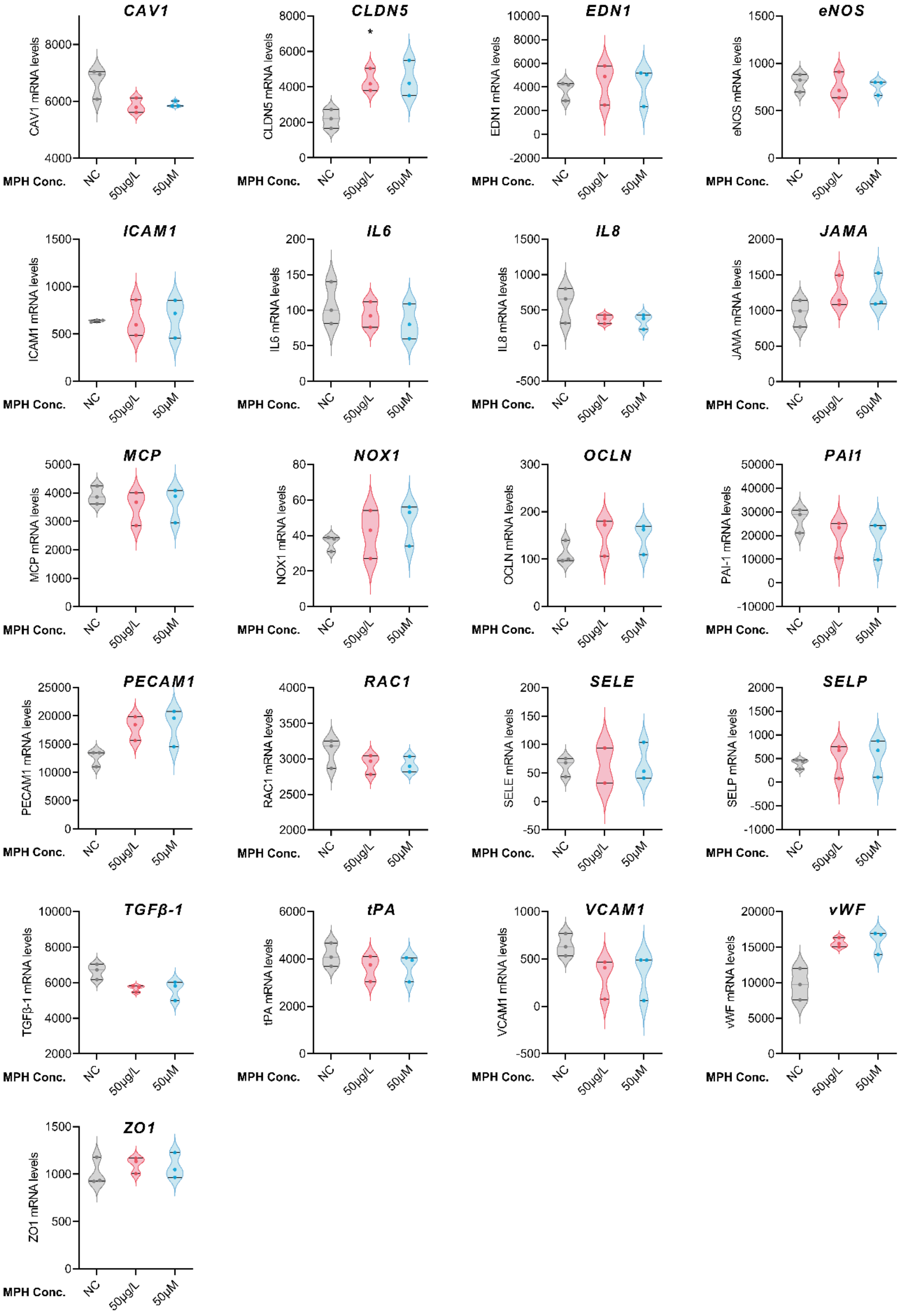

Supplementary Figure S4. Effects of MPH on mRNA expression of vascular endothelial function-related genes in human aortic endothelial cells (HAEC). Violin plots showing

absolute mRNA expression readout of 21 endothelial markers (*CAV1, CLDN5, EDN1, eNOS, ICAM1, IL6, IL8, JAMA, MCP, NOX1, OCLN, PAI1, PECAM1, RAC1, SELE, SELP, TGFβ-1, tPA, VCAM1, vWF,* and *ZO1*) in HAEC following 24 h treatment with 50 µg/L or 50 µM MPH. RNA sequencing was performed on three independent biological replicates per group (n = 3), with each replicate derived from an independent experiment. No technical replicates were included. Each dot represents one independent biological replicate. Statistical comparisons between each treatment group and NC were performed using paired *t*-tests, followed by FDR correction. Adjusted *p*-values are indicated. *adjusted $P<0.05$

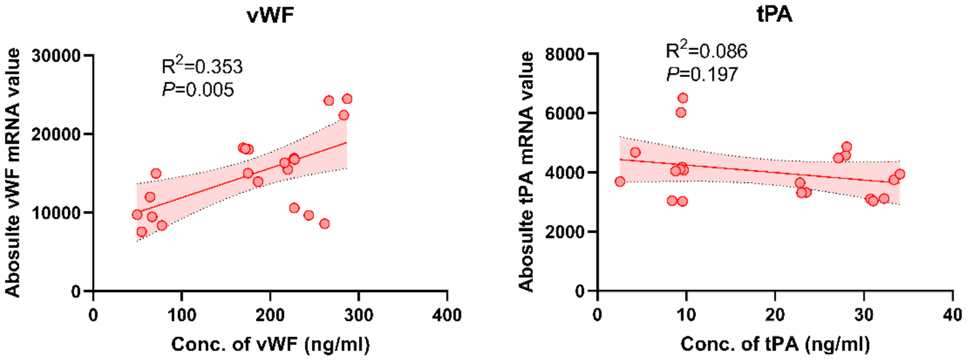

Supplementary Figure S5. Correlation between mRNA readouts and secreted protein levels, including all samples of human brain microvascular endothelial cells (HBEC) and human aortic endothelial cells (HAEC). Each dot represents an independent biological replicate (n = 21) measured using the nCounter Analysis System and ELISA, including NC, 10 µg/L, 50 µg/L, and 50 µM groups for HBEC (n = 3 for each group); NC, 50 µg/L, and 50 µM groups for HAEC (n = 3 for each group). Correlation was assessed using Pearson correlation analysis. The red line represents the best-fit linear regression, and the shaded area indicates the 95% confidence interval.

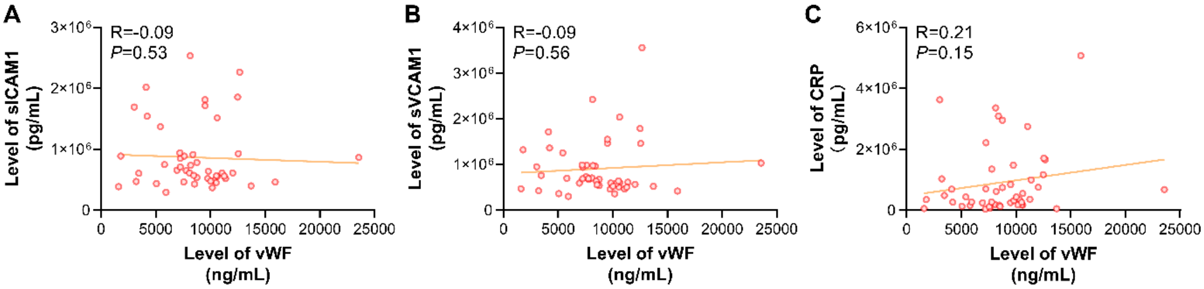

Supplementary Figure S6. Correlation for plasma levels of vWF with sICAM1, sVCAM1 and CRP in ADHD children (n=48). Each dot represents an independent participant's sample. Spearman correlation coefficients (R) and corresponding *P* values are shown in each panel. The orange line represents the best-fit linear regression. *Y*-axes represent analyte levels assessed using Meso Scale Discovery VPLEX Vascular Injury Panel 2 Human Kit (**Supplementary Method 10**). 17 of the children were currently on MPH, 14 were currently on non-MPH medication, and 17 were medication-naïve. 13 were female, 35 were male, and the mean±SD of age was 12.7±2.8.

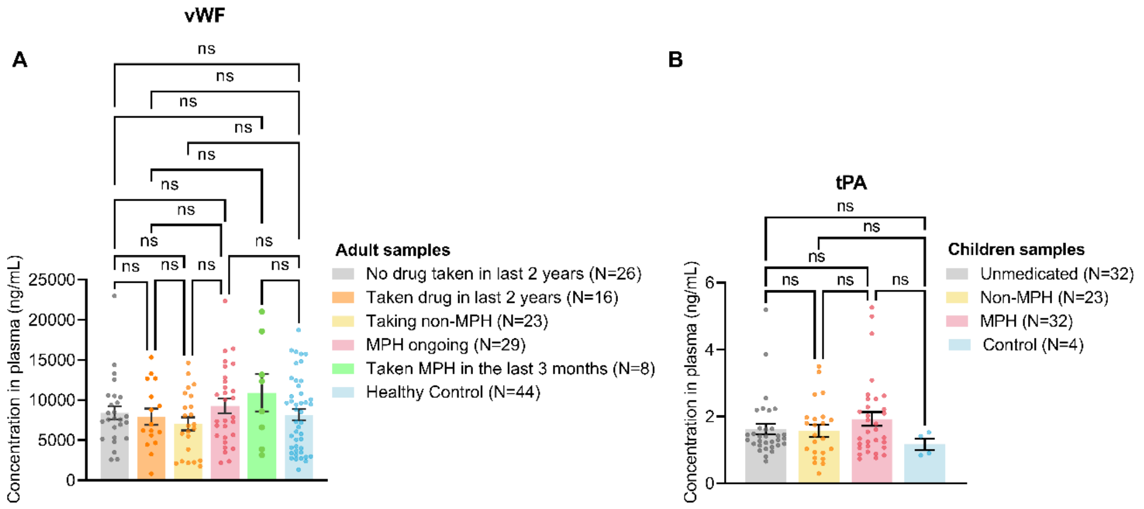

Supplementary Figure S7. Plasma protein levels of tPA and vWF in participants. (A) Plasma protein levels of vWF in adults with ADHD and controls. Each dot represents one independent participant's sample. Bars represent mean and error bars represent SD. Statistical analysis was performed using one-way ANOVA, following Dunn's multiple comparisons test. Unmedicated in Figure 1H (n=42) were categorised into 2 groups: i) no medication in the last 24 months (grey bar, n=26) and ii) no medication 3-24 months before sampling (orange bar, n=16). Non-MPH (yellow bar, n=23), the participants were on ADHD medication other than MPH currently or in the last 3 months, including Lisdexamphetamine only (n=15), Dexamphetamine only (n=3) and n=5 used both Lisdexamphetamine and Dexamphetamine. MPH in Figure 1H (n=37) were categorised into 2 groups: i) current MPH use (pink bar, n=29), and ii) MPH use in the last 3 months (green bar, n=8).

(B) Plasma protein levels of tPA in children with ADHD and controls. Each dot represents one independent participant's sample. Bars represent mean and error bars represent SEM. Statistical analysis was performed using Kruskal-Wallis test, following Tukey's multiple comparisons test. Unmedicated: the participants had never taken any ADHD medication (Drug-naïve). Non-MPH: the participants were currently on ADHD medication other than MPH, including Lisdexamphetamine only (n=18), Dextroamphetamine only (n=1), Atomoxetine only (n=3) and n=1 was on Lisdexamphetamine and Atomoxetine. MPH: the participants were currently on MPH (n=28 took MPH only and n=4 took MPH and Atomoxetine).

ns: not significant.

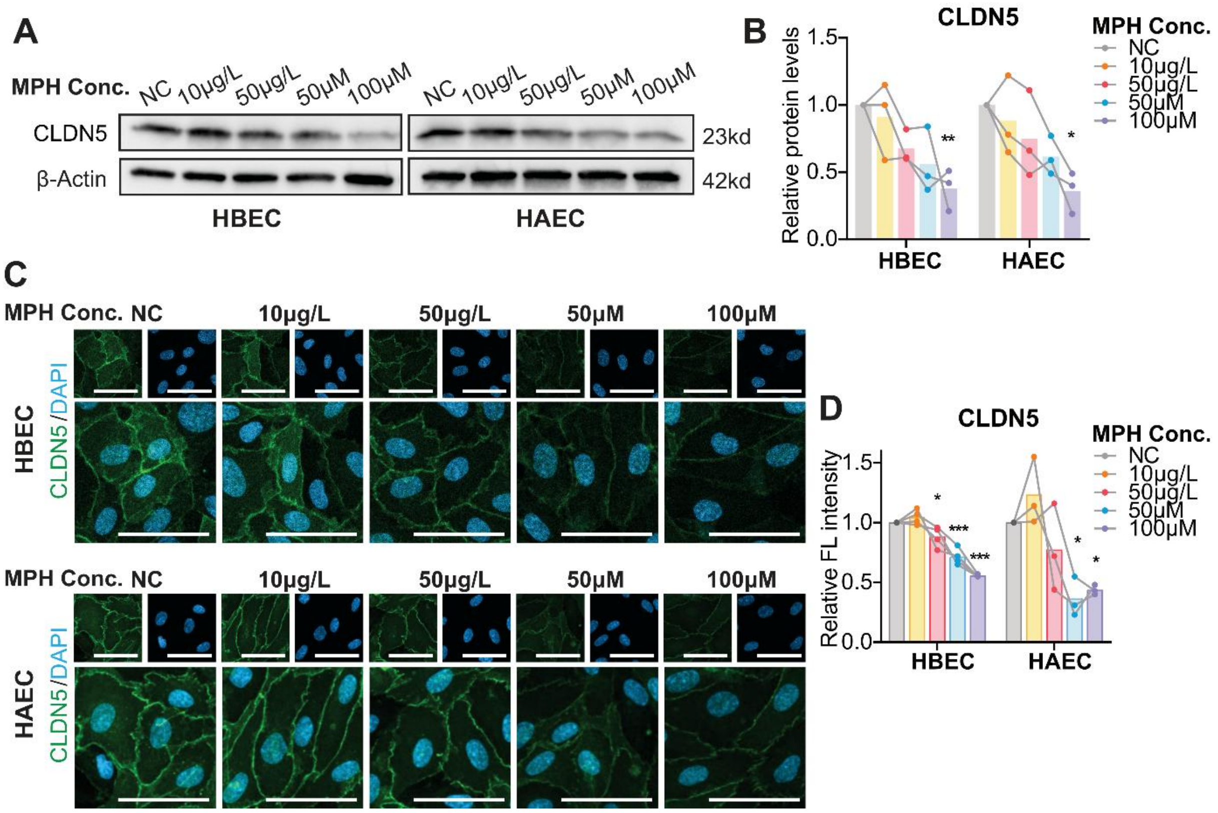

Supplementary Figure S8. MPH at supratherapeutic concentrations reduces CLDN5 protein expression in human vascular endothelial cells after 48 hours. (A) Representative Western blot images of total CLDN5 protein level in HBEC and HAEC following 48-hour treatment with MPH. (**B)** Quantification of relative CLDN5 protein levels from Western blots, normalised to β-actin and negative control (NC, 0μg/L MPH) within each experiment. Each dot represents an independent biological replicate, with no technical replicates. Each grey line connects data points obtained from the same independent experiment (n=3). (**C)** Representative immunocytochemistry image of CLDN5 (green) and nuclei (DAPI, blue) in HBEC and HAEC following 48-hour treatment with MPH. Scale bars: 50 μm. **(D)** Quantification of the total fluorescence intensity of CLDN5 membrane staining, normalised to cell number and NC within each experiment. Each dot represents an independent biological replicate. Each grey line connects data points obtained from the same independent experiment (n=4 for HBEC and n=3 for HAEC). Statistical analysis was performed using one-way ANOVA followed by Dunnett's post hoc test for multiple comparisons, comparing each treatment group to NC. *adjusted $P<0.05$, **adjusted $P<0.01$, *** adjusted $P<0.001$. Absolute values are provided in the supplementary raw data.

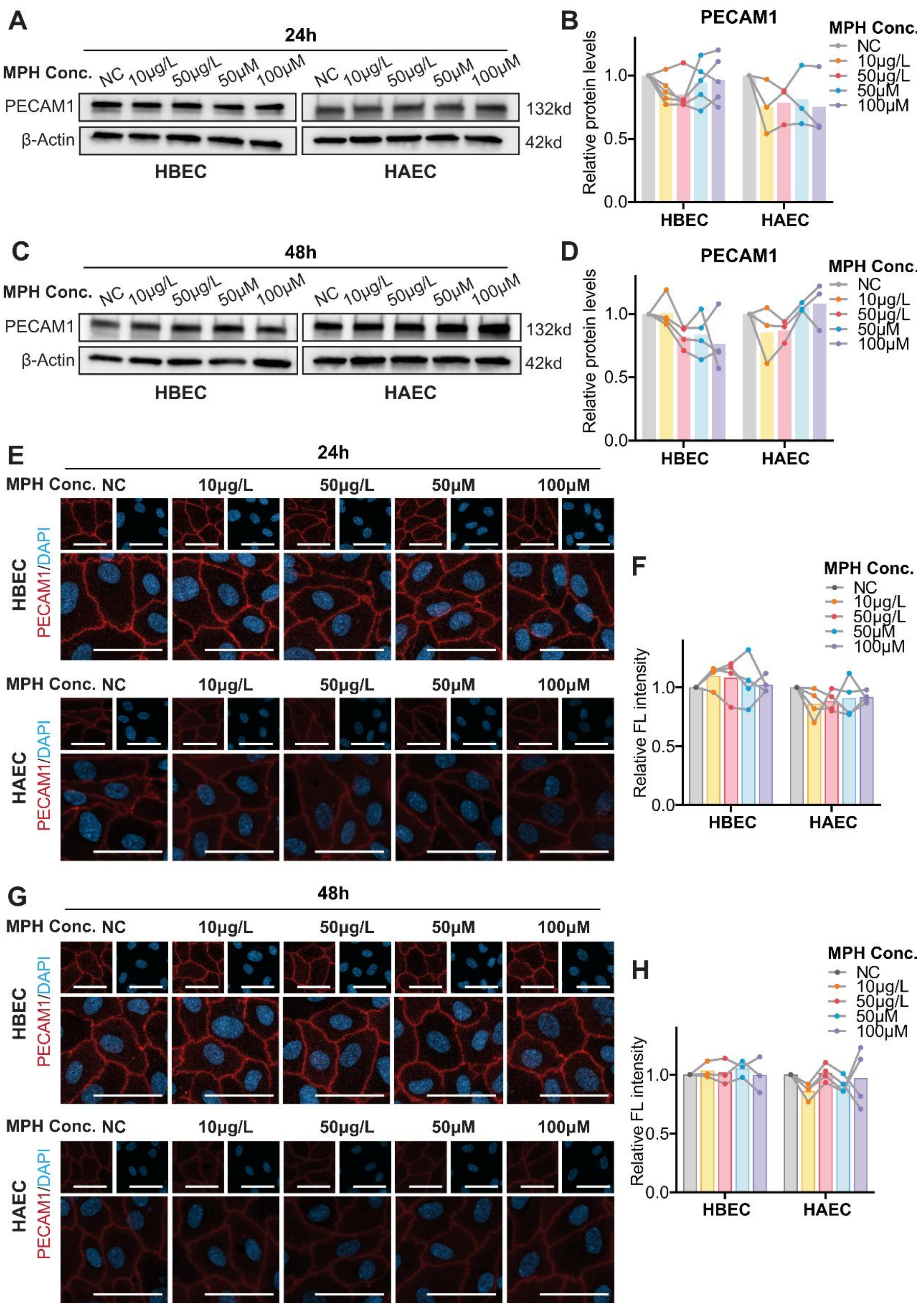

Supplementary Figure S9. MPH treatment does not significantly alter PECAM1 protein expression in human vascular endothelial cells. (A, C) Representative Western blot images of total PECAM1 protein level in HBEC and HAEC following 24-hour treatment (A), and 48-hour treatment (C) with MPH. **(B, D)** Quantification of relative PECAM1 protein levels from Western blots, normalised to β-actin and negative control (NC, 0μg/L MPH) within each

experiment. Each dot represents an independent biological replicate, with no technical replicates. Each grey line connects data points obtained from the same independent experiment (n=4-5 for HBEC and n=3 for HAEC). **(E, G)** Representative immunofluorescence images of PECAM1 (red) and nuclei (DAPI, blue) in HBEC and HAEC following MPH treatment for 24h (E), and 48h (G). Scale bars = 50 µm. **(F, H)** Quantification of the total fluorescence intensity of PECAM1, normalised to cell number and NC within each experiment. Each dot represents an independent biological replicate. Each grey line connects data points obtained from the same independent experiment (n=3-4 for HBEC and n=4 for HAEC). Statistical analysis was performed using one-way ANOVA followed by Dunnett's post hoc test for multiple comparisons, comparing each treatment group to NC. Absolute values are provided in the supplementary raw data.

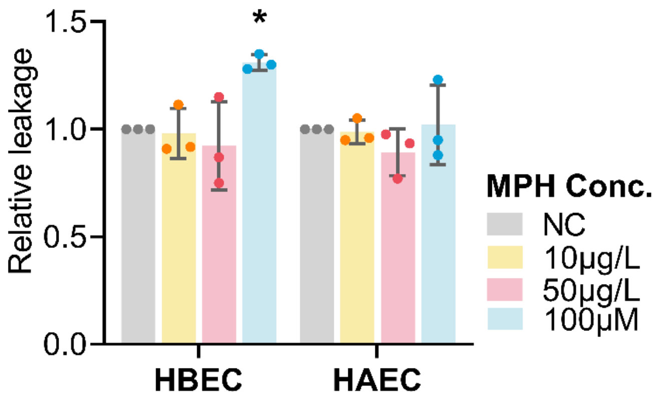

Supplementary Figure S10. MPH at supratherapeutic concentrations impairs the human vascular endothelial cell barrier (pilot study of Figure 3A, with only NC, 10 µg/L, 50 µg/L and 100 µM).

Quantification of paracellular permeability (70 kDa FITC-dextran) assessment using transwell plates in HBEC and HAEC after 24-hour treatment with MPH. Leakage levels were normalised to the corresponding negative control (NC, 0µg/L MPH) within each experiment. Each dot represents an independent biological replicate (n=3) with technical triplicates.

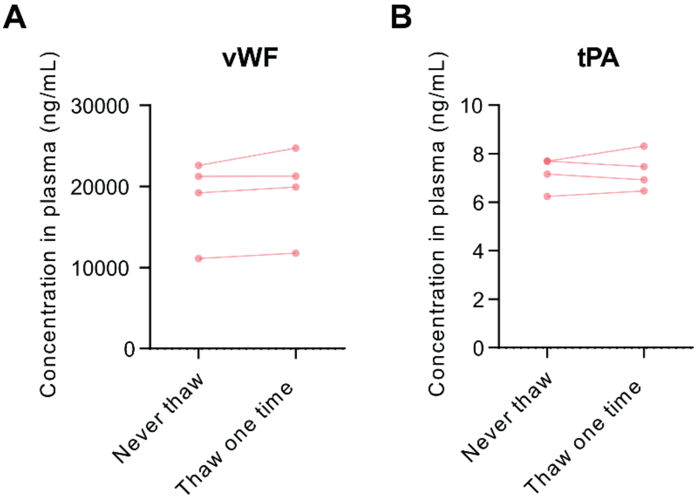

Supplementary Figure S11. Effect of a single freeze-thaw cycle on plasma von Willebrand factor (vWF) and tissue plasminogen activator (tPA) concentrations. Plasma concentrations of vWF (**A**) and tPA (**B**) measured before and after a single freeze-thaw cycle in four plasma samples using ELISA.

Supplementary Tables

Supplementary Table S1. Methylphenidate (MPH) concentrations and references behind their selection in this study.

µg/L	µM	nM	Reference
10.0 µg/L	0.0371 µM	37.1 nM	Chermá, et al. 2017; Marchei, et al. 2010; Markowitz, et al. 2003; Swanson and Volkow 2003
50.0 µg/L	0.185 µM	185 nM	Stevens, et al. 2010
13 488 µg/L	50.0 µM	50 000 nM	Coelho-Santos, et al. 2016; Gopal, et al. 2007; Kong, et al. 2021
26 977 µg/L	100 µM	100 000 nM	

Supplementary Table S2. List of all genes in the nCounter panel studied for expression.

Gene symbol	Corresponding protein full name (Abbreviation)	% below lower limit of detection (LLOD)
AGTR2, equal to *Ang II*	Angiotensin II (Ang II)	83%
CAV1	Caveolin-1 (CAV1)	0%
CD40LG, equal to *CD40L*	CD40 ligand (CD40L)	99%
CLDN5	Claudin-5 (CLDN5)	0%
NOS3, equal to *eNOS*	Endothelial nitric oxide synthase (eNOS)	0%
EDN1	Endothelin-1 (EDN1)	0%
SELE	E-selectin (SELE)	14%
ICAM1	Intercellular adhesion molecule 1 (ICAM-1)	0%
IL1A	Interleukin 1α (IL1α)	50%
IL1B	Interleukin 1β (IL1β)	99%
IL6	Interleukin 6 (IL6)	4%
CXCL8, equal to *IL8*	Interleukin 8 (IL8)	0%
F11R, equal to *JAM-A*	Junctional adhesion molecule A (JAM-A)	0%
CD46, equal to *MCP*	Monocyte chemoattractant protein-1 (MCP)	0%
MADCAM1	Mucosal addressin cell adhesion molecule-1 (MadCAM-1)	83%
NOX1	NADPH oxidase 1 (NOX1)	28%
CYBB, equal to *NOX2*	NADPH oxidase 2 (NOX2)	99%
OCLN	Occludin (OCLN)	0%
SERPINE1, equal to *PAI-1*	Plasminogen activating inhibitor-1 (PAI-1)	0%
PECAM1	Platelet endothelial cell adhesion molecule-1 (PECAM-1)	0%
SELP	P-selectin (SELP)	2%
RAC1	Rac family small GTPase 1 (Rac1)	0%
TF	Tissue factor (TF)	88%
PLAT, equal to *tPA*	Tissue plasminogen activator (tPA)	0%
TGFB1, equal to *TGFβ-1*	Transforming growth factor β-1 (TGFβ-1)	0%
TNF	Tumor necrosis factor α (TNF-α)	84%
VCAM1	Vascular cell adhesion molecule 1 (VCAM-1)	3%
VWF, equal to v*WF*	Von Willebrand factor (vWF)	0%
TJP1, equal to *ZO-1*	Zonula occludens-1 (ZO-1)	0%
**House-keeping genes**		
ACTB	Beta-actin (ACTB)	0%
B2M	Beta-2-microglobulin (B2M)	0%
GAPDH	Glyceraldehyde-3-phosphate dehydrogenase (GAPDH)	0%
GUSB	Beta-glucuronidase (GUSB)	0%
HPRT1	Hypoxanthine-guanine phosphoribosyltransferase (HPRT1)	0%
RPLP0	Ribosomal protein lateral stalk subunit P0 (RPLP0)	0%

LLOD was set as the mean plus two standard deviations of the negative controls. Markers with more than 30% of cell samples below LLOD were excluded from subsequent statistical analysis.

Supplementary Table S3. Associations between methylphenidate exposure and plasma vWF and tPA in multivariable linear regression models in children.

		vWF			tPA		
		**β**	**95% CI**	**P**	**β**	**95% CI**	***P***
**Model 1**							
Groups	Unmedicated (Reference)						
	MPH	0.259	**0.025-0.493**	**0.031**	0.092	-0.169-0.352	0.49
	Non-MPH	0.164	-0.098-0.427	0.22	-0.142	-0.434-0.150	0.34
	Control	-0.329	-0.880-0.223	0.24	-0.442	-1.056-0.172	0.16
ADHD symptom score		0.049	0.136-0.233	0.60	-0.129	-0.335-0.076	0.21
**Model 2**							
Groups	Unmedicated (Reference)						
	MPH	0.270	**0.037-0.503**	**0.024**	0.094	-0.163-0.351	0.47
	Non-MPH	0.151	-0.110-0.412	0.25	-0.114	-0.402-0.174	0.43
	Control	-0.322	-0.872-0.228	0.25	-0.405	-1.012-0.202	0.19
ADHD symptom score		0.073	-0.117-0.262	0.45	-0.124	-0.333-0.085	0.24
Sex_Male		0.182	-0.035-0.399	0.099	-0.276	-0.515--0.037	0.024
Age		0.027	-0.012-0.066	0.17	-0.011	-0.054-0.033	0.63

Model 1 was adjusted for the SNAP total score. Model 2 was further adjusted for sex and age.

Confidence intervals (CIs) are 95%. P values were derived from multivariable linear regression models.

Unmedicated (n=32): the ADHD patients had never taken any ADHD medication (Medication-naïve).

Non-MPH (n=23): the ADHD patients were currently on ADHD medication other than MPH, including Lisdexamphetamine only (n=18), Dextroamphetamine only (n=1), Atomoxetine only (n=3) and n=1 was on Lisdexamphetamine and Atomoxetine.

MPH (n=32): the ADHD patients were currently on MPH (n=28 took MPH only and n=4 took MPH and Atomoxetine).

Control (n=4): the participants without ADHD.

ADHD symptom scale: Parent-reported SNAP-IV.

Supplementary Table S4. Associations between methylphenidate exposure and plasma vWF and tPA in multivariable linear regression models in adults.

		vWF		
		**β**	**95% CI**	**P**
**Model 1**				
Groups	Unmedicated (Reference)			
	MPH	0.132	-0.152-0.416	0.36
	Non-MPH	-0.215	-0.552-0.122	0.21
	Control	-0.059	-0.331-0.213	0.67
Sex_Male		0.019	-0.201-0.24	0.86
**Model 2**				
Groups	Unmedicated (Reference)			
	Non-MPH	0.141	-0.144-0.425	0.33
	MPH	-0.225	-0.566-0.115	0.19
	Control	-0.048	-0.323-0.227	0.73
Sex_Male		0.017	-0.205-0.238	0.88
Age		-0.004	-0.018-0.01	0.53
BMI		-0.011	-0.035-0.013	0.35
**Model 3**				
Groups	Unmedicated (Reference)			
	Non-MPH	0.132	-0.153-0.417	0.36
	MPH	-0.249	-0.59-0.092	0.15
	Control	-0.128	-0.497-0.24	0.49
Sex_Male		0.0309	-0.194-0.256	0.79
Age		-0.0057	-0.02-0.009	0.44
BMI		-0.0123	-0.036-0.012	0.31
ASRS_Total_mean		-0.0599	-0.254-0.134	0.54

Model 1 was adjusted for sex. Model 2 was further adjusted for age and BMI. Model 3 was further adjusted for age, BMI and ADHD symptom score.

Confidence intervals (CIs) are 95%. P values were derived from multivariable linear regression models.

Unmedicated (n=42): the ADHD participants had no ADHD medication in the last 3 months.

Non-MPH (n=23): the ADHD participants were on ADHD medication other than MPH currently or in the last 3 months, including Lisdexamphetamine only (n=15), Dexamphetamine only (n=3) and n=5 used both Lisdexamphetamine and Dexamphetamine.

MPH (n=37): the ADHD participants use MPH currently or in the last 3 months (n=33 use MPH only, n=3 use MPH and Lisdexamphetamine and n=1 uses MPH and Atomoxetine). Control (n=44): the participants without ADHD.

ADHD symptom scale: Self-reported ASRS.

Supplementary methods

1. Cell culture and treatments

Human aortic endothelial cells (HAECs) were obtained from a 56-year-old male donor. Human brain endothelial cells (HBECs) were derived from a normal, healthy male pediatric donor. HAEC and HBEC were seeded onto gelatin-coated (Cat.#G9391, Sigma-Aldrich, St. Louis, MO, USA) plates and maintained at 37°C with 5% CO_2. The medium was changed every two days until the cells became confluent. All experiments were performed using cell passages three to seven, and the absence of *Mycoplasma* contamination was confirmed using a mycoplasma detection kit (Cat.#rep-mys-100; InvivoGen, Hong Kong, China). MPH was freshly prepared for each experiment and protected from light. It was dissolved in sterile water to prepare a 1mg/ml stock solution and subsequently filtered using a sterile membrane filter prior to use. The stock solution was further diluted in working solutions. Equal volumes of working solution were added to the culture medium to achieve the indicated final concentrations. Control cells received an equivalent volume of sterile water.

2. Cell proliferation and viability assays

The cells were seeded in a transparent 96-well plate at a density of 5000 cells/well under a series of concentration gradients: 0 µg/L MPH (Negative control, NC), 10 µg/L MPH, 50 µg/L MPH, and 13488 µg/L (equivalent to 50µM MPH). Cells were treated once with MPH and monitored for up to 48 hours. Previous studies have reported that 100 µM MPH does not significantly affect endothelial cell viability [1]. 200 000 µg/L (equivalent to 741 µM MPH) was set as a high-dose cytotoxic control to confirm the sensitivity of the proliferation and cell death assays. The IncuCyte Live-cell Analysis System (Sartorius, Göttingen, Germany) captured five images of each well every 4 hours over a period of 48 hours. To evaluate cell proliferation, the confluence was automatically analysed using the IncuCyte software, version 2022A (Sartorius), and normalised to the baseline value obtained at zero hours. Cell death was quantified using fluorescence-based detection with IncuCyte Cytotox Dye (Cat.#4632, Sartorius).

3. Study participants and blood sampling

The participants in this study were persons between 8 and 51 years of age, with a confirmed ADHD diagnosis (ICD-10 F90), or without an ADHD diagnosis defining the healthy controls. The healthy controls were either family or friends of a participant with ADHD, or completely unrelated to the other participants. None had autism, intellectual disability, eating disorder, diabetes or any gastrointestinal diagnosis. None had changed treatment in the past 4 weeks,

none had taken antibiotics in the past 6 weeks and none had any symptom of infection. Medication details are found in Table 1. At baseline, non-fasting venous blood was collected in EDTA tubes, which were immediately centrifuged at 1700 g (3500 rpm) for 20 minutes. Subsequently, the plasma was aliquoted and stored at -80°C until analysis. All samples had been exposed to one additional freeze-thawing cycle before analysis.

4. Gene expression analysis

The cells were incubated with different concentrations of MPH for 24 hours before collection. For HBEC, four different concentrations of MPH were used (NC, 10 µg/L, 50 µg/L, and 50 µM), with five biological replicates in each group. HAEC was used as a supplementary validation, with three MPH concentrations (NC, 50 µg/L, and 50 µM), each containing three biological replicates. Cell samples were processed in batches of 12 samples per run of 8 runs. Cultured cells were lysed using Buffer RLT (Cat.#79216, QIAGEN, Hilden, Germany) and then hybridised overnight at 65°C with the pre-designed nCounter CodeSet targeting genes of interest. After hybridisation, samples were processed on the automated nCounter Prep Station and analysed using the nCounter Digital Analyzer.

Initially, raw count data were subjected to quality control (QC) procedures assessing imaging quality (percentage of fields of view [FOV] successfully imaged >75%), binding density (optimal range 0.1–2.25 spots/µm²), and positive control linearity (R^2 >0.95). Samples failing any of these QC criteria were excluded from subsequent analyses. The raw mRNA expression data passing QC were first normalised by the geometric mean of internal positive controls in the panel and then further normalised by the geometric mean of a set of stably expressed housekeeping genes (*ACTB, B2M, GAPDHGUSB, HPRT1* and *RPLP0*) using nSolver software (version 4.0, NanoString Technologies). Values below the background expression (lower level of detection [LLOD]) were filtered out from the analysis using the mean plus two standard deviations of negative control counts in the panel (**Supplementary Table 1**).

The genes were subsequently tested statistically using paired t-tests, applying a False Discovery Rate (FDR) correction. Fold changes (FC) between treatment and control groups were calculated. Only genes with FDR-adjusted *P* value ≤0.05, and absolute fold-change ≥1.5 or ≤0.67 were selected as biomarkers for subsequent protein verification.

5. Enzyme-linked immunosorbent assay (ELISA) for vWF and tPA

Concentrations of vWF and tPA in cell culture supernatants and blood plasma were determined using a commercially available 96-well-format ELISA kit (Human vWF ELISA Kit, Cat.#EHVWF, Human tPA ELISA Kit, Cat.#BMS258-2, Thermo Fisher Scientific), according to the manufacturer's protocol. Phosphoric acid (1 M) was used as stop solution to avoid precipitation. Cell supernatants were collected from the same independent experiments as the mRNA analyses, aliquoted, and stored at -80 °C without repeated freeze-thaw cycles prior to ELISA analysis. The cell supernatants were diluted 1:32 for both vWF and tPA. Blood plasma samples were diluted 1:1000 and 1:5 for vWF and tPA, respectively. In total, 4 plates of each analyte were run for children, with equal distribution of the groups on each plate, plus a standard curve and two plasma samples included on all plates as inter-plate controls. All samples were run in duplicate, and each well was prepared separately. Similarly, the adult samples were run on 4 plates, but only for vWF. The ELISA assay for tPA had a lower limit of detection of 6 pg/mL. According to the manufacturer's specifications, no significant cross-reactivity was observed with other circulating proteins, including plasminogen activator inhibitor-1 (PAI-1), at concentrations up to 2000 pg/mL. The ELISA assay for vWF had a lower limit of detection of 0.13 ng/mL. The antibody pair specifically detects human vWF, with no reported cross-reactivity under the tested conditions. All samples had detectable levels. The inter-plate and intra-plate coefficients of variation for all the plasma samples were for vWF: 10.6% and 2.1%, and for tPA: 14.0% and 4.1%, respectively. No potential effect of freeze-thawing was detected (**Supplementary Figure S11**).

6. Western blot analysis

Cells were harvested and lysed using RIPA buffer (Cat.#89900, Thermo Fisher Scientific) supplemented with protease inhibitors (Cat.# 04693124001, Roche, Basel, Switzerland). Protein concentrations were quantified using a BCA assay (Cat.#23227, Thermo Fisher Scientific). Equal amounts of total protein were loaded for Western blot analysis. Protein samples supplemented with Sample Reducing Agent (Cat.#11569166, Thermo Fisher Scientific) and Laemmli Sample Buffer (Cat.#1610747, Bio-Rad, Hercules, CA, USA) were denatured at 98 °C for 3 minutes. Protein samples (40 µg) were run on a 4-20% precast polyacrylamide gel (Cat.#4561094, Bio-Rad) and then transferred onto polyvinylidene difluoride membranes (Cat.#1704156, Bio-Rad). Membranes were blocked in 5% non-fat milk in Tris-buffered saline with 0.05% Tween (TBS-T, Cat.#28358, Thermo Fisher Scientific) for one hour at room temperature, followed by incubation with primary antibodies at 4°C overnight. After washing, membranes were incubated with secondary antibodies for one hour at room temperature. A pre-stained protein molecular weight marker (Cat.#1610375,

Bio-Rad; Cat.# 26634, Thermo Fisher Scientific) was used as a size reference for protein band identification. Protein signals were visualised with enhanced chemiluminescence (Cat.#1705062, Bio-Rad) and detected using the ChemiDoc imaging system (Bio-Rad). Band intensities were quantified using ImageJ software.

7. Immunocytochemistry

Cells grown on a clear-bottom black 96-well plate (Cat.# CLS3603, Corning, Corning, NY, USA) were fixed with 4% paraformaldehyde or 95% ethanol at room temperature for 15 min for PECAM1 and CLDN5, respectively. The cells were then blocked with 5% donkey serum for one hour at room temperature and thereafter incubated overnight at 4°C with primary antibodies, followed by secondary antibodies for one hour at room temperature in the dark. Cell nuclei were counterstained with DAPI for 5 min at room temperature. Images were acquired using LSM 900 Confocal microscope (Carl Zeiss, Oberkochen, Germany), maintaining identical acquisition settings for all samples. Fluorescence intensity was assessed using ImageJ software for quantitative analysis. Quantification was performed as total fluorescence intensity per image normalized to cell number.

8. Fluorescence-activated cell sorting (FACS)

After rinsing twice with PBS and once with Versene (Cat.#15040066, Thermo Fisher Scientific), cells were detached by adding trypsin (Cat.#R001100, Thermo Fisher Scientific) for 1 min. The cells were washed twice in PBS and incubated with primary antibodies for 30 min at 4°C in the dark. The cells were then stained with fluorophore-conjugated secondary antibodies for 30 min at 4°C in the dark. Dead cells were labelled using the live/dead dye (Cat.#A1310, Thermo Fisher Scientific). After washing, cells were resuspended in staining buffer (PBS containing 3% FBS) and analysed with a fluorescence-activated cell sorter (FACSverse, BD Biosciences, San Jose, CA, USA). Data analysis was performed using FlowJo software (version 10.10.0, BD Biosciences). Flow cytometry data were analysed using a standardised gating strategy. Briefly, debris was excluded based on forward and side scatter (FSC-A vs SSC-A), followed by the selection of single cells using FSC-A vs FSC-H. Viable cells were identified using live/dead dye staining, and target protein expression was subsequently analysed within the gated population.

9. Permeability assessment in transwell plates *in vitro*

Cells were seeded in the upper chamber at a density of 50000 cells/well and then cultured for 5 days until confluent. Following a 24-hour treatment period with MPH, the upper and lower chambers of the Transwell were gently washed with PBS after careful removal of the medium. The upper chamber medium was replaced with non-MPH medium containing 70kDa

FITC-dextran (Cat.#90718, Sigma-Aldrich), and the lower chamber medium was replaced with non-MPH medium. Following one-hour incubation at 37°C, 100 µL of the medium was collected from the lower chamber and added to a black 96-well plate (Cat.#237105, Thermo Fisher Scientific). Fluorescence intensity was measured using a fluorescence microplate reader (SpectraMax iD3, Molecular Devices, San Jose, CA, USA, 493 nm and 520 nm excitation and emission, respectively). Permeability was quantified by calculating the fluorescence intensity of treatment groups relative to control groups.

10. Multiplex immunoassays for plasma levels of sICAM1, sVCAM1 and CRP

Plasma concentrations of sICAM1, sVCAM1 and CRP were quantified in April-May 2019 using a sandwich-based multiplex immunoassay (V-PLEX Vascular Injury Panel 2 Human Kit, Cat. #K15198D; Meso Scale Discovery, USA), following the manufacturer's protocol, as previously described [2].

Reference

1. Coelho-Santos, V., et al., *Methylphenidate-triggered ROS generation promotes caveolae-mediated transcytosis via Rac1 signaling and c-Src-dependent caveolin-1 phosphorylation in human brain endothelial cells.* Cell Mol Life Sci, 2016. **73**(24): p. 4701-4716.
2. Yang, L.L., et al., *Proinflammatory mediators and their associations with medication and comorbid traits in children and adults with ADHD.* Eur Neuropsychopharmacol, 2020. **41**: p. 118-131.
