# Supplementary material for: Effects of methylphenidate on the human vascular endothelium
Source: Transl Psychiatry. 2026 Jul 17;16:369. doi: 10.1038/s41398-026-04237-6 (PMC13379382; doi:10.1038/s41398-026-04237-6)
Supplement: Supplementary file 2 — Supplementary file 1_WB raw pictures [file 41398_2026_4237_MOESM2_ESM.pdf]

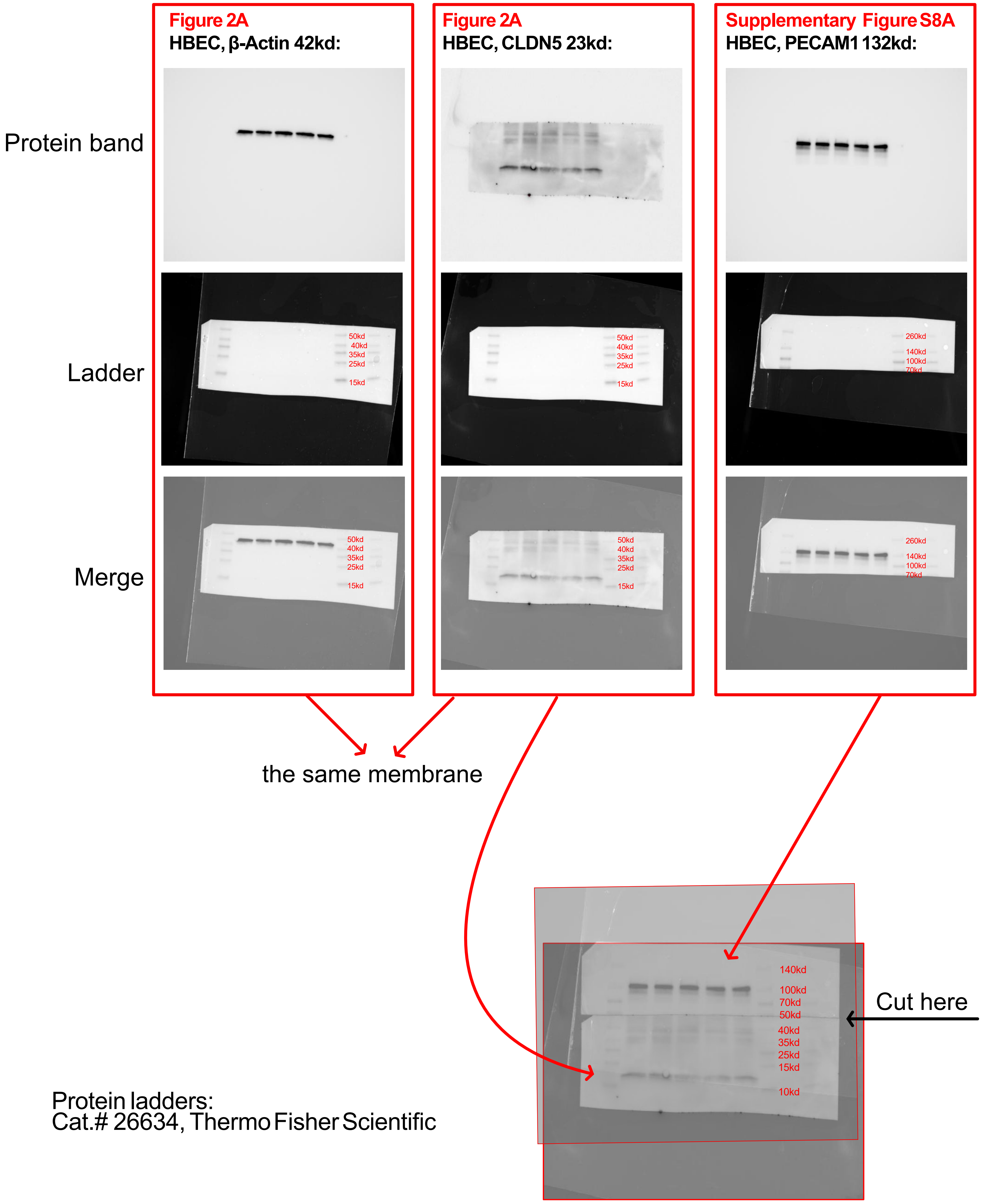

Figure 2A
HBEC, β-Actin 42kd:
Figure 2A
HBEC, CLDN5 23kd:
Supplementary Figure S8A
HBEC, PECAM1 132kd:
Protein band
Ladder
Merge
50kd
40kd
35kd
25kd
15kd
260kd
140kd
100kd
70kd
the same membrane
140kd
100kd
70kd
50kd
40kd
35kd
25kd
15kd
10kd
Cut here
Protein ladders:
Cat.# 26634, Thermo Fisher Scientific

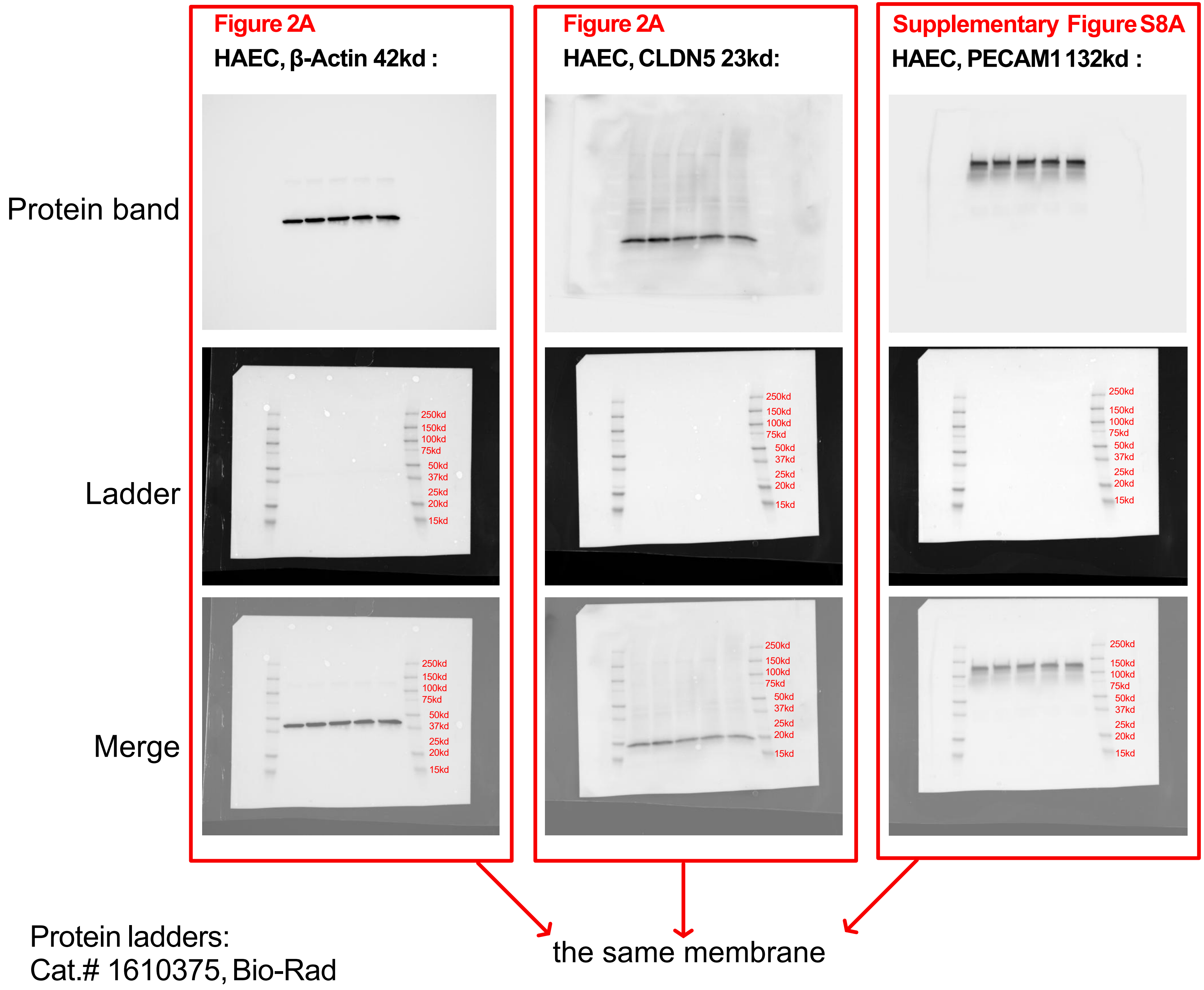

Figure 2A
HAEC, β-Actin 42kd :
Figure 2A
HAEC, CLDN5 23kd:
Supplementary Figure S8A
HAEC, PECAM1 132kd :
Protein band
Ladder
Merge
250kd
150kd
100kd
75kd
50kd
37kd
25kd
20kd
15kd
the same membrane
Protein ladders:
Cat.# 1610375, Bio-Rad

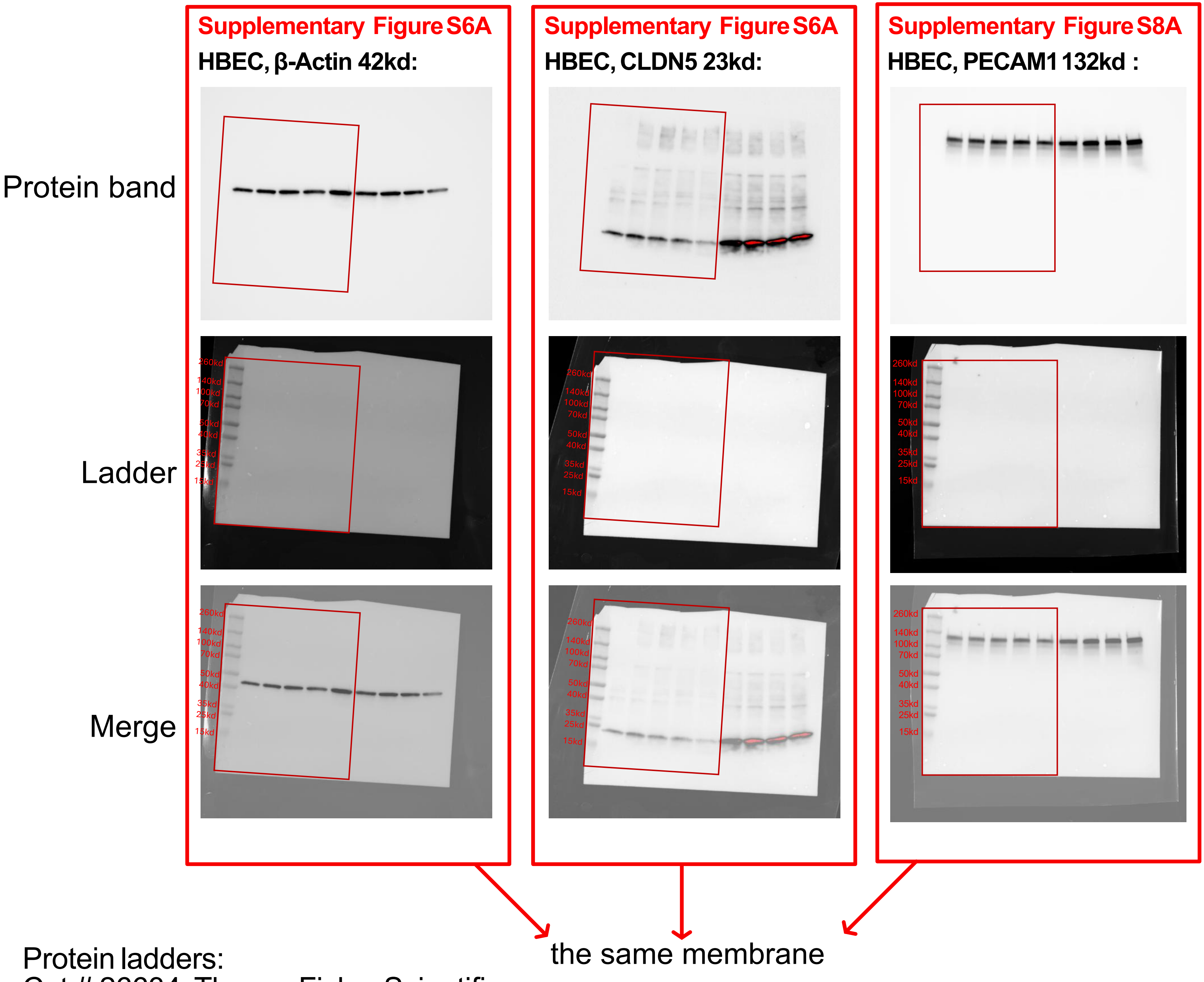

Protein ladders:
Cat.# 26634, Thermo Fisher Scientific

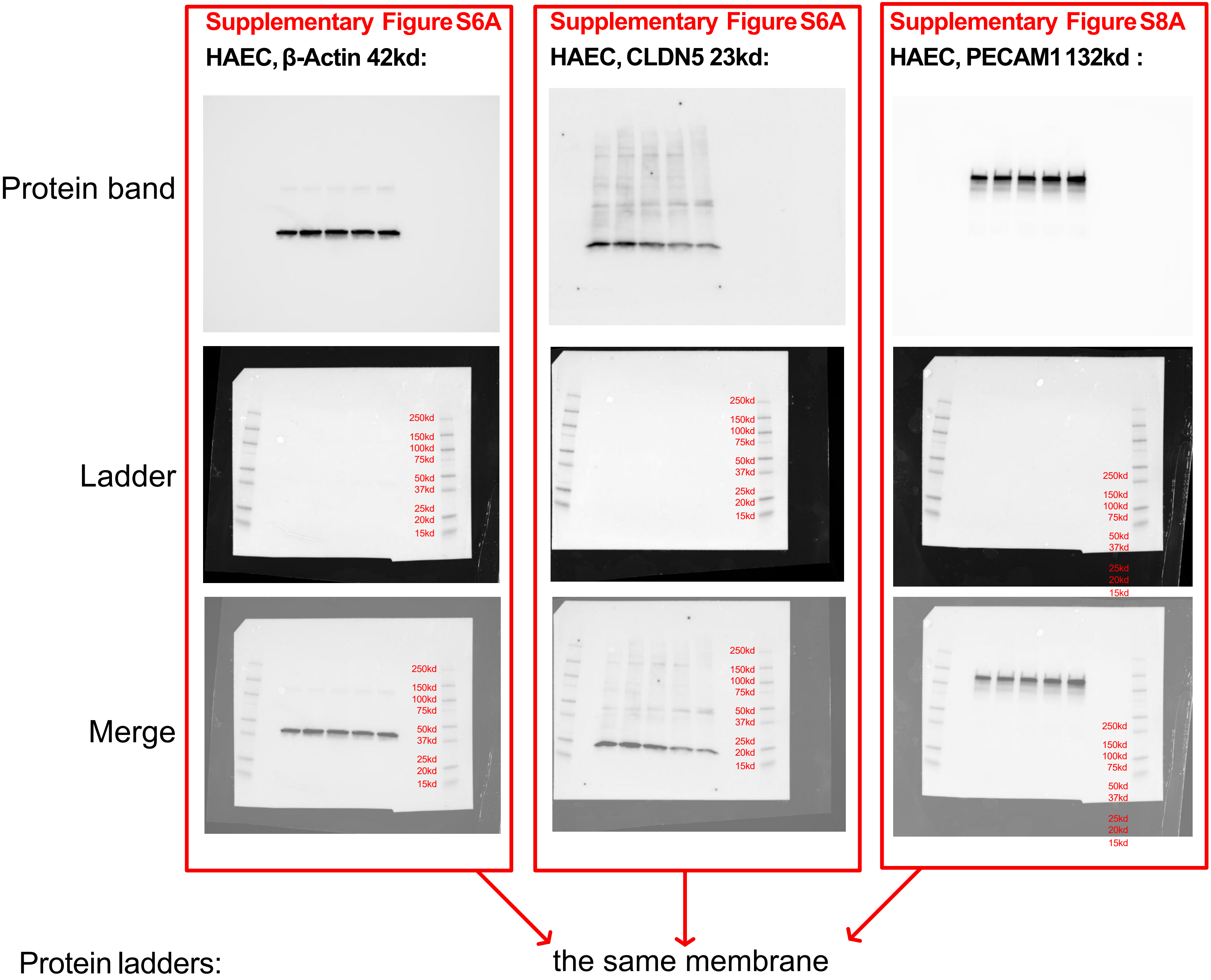

Protein ladders:
Cat.# 26634, Thermo Fisher Scientific
