# Supplementary material for: Effects of methylphenidate on the human vascular endothelium
Source: Transl Psychiatry. 2026 Jul 17;16:369. doi: 10.1038/s41398-026-04237-6 (PMC13379382; doi:10.1038/s41398-026-04237-6)
Supplement: Supplementary file 3 — Supplementary file 2_raw data [file 41398_2026_4237_MOESM3_ESM.pdf]

Table2&Fig1A-D&FigS2&FigS4

	HBEC												HAEC							
	10 µg/L MPH				50 µg/L MPH				50uM MPH				50 µg/L MPH				50uM MPH			
Marker name	Fold Change	Log_2 (Fold Change)	P-value	Adjusted P-value	Fold Change	Log_2 (Fold Change)	P-value	Adjusted P-value	Fold Change	Log_2 (Fold Change)	P-value	Adjusted P-value	Fold Change	Log_2 (Fold Change)	P-value	Adjusted P-value	Fold Change	Log_2 (Fold Change)	P-value	Adjusted P-value
CLDN5	1,89	0,92	0,0001	0,001	1,90	0,92	0,0001	0,002	1,95	0,97	0,00002	0,0004	1,98	0,99	0,002	0,045	2,01	1,01	0,016	0,185
JAM-A	1,38	0,46	0,002	0,013	1,42	0,50	0,002	0,015	1,38	0,47	0,001	0,006	1,28	0,36	0,247	0,432	1,29	0,36	0,249	0,374
tPA	0,63	-0,67	0,002	0,013	0,66	-0,59	0,003	0,015	0,62	-0,69	0,003	0,011	0,88	-0,19	0,032	0,169	0,89	-0,18	0,110	0,214
PAI-1	0,76	-0,40	0,003	0,013	0,78	-0,37	0,004	0,015	0,73	-0,46	0,001	0,006	0,73	-0,45	0,048	0,190	0,71	-0,50	0,045	0,191
PECAM-1	1,30	0,38	0,003	0,013	1,31	0,39	0,003	0,015	1,33	0,41	0,0005	0,005	1,42	0,50	0,010	0,105	1,45	0,53	0,036	0,191
TGFβ	0,78	-0,36	0,008	0,026	0,80	-0,33	0,010	0,026	0,80	-0,33	0,011	0,029	0,85	-0,23	0,021	0,149	0,85	-0,24	0,026	0,185
vWF	1,56	0,64	0,017	0,050	1,52	0,60	0,009	0,026	1,58	0,66	0,006	0,018	1,60	0,68	0,063	0,190	1,62	0,70	0,060	0,209
MCP	0,87	-0,20	0,029	0,077	0,90	-0,15	0,042	0,098	0,92	-0,12	0,037	0,071	0,90	-0,15	0,436	0,572	0,93	-0,10	0,525	0,648
ICAM-1	1,36	0,45	0,038	0,089	1,28	0,36	0,067	0,140	1,28	0,35	0,101	0,141	1,02	0,02	0,930	0,930	1,06	0,09	0,756	0,784
IL6	0,76	-0,40	0,121	0,253	0,81	-0,30	0,150	0,224	0,75	-0,41	0,064	0,106	0,87	-0,20	0,191	0,399	0,78	-0,36	0,021	0,185
VCAM1	0,74	-0,43	0,172	0,329	0,84	-0,25	0,339	0,375	0,60	-0,74	0,090	0,135	0,49	-1,02	0,063	0,190	0,54	-0,90	0,089	0,214
SELE	0,79	-0,35	0,198	0,339	0,72	-0,47	0,331	0,375	0,88	-0,19	0,737	0,773	1,02	0,03	0,815	0,856	1,06	0,09	0,784	0,784
ZO1	1,09	0,12	0,234	0,339	1,15	0,20	0,004	0,015	1,19	0,25	0,013	0,030	1,09	0,12	0,568	0,662	1,07	0,09	0,695	0,769
SELP	1,47	0,56	0,242	0,339	1,33	0,41	0,135	0,224	1,33	0,41	0,006	0,018	1,27	0,35	0,560	0,662	1,40	0,49	0,466	0,612
IL8	0,85	-0,24	0,249	0,339	0,85	-0,23	0,241	0,316	0,74	-0,44	0,066	0,106	0,63	-0,67	0,209	0,399	0,58	-0,77	0,126	0,214
EDN1	1,20	0,27	0,258	0,339	1,22	0,29	0,143	0,224	1,20	0,26	0,148	0,194	1,17	0,22	0,388	0,572	1,11	0,15	0,453	0,612
NOX1	2,37	1,25	0,365	0,451	0,96	-0,06	0,802	0,830	0,98	-0,02	0,813	0,813	1,16	0,22	0,415	0,572	1,32	0,41	0,123	0,214
OCLN	1,09	0,12	0,531	0,620	1,28	0,35	0,075	0,144	1,45	0,53	0,023	0,049	1,37	0,45	0,151	0,396	1,32	0,40	0,132	0,214
CAV1	0,99	-0,02	0,586	0,647	0,97	-0,05	0,260	0,322	0,97	-0,04	0,314	0,366	0,87	-0,19	0,203	0,399	0,88	-0,18	0,110	0,214
RAC1	1,00	0,00	0,915	0,960	1,00	-0,01	0,830	0,830	0,99	-0,02	0,652	0,721	0,95	-0,08	0,367	0,572	0,94	-0,09	0,117	0,214
eNOS	1,00	0,00	0,977	0,977	1,05	0,07	0,192	0,269	1,03	0,04	0,259	0,319	0,94	-0,09	0,754	0,833	0,94	-0,09	0,605	0,706

Fig1D&FigS2-3

		tPA		vWF		CLDN5		CAV1		EDN1		ICAM1		IL6	
Group	Experiments	Abosulte mRNA value	Relative value of NC	Abosulte mRNA value	Relative value of NC	Abosulte mRNA value	Relative value of NC	Abosulte mRNA value	Relative value of NC	Abosulte mRNA value	Relative value of NC	Abosulte mRNA value	Relative value of NC	Abosulte mRNA value	Relative value of NC
NC	Exp1	4176	1,00	9459	1,00	2484	1,00	5545	1,00	2311	1,00	191	1,00	110	1,00
	Exp2	6022	1,00	14983	1,00	2972	1,00	6772	1,00	2037	1,00	271	1,00	63	1,00
	Exp3	6510	1,00	8358	1,00	1938	1,00	4639	1,00	1597	1,00	215	1,00	136	1,00
	Exp4	5567	1,00	12800	1,00	1816	1,00	6252	1,00	3406	1,00	414	1,00	149	1,00
	Exp5	6628	1,00	12119	1,00	2895	1,00	6558	1,00	2107	1,00	391	1,00	97	1,00
	**Mean**	5781	1,00	11544	1,00	2421	1,00	5953	1,00	2292	1,00	296	1,00	111	1,00
	**SD**	991	0,00	2657	0,00	532	0,00	869	0,00	675	0,00	101	0,00	34	0,00
10ug/L MPH	Exp1	3114	0,75	18225	1,93	4843	1,95	6009	1,08	2641	1,14	226	1,18	76	0,69
	Exp2	3322	0,55	24454	1,63	5014	1,69	6387	0,94	3503	1,72	454	1,67	79	1,27
	Exp3	4577	0,70	8595	1,03	3940	2,03	4549	0,98	1489	0,93	336	1,57	110	0,81
	Exp4	3292	0,59	19591	1,53	4372	2,41	5935	0,95	2980	0,88	594	1,44	81	0,54
	Exp5	3800	0,57	19270	1,59	4770	1,65	6444	0,98	3173	1,51	407	1,04	75	0,77
	**Mean**	3621	0,63	18027	1,54	4588	1,94	5865	0,99	2758	1,24	403	1,38	84	0,82
	**SD**	592	0,09	5794	0,32	432	0,31	769	0,06	775	0,37	137	0,26	14	0,27
50ug/L MPH	Exp1	3100	0,74	18035	1,91	4993	2,01	5716	1,03	2886	1,25	271	1,42	64	0,58
	Exp2	3652	0,61	22380	1,49	5150	1,73	6080	0,90	3093	1,52	395	1,46	71	1,14
	Exp3	4863	0,75	9662	1,16	4010	2,07	4614	0,99	1545	0,97	399	1,86	126	0,93
	Exp4	3716	0,67	19131	1,49	4232	2,33	6132	0,98	3202	0,94	419	1,01	99	0,66
	Exp5	3827	0,58	18474	1,52	4591	1,59	6268	0,96	3288	1,56	411	1,05	91	0,94
	**Mean**	3831	0,67	17536	1,52	4595	1,95	5762	0,97	2803	1,25	379	1,36	90	0,85
	**SD**	641	0,08	4721	0,27	485	0,29	673	0,05	719	0,29	61	0,35	24	0,23
50uM MPH	Exp1	3030	0,73	18103	1,91	4891	1,97	5714	1,03	2751	1,19	330	1,73	66	0,61
	Exp2	3308	0,55	24244	1,62	5300	1,78	6081	0,90	3034	1,49	424	1,57	71	1,13
	Exp3	4482	0,69	10578	1,27	4215	2,17	4740	1,02	1642	1,03	353	1,65	114	0,84
	Exp4	3494	0,63	19566	1,53	4364	2,40	6014	0,96	3161	0,93	430	1,04	95	0,64
	Exp5	3589	0,54	18802	1,55	4863	1,68	6335	0,97	3144	1,49	354	0,91	73	0,75
	**Mean**	3581	0,63	18259	1,58	4727	2,00	5777	0,98	2746	1,23	378	1,38	84	0,79
	**SD**	547	0,08	4921	0,23	438	0,29	621	0,05	639	0,26	46	0,38	20	0,21

		IL8		JAM_A		MCP		NOX1		OCLN		PAI_1		PECAM1	
Group	Experiments	Abosulte mRNA value	Relative value of NC	Abosulte mRNA value	Relative value of NC	Abosulte mRNA value	Relative value of NC	Abosulte mRNA value	Relative value of NC	Abosulte mRNA value	Relative value of NC	Abosulte mRNA value	Relative value of NC	Abosulte mRNA value	Relative value of NC
NC	Exp1	369	1,00	997	1,00	3433	1,00	29	1,00	99	1,00	13681	1,00	12060	1,00
	Exp2	448	1,00	1331	1,00	3636	1,00		1,00	148	1,00	13576	1,00	16439	1,00
	Exp3	849	1,00	1152	1,00	3523	1,00	31	1,00	112	1,00	19630	1,00	11863	1,00
	Exp4	555	1,00	1229	1,00	3500	1,00	31	1,00	85	1,00	17414	1,00	13716	1,00
	Exp5	369	1,00	1450	1,00	3743	1,00	46	1,00	104	1,00	13263	1,00	14226	1,00
	**Mean**	518	1,00	1232	1,00	3567	1,00	34	1,00	110	1,00	15513	1,00	13661	1,00
	**SD**	200	0,00	172	0,00	122	0,00	8	0,00	24	0,00	2861	0,00	1860	0,00
10ug/L MPH	Exp1	272	0,74	1627	1,63	3273	0,95			127	1,29	9547	0,70	17840	1,48
	Exp2	408	0,91	1819	1,37	3503	0,96			128	0,86	11442	0,84	20281	1,23
	Exp3	615	0,72	1645	1,43	2791	0,79	113	3,63	85	0,76	14806	0,75	13734	1,16
	Exp4	421	0,76	1715	1,40	2995	0,86	48	1,57	119	1,40	12402	0,71	18425	1,34
	Exp5	486	1,31	1670	1,15	2929	0,78			136	1,31	10545	0,80	18558	1,30
	**Mean**	440	0,89	1695	1,39	3098	0,87	81	2,60	119	1,12	11748	0,76	17768	1,30
	**SD**	125	0,25	77	0,17	287	0,09	46	1,45	20	0,29	2010	0,06	2431	0,12
50ug/L MPH	Exp1	251	0,68	1704	1,71	3382	0,99	19	0,66	160	1,63	9923	0,73	18563	1,54
	Exp2	380	0,85	1713	1,29	3433	0,94			174	1,17	11696	0,86	20172	1,23
	Exp3	639	0,75	1692	1,47	3023	0,86	31	0,98	108	0,97	15628	0,80	14934	1,26
	Exp4	452	0,81	1817	1,48	3241	0,93	43	1,39	143	1,68	12208	0,70	18142	1,32
	Exp5	492	1,33	1791	1,24	3027	0,81	38	0,84	115	1,11	10747	0,81	17429	1,23
	**Mean**	443	0,89	1744	1,44	3221	0,90	33	0,97	140	1,31	12040	0,78	17848	1,31
	**SD**	143	0,26	57	0,19	192	0,07	10	0,31	28	0,32	2190	0,07	1915	0,13
50uM MPH	Exp1	204	0,55	1614	1,62	3434	1,00	20	0,71	168	1,71	9132	0,67	18161	1,51
	Exp2	354	0,79	1666	1,25	3502	0,96			212	1,43	10929	0,81	21152	1,29
	Exp3	532	0,63	1688	1,47	3116	0,88	42	1,33	112	1,00	15053	0,77	15520	1,31
	Exp4	437	0,79	1607	1,31	3067	0,88			159	1,86	11739	0,67	18242	1,33
	Exp5	385	1,04	1952	1,35	3263	0,87	39	0,85	142	1,37	9651	0,73	17987	1,26
	**Mean**	382	0,76	1705	1,40	3276	0,92	33	0,96	159	1,47	11301	0,73	18212	1,34
	**SD**	121	0,19	142	0,15	191	0,06	12	0,33	37	0,33	2336	0,06	1996	0,10

		RAC1		SELE		SELP		TGFb		VCAM1		ZO1		eNOS	
Group	Experiments	Abosulte mRNA value	Relative value of NC	Abosulte mRNA value	Relative value of NC	Abosulte mRNA value	Relative value of NC	Abosulte mRNA value	Relative value of NC	Abosulte mRNA value	Relative value of NC	Abosulte mRNA value	Relative value of NC	Abosulte mRNA value	Relative value of NC
NC	Exp1	3008	1,00	29	1,00	36	1,00	6445	1,00	67	1,00	1150	1,00	737	1,00
	Exp2	3027	1,00	63	1,00	73	1,00	7327	1,00	78	1,00	1240	1,00	651	1,00
	Exp3	3291	1,00		1,00	48	1,00	6371	1,00	70	1,00	1108	1,00	781	1,00
	Exp4	3148	1,00	86	1,00	76	1,00	6402	1,00	115	1,00	1307	1,00	636	1,00
	Exp5	3271	1,00	44	1,00	43	1,00	6797	1,00	72	1,00	1292	1,00	809	1,00
	**Mean**	3149	1,00	55	1,00	55	1,00	6669	1,00	80	1,00	1219	1,00	723	1,00
	**SD**	132	0,00	25	0,00	18	0,00	406	0,00	20	0,00	87	0,00	77	0,00
10ug/L MPH	Exp1	3169	1,05					5364	0,83			1401	1,22	749	1,02
	Exp2	3279	1,08	42	0,67	105	1,44	5105	0,70	63	0,81	1526	1,23	701	1,08
	Exp3	3070	0,93					5787	0,91			990	0,89	806	1,03
	Exp4	3131	0,99	46	0,53	73	0,96	4913	0,77	58	0,51	1293	0,99	668	1,05
	Exp5	3145	0,96			66	1,53	4867	0,72	58	0,80	1427	1,10	693	0,86
	**Mean**	3159	1,01	44	0,60	81	1,31	5207	0,78	60	0,70	1327	1,09	724	1,01
	**SD**	77	0,06	3	0,10	21	0,31	378	0,09	3	0,17	206	0,15	55	0,09
50ug/L MPH	Exp1	3014	1,00	23	0,80	61	1,71	5582	0,87	52	0,77	1431	1,24	784	1,06
	Exp2	3177	1,05	61	0,97	120	1,64	5228	0,71	97	1,24	1383	1,12	686	1,05
	Exp3	3137	0,95	35	0,64	35	0,73	5870	0,92	67	0,97	1250	1,13	844	1,08
	Exp4	3235	1,03	36	0,41	86	1,13	4793	0,75	61	0,53	1545	1,18	728	1,14
	Exp5	3110	0,95	46	1,03	66	1,51	5050	0,74	62	0,85	1412	1,09	759	0,94
	**Mean**	3135	1,00	40	0,77	73	1,34	5304	0,80	68	0,87	1404	1,15	760	1,06
	**SD**	82	0,04	14	0,25	32	0,41	427	0,09	17	0,26	106	0,06	59	0,08
50uM MPH	Exp1	2977	0,99	39	1,37	49	1,37	5459	0,85	47	0,70	1498	1,30	795	1,08
	Exp2	3225	1,07	58	0,93	105	1,43	5506	0,75			1614	1,30	676	1,04
	Exp3	3084	0,94			64	1,34	6002	0,94	60	0,86	1257	1,13	786	1,01
	Exp4	3190	1,01			89	1,18	4825	0,75	44	0,38	1429	1,09	675	1,06
	Exp5	3086	0,94			60	1,38	4771	0,70	41	0,57	1450	1,12	780	0,96
	**Mean**	3113	0,99	49	1,15	73	1,34	5313	0,80	48	0,63	1450	1,19	742	1,03
	**SD**	98	0,05	14	0,31	23	0,10	516	0,10	8	0,20	129	0,10	61	0,05

Fig1E-F

Cell types	Groups	Experiments	vWF			tPA		
			OD	Conc. (ng/ml)	Relative value of NC	OD	Conc. (ng/ml)	Relative value of NC
HBEC	NC	Exp1	0,621	66,61	100%	0,385	9,55	100%
		Exp2	0,654	70,99	100%	0,380	9,40	100%
		Exp3	0,705	77,75	100%	0,388	9,64	100%
		Mean	0,660	71,785	100%	0,385	9,529	100%
		SD	0,042	5,613	0%	0,004	0,125	0%
	10ug/L MPH	Exp1	0,756	169,44	254%	1,204	32,28	338%
		Exp2	1,162	286,93	404%	0,851	23,52	250%
		Exp3	1,077	261,45	336%	1,021	27,95	290%
		Mean	0,998	239,274	332%	1,025	27,920	293%
		SD	0,214	61,805	75%	0,177	4,381	44%
	50ug/L MPH	Exp1	0,778	175,62	264%	1,136	30,74	322%
		Exp2	1,150	283,31	399%	0,824	22,80	243%
		Exp3	1,017	243,73	313%	1,025	28,07	291%
		Mean	0,981	234,220	325%	0,995	27,203	285%
		SD	0,188	54,474	69%	0,158	4,040	40%
	50uM MPH	Exp1	0,765	171,97	258%	1,150	31,05	325%
		Exp2	1,093	266,29	375%	0,831	22,98	245%
		Exp3	0,960	227,12	292%	0,988	27,13	281%
		Mean	0,939	221,795	308%	0,989	27,056	284%
		SD	0,165	47,383	60%	0,160	4,037	40%
HAEC	NC	Exp1	0,489	49,60	100%	0,391	9,73	100%
		Exp2	0,604	64,31	100%	0,149	2,56	100%
		Exp3	0,529	54,70	100%	0,191	4,27	100%
		Mean	0,540	56,203	100%	0,243	5,519	100%
		SD	0,058	7,470	0%	0,129	3,744	0%
	10ug/L MPH	Exp1	0,782	176,73	356%	1,257	33,46	344%
		Exp2	0,915	214,28	333%	0,240	6,44	251%
		Exp3	0,904	211,01	386%	0,278	8,13	190%
		Mean	0,867	200,674	358%	0,591	16,008	262%
		SD	0,074	20,798	26%	0,576	15,138	77%
	50ug/L MPH	Exp1	0,776	174,96	353%	1,253	33,38	343%
		Exp2	0,935	219,96	342%	0,284	8,42	329%
		Exp3	0,922	216,29	395%	0,304	9,35	219%
		Mean	0,878	203,740	363%	0,614	17,046	297%
		SD	0,089	24,990	28%	0,554	14,151	68%
	50uM MPH	Exp1	0,817	186,37	376%	1,284	34,05	350%
		Exp2	0,960	227,11	353%	0,309	9,58	374%
		Exp3	0,961	227,44	416%	0,293	8,81	206%
		Mean	0,912	213,641	382%	0,628	17,480	310%
		SD	0,083	23,619	32%	0,567	14,352	91%

Fig1G&FigS7B&Table S3

ID	Group1	Group2	Age_group	vWF Conc. (ng/ml)	tPA Conc. (ng/ml)	Sex	Age	BMI	BMI SDS	Methylphenidate	Lisdexamfetamine	Dexamfetamine	Atomoxetine	SNAP_Total_Mean	SNAP_Attention_mean	SNAP_Hyperactivity_mean
210	MPH	MPH ongoing	Children	12049,25	0,76	Male	12,00	21,05	1,35	Yes				1,0	1,1	0,9
248	MPH	MPH ongoing	Children	17419,94	0,86	Male	13,00	16,40	-0,98	Yes				2,6	2,9	2,3
283	MPH	MPH ongoing	Children	7237,68	0,94	Male	15,00	20,00	0,09	Yes				2,0	1,7	2,3
295	MPH	MPH ongoing	Children	11037,82	5,26	Male	14,00	20,55	0,60	Yes				0,9	0,6	1,2
348	MPH	MPH ongoing	Children	17807,77	2,62	Female	18,00	17,26	-1,59	Yes				1,4	2,3	0,4
336	MPH	MPH ongoing	Children	11131,11	1,78	Male	18,00	22,60	0,31	Yes			Yes	1,6	1,6	1,7
16	MPH	MPH ongoing	Children	9573,23	2,52	Female	9,00	NA	NA	Yes				1,3	1,6	1,0
18	MPH	MPH ongoing	Children	7724,65	0,84	Female	14,00	NA	NA	Yes				1,6	1,9	1,2
19	MPH	MPH ongoing	Children	11152,70	4,99	Female	13,00	NA	NA	Yes				0,7	0,7	0,7
21	MPH	MPH ongoing	Children	4905,35	1,07	Female	11,00	NA	NA	Yes				1,8	1,7	2,0
23	MPH	MPH ongoing	Children	3825,36	3,08	Female	11,00	NA	NA	Yes				1,4	1,4	1,3
31	MPH	MPH ongoing	Children	13935,49	1,37	Male	14,00	NA	NA	Yes				1,4	1,4	1,4
34	MPH	MPH ongoing	Children	13758,22	1,11	Male	14,00	NA	NA	Yes				0,5	0,6	0,4
22	MPH	MPH ongoing	Children	9576,24	2,52	Female	16,00	NA	NA	Yes				1,2	1,9	0,6
24	MPH	MPH ongoing	Children	13679,61	1,54	Male	13,00	NA	NA	Yes			Yes	1,5	1,8	1,2
26	MPH	MPH ongoing	Children	7793,45	1,61	Male	10,00	NA	NA	Yes			Yes	1,9	1,9	2,0
130	MPH	MPH ongoing	Children	10888,60	2,14	Male	11,00	19,90	1,26	Yes				0,7	0,8	0,7
69	MPH	MPH ongoing	Children	10261,24	1,06	Male	18,00	NA	NA	Yes				0,5	0,9	0,1
28	MPH	MPH ongoing	Children	6376,42	1,89	Male	11,00	NA	NA	Yes				2,0	2,2	1,8
158	MPH	MPH ongoing	Children	10692,96	1,34	Male	16,00	NA	NA	Yes				1,9	1,8	2,1
157	MPH	MPH ongoing	Children	10189,28	2,65	Male	10,00	NA	NA	Yes				1,9	1,9	2,0
54	MPH	MPH ongoing	Children	7625,08	0,88	Male	8,00	NA	NA	Yes				2,1	1,9	2,2
182	MPH	MPH ongoing	Children	9557,77	1,18	Male	10,00	NA	NA	Yes				1,5	1,6	1,4
52	MPH	MPH ongoing	Children	26954,13	1,23	Female	15,00	NA	NA	Yes			Yes	2,4	2,6	2,3
36	MPH	MPH ongoing	Children	5549,82	0,73	Female	8,00	NA	NA	Yes				1,7	1,7	1,7
38	MPH	MPH ongoing	Children	2409,57	2,09	Male	10,00	NA	NA	Yes				1,1	1,1	1,0
43	MPH	MPH ongoing	Children	7254,11	1,29	Female	8,00	NA	NA	Yes				2,7	2,4	3,0
46	MPH	MPH ongoing	Children	3509,18	2,28	Male	9,00	NA	NA	Yes				1,8	2,1	1,6
51	MPH	MPH ongoing	Children	14813,81	4,48	Male	9,00	NA	NA	Yes				2,2	2,4	1,9
48	MPH	MPH ongoing	Children	8739,72	2,02	Male	13,00	NA	NA	Yes				0,8	1,2	0,3
53	MPH	MPH ongoing	Children	7427,04	1,24	Male	8,00	NA	NA	Yes				1,8	1,8	1,9
37	MPH	MPH ongoing	Children	12056,32	2,21	Male	13,00	NA	NA	Yes				NA	NA	NA
202	Non-MPH	Non-MPH ongoing	Children	6876	1,75	Male	14,00	15,60	-1,91		Yes			1,1	1,6	0,6
206	Non-MPH	Non-MPH ongoing	Children	13451	1,24	Female	13,00	15,99	-1,34		Yes			0,8	1,0	0,6
281	Non-MPH	Non-MPH ongoing	Children	12862	0,59	Male	13,00	16,42	-0,96				Yes	1,5	1,4	1,6
322	Non-MPH	Non-MPH ongoing	Children	15513	1,03	Male	12,00	17,94	0,19		Yes			2,1	2,4	1,8
15	Non-MPH	Non-MPH ongoing	Children	8727	0,76	Male	12,00	NA	NA		Yes			0,8	1,1	0,4
20	Non-MPH	Non-MPH ongoing	Children	9803	1,34	Male	11,00	NA	NA		Yes			1,7	2,1	1,2
39	Non-MPH	Non-MPH ongoing	Children	9806	3,50	Male	8,00	NA	NA		Yes			0,8	0,6	0,9
30	Non-MPH	Non-MPH ongoing	Children	11212	1,80	Female	14,00	NA	NA		Yes			1,1	1,1	1,0
32	Non-MPH	Non-MPH ongoing	Children	8148	0,73	Male	8,00	NA	NA		Yes			1,8	1,9	1,8
55	Non-MPH	Non-MPH ongoing	Children	7832	0,91	Male	14,00	NA	NA		Yes			1,8	3,0	0,7
57	Non-MPH	Non-MPH ongoing	Children	8977	1,79	Female	13,00	NA	NA		Yes			2,6	2,9	2,3
100	Non-MPH	Non-MPH ongoing	Children	10198	1,89	Female	17,00	29,76	2,08		Yes			2,1	2,3	1,9
119	Non-MPH	Non-MPH ongoing	Children	4865	0,61	Male	16,00	17,30	-1,51		Yes			1,6	2,3	0,9
131	Non-MPH	Non-MPH ongoing	Children	10248	1,99	Male	12,00	16,17	-0,76			Yes		0,6	0,7	0,4
146	Non-MPH	Non-MPH ongoing	Children	11131	0,29	Male	16,00	NA	NA		Yes			0,8	1,7	0,0
136	Non-MPH	Non-MPH ongoing	Children	11094	0,93	Male	12,00	NA	NA		Yes			0,4	0,8	0,1
160	Non-MPH	Non-MPH ongoing	Children	8292	1,03	Male	13,00	NA	NA				Yes	1,1	1,2	1,0
339	Non-MPH	Non-MPH ongoing	Children	9376	1,44	Female	15,00	26,12	1,60		Yes		Yes	1,0	1,4	0,7
41	Non-MPH	Non-MPH ongoing	Children	2002	2,66	Male	13,00	NA	NA		Yes			1,4	1,6	1,2
40	Non-MPH	Non-MPH ongoing	Children	9832	3,33	Female	13,00	NA	NA		Yes			0,3	0,4	0,2
45	Non-MPH	Non-MPH ongoing	Children	3159	2,71	Male	13,00	NA	NA		Yes			1,8	2,0	1,6
47	Non-MPH	Non-MPH ongoing	Children	5357	1,92	Male	11,00	NA	NA		Yes			1,8	1,8	1,8
179	Non-MPH	Non-MPH ongoing	Children	5936	1,97	Male	14,00	NA	NA				Yes	1,3	2,2	0,3
311	Unmedicated	Drug-naïve	Children	11667,29	2,55	Male	15,00	31,75	2,73					1,4	1,9	1,0
319	Unmedicated	Drug-naïve	Children	9152,77	2,18	Female	11,00	18,09	0,36					2,0	2,2	1,8
343	Unmedicated	Drug-naïve	Children	3038,76	1,21	Female	16,00	20,32	-0,13					1,2	0,9	1,4
317	Unmedicated	Drug-naïve	Children	8163,26	1,34	Male	11,00	16,89	-0,03					1,7	2,1	1,3
350	Unmedicated	Drug-naïve	Children	7567,73	0,66	Male	9,00	15,38	-0,44					1,3	1,4	1,2
328	Unmedicated	Drug-naïve	Children	8932,03	1,48	Male	13,00	16,00	-1,23					1,4	1,1	1,6
354	Unmedicated	Drug-naïve	Children	8613,89	0,95	Male	10,00	18,35	0,95					2,1	1,9	2,2
17	Unmedicated	Drug-naïve	Children	3719,05	5,19	Female	11,00	NA	NA					1,7	2,7	0,8
42	Unmedicated	Drug-naïve	Children	7630,29	1,14	Male	15,00	NA	NA					2,7	2,9	2,6
59	Unmedicated	Drug-naïve	Children	4879,64	1,03	Male	9,00	NA	NA					1,6	2,2	1,0
118	Unmedicated	Drug-naïve	Children	6399,07	1,30	Male	13,00	17,51	-0,35					1,8	2,0	1,7
50	Unmedicated	Drug-naïve	Children	7701,75	1,63	Male	15,00	NA	NA					1,6	2,0	1,1
66	Unmedicated	Drug-naïve	Children	11040,48	2,24	Male	14,00	NA	NA					1,8	1,8	1,9
80	Unmedicated	Drug-naïve	Children	4687,87	3,86	Female	18,00	18,78	-0,91					1,6	2,0	1,1
123	Unmedicated	Drug-naïve	Children	7487,85	1,65	Female	16,00	NA	NA					1,2	2,0	0,6
221	Unmedicated	Drug-naïve	Children	8723,85	1,45	Female	15,00	23,18	0,91					1,3	1,9	0,8
333	Unmedicated	Drug-naïve	Children	8528,79	1,13	Male	11,00	19,71	1,20					2,4	2,4	2,3
301	Unmedicated	Drug-naïve	Children	7498,70	2,23	Male	10,00	16,04	-0,24					2,1	2,4	1,8
306	Unmedicated	Drug-naïve	Children	10509,41	1,62	Male	12,00	18,66	0,51					2,1	1,4	2,7
310	Unmedicated	Drug-naïve	Children	12078,13	0,80	Male	10,00	15,95	-0,30					1,7	1,9	1,6
330	Unmedicated	Drug-naïve	Children	7821,88	1,24	Male	12,00	16,00	-0,86					2,5	2,4	2,6
312	Unmedicated	Drug-naïve	Children	12602,65	1,18	Male	12,00	19,91	0,99					1,6	1,4	1,7
159	Unmedicated	Drug-naïve	Children	10189,28	1,18	Male	10,00	NA	NA					1,6	1,9	1,3
121	Unmedicated	Drug-naïve	Children	3560,78	1,26	Female	11,00	NA	NA					2,2	2,2	2,2
204	Unmedicated	Drug-naïve	Children	7749,99	1,30	Male	13,00	58,01	4,23					1,8	1,4	2,2
99	Unmedicated	Drug-naïve	Children	7764,15	1,08	Female	16,00	29,76	2,15					1,9	2,7	1,1
137	Unmedicated	Drug-naïve	Children	1819,15	2,19	Female	17,00	19,33	-0,62					0,6	1,2	0,0
138	Unmedicated	Drug-naïve	Children	6089,90	1,41	Female	14,00	NA	NA					1,0	1,8	0,2
145	Unmedicated	Drug-naïve	Children	7469,04	1,42	Male	8,00	NA	NA					2,3	2,3	2,3
213	Unmedicated	Drug-naïve	Children	9629,64	1,46	Male	8,00	13,19	-2,11					2,3	2,2	2,3
261	Unmedicated	Drug-naïve	Children	5340,23	0,98	Male	12,00	20,00	1,02					1,3	1,6	1,1
279	Unmedicated	Drug-naïve	Children	5876,13	1,41	Female	17,00	20,32	-0,25					1,6	1,9	1,2
307	Control	Control	Children	4844,53	1,41	Male	13,00	16,80	-0,74					0,3	0,4	0,2
56	Control	Control	Children	4480,32	0,90	Male	14,00	NA	NA					0,1	0,1	0,0
302	Control	Control	Children	6502,54	0,84	Male	12,00	15,01	-1,55					0,3	0,7	0,0
122	Control	Control	Children	3621,60	1,52	Female	13,00	NA	NA					0,6	0,8	0,3

Fig1H&FigS7A&Table S4

ID	Group1	Group2	Age_group	vWF Conc. (ng/ml)	Sex	Age	BMI	Methylphenidate	Lisdexamfetamine	Dexamfetamine	Atomoxetine	Antidepression	Antipsychotic	Anxiolytics	Statins	Concerta	Equasym	Medikinet	Ritalin	Elvanse	Attentin	Metamina	Strattera	ASRS_Total_mean	ASRS_A_mean	ASRS_B_mean
77	MPH	MPH ongoing	Adult	10873	Male	35	23,8	Yes				0	0	0	0	2	0	0	2	0	0	0	0	2,61	3,22	2,00
81	MPH	Taken MPH in the last 3 months	Adult	21002	Male	26	27,5	Yes				0	0	0	0	3	0	1	3	1	0	0	0	2,11	1,89	2,33
95	MPH	Taken MPH in the last 3 months	Adult	18553	Female	43	25,3	Yes				2	0	0	0	3	0	0	1	0	0	0	0	2,72	3,78	1,67
98	MPH	MPH ongoing	Adult	14198	Female	30	27,0	Yes				0	0	0	0	2	0	0	3	0	0	0	0	2,33	2,44	2,22
103	MPH	MPH ongoing	Adult	22328	Female	43	25,7	Yes				2	0	0	0	0	0	0	2	0	0	0	0	2,00	1,78	2,22
115	MPH	MPH ongoing	Adult	7335	Female	46	23,9	Yes				0	0	0	0	2	0	0	0	0	0	0	0	1,56	1,67	1,44
117	MPH	Taken MPH in the last 3 months	Adult	6591	Male	30	22,4	Yes				0	0	0	0	3	0	0	0	0	0	0	0	2,00	2,67	1,33
126	MPH	MPH ongoing	Adult	12791	Female	28	20,2	Yes				0	0	3	0	0	0	0	2	0	0	0	0	2,56	3,00	2,11
128	MPH	MPH ongoing	Adult	11574	Male	42	26,6	Yes				2	0	0	0	2	0	0	0	1	0	0	0	1,44	2,11	0,78
134	MPH	Taken MPH in the last 3 months	Adult	13149	Female	37	26,6	Yes				0	0	1	0	0	3	0	0	0	0	0	0	2,44	3,00	1,89
144	MPH	MPH ongoing	Adult	2168	Male	40	28,4	Yes				0	0	0	0	2	0	0	0	0	0	0	0	2,94	3,44	2,44
150	MPH	MPH ongoing	Adult	16093	Female	32	31,9	Yes				0	0	0	0	0	0	3	2	0	0	0	0	2,56	2,56	2,56
152	MPH	MPH ongoing	Adult	14801	Female	38	23,1	Yes				0	0	0	0	2	0	2	3	1	1	0	0	2,17	2,22	2,11
155	MPH	MPH ongoing	Adult	7842	Female	19	25,7	Yes				0	0	0	0	0	0	0	2	0	0	0	0	2,89	3,22	2,56
156	MPH	MPH ongoing	Adult	5636	Female	43	34,3	Yes				2	0	0	0	2	0	0	0	1	0	0	0	2,56	3,00	2,11
169	MPH	MPH ongoing	Adult	3930	Male	41	24,4	Yes				0	0	0	0	2	0	0	3	0	0	0	0	3,17	3,33	3,00
174	MPH	MPH ongoing	Adult	14541	Female	42	18,5	Yes				0	0	0	0	0	0	2	0	0	0	0	0	2,67	3,00	2,33
180	MPH	MPH ongoing	Adult	7262	Female	42	27,1	Yes				0	0	0	0	2	0	0	0	1	0	0	0	2,11	2,89	1,33
209	MPH	MPH ongoing	Adult	4078	Female	50	25,7	Yes				0	0	0	0	2	0	0	0	0	0	0	0	3,39	3,44	3,33
215	MPH	MPH ongoing	Adult	10652	Male	41	22,2	Yes				0	0	0	0	2	0	0	0	0	0	0	0	2,83	3,00	2,67
222	MPH	MPH ongoing	Adult	16404	Male	29	21,2	Yes				0	0	0	0	2	0	0	0	0	0	0	0	1,67	1,78	1,56
223	MPH	MPH ongoing	Adult	5312	Female	18	23,0	Yes				2	0	0	0	0	0	2	2	0	0	0	0	2,06	2,78	1,33
234	MPH	MPH ongoing	Adult	11111	Male	27	22,7	Yes				0	0	0	0	2	0	0	0	0	0	0	0	2,44	2,78	2,11
243	MPH	MPH ongoing	Adult	3488	Female	40	23,0	Yes				2	0	0	0	2	0	0	0	0	0	0	0	2,06	2,33	1,78
249	MPH	Taken MPH in the last 3 months	Adult	3141	Female	25	21,2	Yes				0	0	0	0	1	0	0	3	0	0	0	0	3,11	3,22	3,00
260	MPH	MPH ongoing	Adult	10233	Female	40	21,5	Yes				0	0	0	0	0	0	0	2	0	0	0	0	2,94	3,44	2,44
270	MPH	MPH ongoing	Adult	7439	Male	50	25,8	Yes				2	0	0	0	0	0	0	2	0	0	0	0	2,56	2,67	2,44
292	MPH	MPH ongoing	Adult	5566	Male	42	22,2	Yes				0	0	0	0	2	1	0	2	0	0	0	0	1,44	2,22	0,67
299	MPH	MPH ongoing	Adult	4734	Female	25	27,8	Yes				0	0	0	0	2	0	0	0	0	0	0	0	1,94	2,33	1,56
304	MPH	MPH ongoing	Adult	6724	Female	29	29,3	Yes				0	0	0	0	2	0	0	0	0	0	0	0	2,11	2,67	1,56
313	MPH	MPH ongoing	Adult	12293	Female	23	45,9	Yes				2	0	1	0	2	0	0	0	0	0	0	0	1,67	2,56	0,78
318	MPH	MPH ongoing	Adult	6804	Female	22	22,6	Yes				0	0	0	0	2	0	0	0	0	0	0	0	2,17	2,11	2,22
345	MPH	MPH ongoing	Adult	2367	Male	41	27,7	Yes				2	0	0	0	2	0	0	0	0	0	0	0	2,56	3,11	2,00
124	MPH	MPH ongoing	Adult	10031	Male	37	25,4	Yes	Yes			0	3	0	0	2	0	0	0	3	0	0	0	1,50	1,89	1,11
154	MPH	Taken MPH in the last 3 months	Adult	8574	Male	33	27,5	Yes	Yes			2	0	1	0	3	0	0	3	2	0	0	0	2,61	2,67	2,56
161	MPH	Taken MPH in the last 3 months	Adult	12333	Female	43	22,5	Yes			Yes	0	0	0	0	0	0	0	3	0	0	0	3	3,44	3,78	3,11
256	MPH	Taken MPH in the last 3 months	Adult	3859	Male	43	22,0	Yes	Yes			0	0	0	0	3	0	0	3	2	0	0	0	2,28	2,78	1,78
73	Non-MPH	Taken non-MPH in the last 3 month	Adult	8151	Male	31	25,3		Yes			1	0	3	0	1	0	0	0	3	0	0	1	2,44	2,44	2,44
76	Non-MPH	Non-MPH ongoing	Adult	6436	Male	35	26,6		Yes			0	0	0	0	0	0	0	0	2	0	0	0	1,50	2,44	0,56
83	Non-MPH	Non-MPH ongoing	Adult	9051	Male	37	28,7		Yes	Yes		0	0	0	0	0	0	0	0	2	2	1	0	1,56	1,89	1,22
86	Non-MPH	Non-MPH ongoing	Adult	6061	Male	40	26,9		Yes			0	0	0	0	0	0	0	0	2	0	1	0	2,28	2,22	2,33
90	Non-MPH	Non-MPH ongoing	Adult	3834	Male	21	23,4		Yes			2	1	3	0	1	0	0	1	2	0	0	0	2,17	2,44	1,89
106	Non-MPH	Non-MPH ongoing	Adult	10853	Male	28	22,3		Yes			0	0	0	0	0	0	0	0	2	0	0	0	2,67	2,11	3,22
109	Non-MPH	Non-MPH ongoing	Adult	6983	Male	37	22,3			Yes		1	0	0	0	0	0	0	0	1	2	0	0	2,17	3,00	1,33
112	Non-MPH	Non-MPH ongoing	Adult	10505	Female	30	24,2		Yes			2	0	0	0	0	0	0	1	2	0	0	0	2,61	2,78	2,44
129	Non-MPH	Non-MPH ongoing	Adult	13290	Male	39	28,3		Yes			0	0	0	0	1	0	0	0	2	0	0	1	2,39	2,56	2,22
139	Non-MPH	Non-MPH ongoing	Adult	9187	Male	32	24,2			Yes		1	0	0	0	0	0	0	0	1	2	3	0	2,28	2,67	1,89
142	Non-MPH	Non-MPH ongoing	Adult	11538	Female	34	24,1		Yes	Yes		2	0	0	0	0	0	0	0	2	2	0	0	2,50	2,44	2,56
147	Non-MPH	Non-MPH ongoing	Adult	5473	Female	30	27,9		Yes			0	0	0	0	1	0	0	0	2	0	0	0	2,72	2,67	2,78
153	Non-MPH	Non-MPH ongoing	Adult	14611	Male	25	23,1			Yes		0	0	0	0	1	0	0	0	1	2	0	0	2,28	3,33	1,22
167	Non-MPH	Taken non-MPH in the last 3 month	Adult	11958	Male	33	26,5		Yes	Yes		0	0	0	0	1	0	1	1	3	3	0	0	1,28	1,78	0,78
216	Non-MPH	Non-MPH ongoing	Adult	2352	Female	28	21,5		Yes			0	0	0	0	0	0	0	0	2	0	0	0	1,89	1,78	2,00
217	Non-MPH	Non-MPH ongoing	Adult	2097	Male	45	22,8		Yes			0	2	0	0	0	0	0	0	2	0	0	0	2,29	2,75	1,89
229	Non-MPH	Non-MPH ongoing	Adult	2180	Male	25	20,3		Yes			0	0	0	0	1	0	0	0	2	0	0	0	1,56	2,11	1,00
240	Non-MPH	Non-MPH ongoing	Adult	1740	Female	28	32,0		Yes			2	0	2	0	0	0	0	0	2	0	0	0	2,61	2,56	2,67
259	Non-MPH	Non-MPH ongoing	Adult	8041	Male	40	22,8		Yes			0	0	0	0	0	0	1	0	2	1	0	0	2,39	3,22	1,56
291	Non-MPH	Non-MPH ongoing	Adult	5820	Female	24	19,5		Yes	Yes		2	0	0	0	0	0	0	1	2	2	0	0	2,72	2,67	2,78
332	Non-MPH	Non-MPH ongoing	Adult	2233	Female	27	22,9		Yes			0	0	0	0	1	0	0	1	2	0	0	0	2,22	3,33	1,11
342	Non-MPH	Non-MPH ongoing	Adult	2440	Female	26	34,7		Yes	Yes		3	0	3	0	0	0	0	1	2	3	0	0	2,00	2,56	1,44
346	Non-MPH	Non-MPH ongoing	Adult	6778	Male	44	25,1		Yes			0	2	0	0	0	0	0	1	2	0	0	0	3,50	3,89	3,11
71	Unmedicated	No drug intake last 2 years	Adult	22947	Male	45	27,2					2	0	2	2	0	0	0	0	0	0	0	0	2,83	2,33	3,33
85	Unmedicated	Taken in last 2 years	Adult	6187	Female	40	25,3					2	0	0	0	0	0	1	1	1	1	0	0	3,17	3,22	3,11
82	Unmedicated	Taken in last 2 years	Adult	8767	Female	26	30,4					1	0	1	0	1	0	0	0	0	0	0	0	2,59	3,50	1,78
84	Unmedicated	No drug intake last 2 years	Adult	12540	Male	45	23,4					0	0	0	0	0	0	0	0	0	0	0	0	2,89	2,89	2,89
92	Unmedicated	No drug intake last 2 years	Adult	5199	Female	40	23,7					2	0	0	0	0	0	0	0	0	0	0	0	3,11	3,33	2,89
93	Unmedicated	Taken in last 2 years	Adult	10401	Female	34	25,8					0	0	0	0	0	0	0	1	0	0	0	0	1,83	2,22	1,44
94	Unmedicated	No drug intake last 2 years	Adult	5134	Female	39	28,4					0	0	0	0	0	0	0	0	0	0	0	0	1,78	1,89	1,67
96	Unmedicated	No drug intake last 2 years	Adult	10340	Female	33	19,3					0	0	0	0	0	0	0	0	0	0	0	0	3,17	3,22	3,11
97	Unmedicated	No drug intake last 2 years	Adult	7562	Female	35	21,1					2	0	0	0	0	0	0	0	0	0	0	0	1,83	1,89	1,78
101	Unmedicated	No drug intake last 2 years	Adult	7307	Female	38	23,3					3	0	0	0	0	0	0	0	0	0	0	0	2,28	2,33	2,22
104	Unmedicated	Taken in last 2 years	Adult	4442	Female	38	24,9					0	0	0	0	0	0	0	0	1	0	0	0	2,83	2,78	2,89
102	Unmedicated	Taken in last 2 years	Adult	15305	Female	29	22,5					3	0	0	0	0	0	1	0	0	0	0	0	2,06	2,00	2,11
105	Unmedicated	Taken in last 2 years	Adult	9235	Female	42	26,7					0	0	0	0	0	0	0	0	1	1	0	0	2,17	2,44	1,89
111	Unmedicated	No drug intake last 2 years	Adult	7948	Male	34	28,1					0	0	1	0	0	0	0	0	0	0	0	0	2,06	2,22	1,89
113	Unmedicated	Taken in last 2 years	Adult	12662	Female	33	33,1					0	0	0	0	0	0	0	0	1	0	0	0	3,56	3,67	3,44
114	Unmedicated	No drug intake last 2 years	Adult	13293	Female	42	22,2					3	0	0	0	0	0	0	0	0	0	0	0	2,50	3,56	1,44
116	Unmedicated	No drug intake last 2 years	Adult	5308	Female	40	20,4					1	0	0	0	0	0	0	0	0	0	0	0	1,83	2,11	1,56
125	Unmedicated	No drug intake last 2 years	Adult	4786	Female	35	25,1					0	0	0	0	0	0	0	0	0	0	0	0	2,83	3,44	2,22
149	Unmedicated	Taken in last 2 years	Adult	13291	Male	43	22,7					0	0	0	0	0	0	0	0	1	0	0	0	2,72	2,11	3,33
151	Unmedicated	No drug intake last 2 years	Adult	10577	Male	33	27,4					0	0	0	0	0	0	0	0	0	0	0	0	2,39	2,56	2,22
164	Unmedicated	Taken in last 2 years	Adult	12693	Female	24	23,7					1	0	0	0	1	0	0	1	1	0	0	0	2,78	2,78	2,78
165	Unmedicated	Taken in last 2 years	Adult	6189	Female	23	21,8					0	0	0	0	0	0	0	1	0	0	0	0	3,11	3,56	2,67
172	Unmedicated	No drug intake last 2 years	Adult	10510	Female	41	29,4					0	0	0	0	0	0	0	0	0	0	0	0	2,44	2,44	2,44
176	Unmedicated	Taken in last 2 years	Adult	4655	Female	37	22,6					0	0	3	0	1	0	0	1	1	1	0	1	2,00	2,56	1,44
191	Unmedicated	No drug intake last 2 years	Adult	14369	Female	26	24,8					0	0	0	0	0	0	0	0	0	0	0	0	2,28	2,33	2,22
208	Unmedicated	No drug intake last 2 years	Adult	7211	Female	24	34,9					2	0	0	0	0	0	0	0	0	0	0	0	3,11	3,22	3,00
212	Unmedicated	No drug intake last 2 years	Adult	3473	Female	43	23,9					2	0	0	0	0	0	0	0	0	0	0	0	2,89	3,56	2,22
219	Unmedicated	No drug intake last 2 years	Adult	7559	Female	36	29,7					0	0	0	0	0	0	0	0	0	0	0	0	1,78	2,78	0,78
227	Unmedicated	No drug intake last 2 years	Adult	7686	Male	26	25,7					0	0	0	0	0	0	0	0	0	0	0	0	0,56	0,89	0,22
228	Unmedicated	No drug intake last 2 years	Adult	2576	Female	46	26,1					0	0	0	0	0	0	0	0	0	0	0	0	3,56	3,78	3,33
231	Unmedicated	No drug intake last 2 years	Adult	6941	Female	40	20,5					2	0	0	0	0	0	0	0	0	0	0	0	3,22	3,33	3,11
238	Unmedicated	No drug intake last 2 years	Adult	2613	Female	34	38,3					0	0	0	0	0	0	0	0	0	0	0	0	2,83	3,22	2,44
255	Unmedicated	Taken in last 2 years	Adult	5606	Female	30	22,5					0	0	0	0	1	0	0	0	0	0	0	0	2,06	2,56	1,56
258	Unmedicated	No drug intake last 2 years	Adult	9363	Female	24	23,7					0	0	0	0	0	0	0	0	0	0	0	0	2,33	2,33	2,33
272	Unmedicated	No drug intake last 2 years	Adult	8381	Female	47	20,4					0	0	0	0	0	0	0	0	0	0	0	0	3,61	3,22	4,00
277	Unmedicated	Taken in last 2 years	Adult	3253	Male	20	17,0					0	0	0	0	1	0	0	0	0	0	0	0	0,50	0,78	0,22
278	Unmedicated	Taken in last 2 years	Adult	6006	Male	20	18,2					0	0	0	0	1	0	0	0	0	0	0	0	1,72	2,11	1,33
285	Unmedicated	Taken in last 2 years	Adult	6980	Male	33	22,9					0	0	0	0	0	0	0	0	0	1	0	0	1,72	1,89	1,56
294	Unmedicated	Taken in last 2 years	Adult	820	Female	42	30,1					0	0	0	0	1	0	0	0	0	0	0	0	2,83	3,22	2,44
305	Unmedicated	No drug intake last 2 years	Adult	8470	Female	31	18,3					0	0	0	0	0	0	0	0	0	0	0	0	2,83	3,22	2,44
324	Unmedicated	No drug intake last 2 years	Adult	9667	Female	33	28,6					2	0	2	0	0	0	0	0	0	0	0	0	3,00	3,33	2,67
325	Unmedicated	No drug intake last 2 years	Adult	6604	Female	51	21,8					2	0	3	0	0	0	0	0	0	0	0	0	2,89	3,56	2,22
88	Control		Adult	3157	Male	37	28,4					0	0	0	0	0	0	0	0	0	0	0	0	1,50	1,78	1,22
110	Control		Adult	9400	Female	36	22,9					0	0	3	0	0	0	0	0	0	0	0	0	1,06	1,33	0,78
133	Control		Adult	12376	Male	23	23,6					0	0	0	0	0	0	0	0	0	0	0	0	2,11	2,33	1,89
143	Control		Adult	9845	Male	35	27,2					0	0	0	0	0	0	0	0	0	0	0	0	1,44	1,44	1,44
162	Control		Adult	8805	Male	35	26,7					0	0	0	0	0	0	0	0	0	0	0	0	1,00	0,78	1,22
166	Control		Adult	8489	Male	25	30,5					0	0	0	0	0	0	0	0	0	0	0	0	1,11	1,22	1,00
168	Control		Adult	3992	Male	27	23,2					0	0	0	0	0	0	0	0	0	0	0	0	1,72	1,11	2,33
173	Control		Adult	5051	Male	37	21,3					0	0	0	0	0	0	0	0	0	0	0	0	2,28	2,78	1,78
175	Control		Adult	10455	Female	31	25,9					0	0	0	0	0	0	0	0	0	0	0	0	0,83	0,89	0,78
177	Control		Adult	11736	Female	30	24,6					0	0	0	0	0	0	0	0	0	0	0	0	0,61	0,56	0,67
178	Control		Adult	15986	Male	33	26,1					0	0	0	0	0	0	0	0	0	0	0	0	1,22	0,78	1,67
181	Control		Adult	7280	Female	42	25,2					0	0	0	0	0	0	0	0	0	0	0	0	0,67	1,00	0,33
187	Control		Adult	14573	Female	36	22,0					0	0	0	0	0	0	0	0	0	0	0	0	1,06	1,00	1,11
193	Control		Adult	9694	Female	41	22,7					0	0	0	0	0	0	0	0	0	0	0	0	0,78	1,11	0,44
194	Control		Adult	15738	Female	38	22,2					0	0	0	0	0	0	0	0	0	0	0	0	0,17	0,22	0,11
195	Control		Adult	10816	Female	47	23,9					0	0	0	0	0	0	0	0	0	0	0	0	1,72	2,11	1,33
196	Control		Adult	16182	Female	30	19,7					0	0	0	0	0	0	0	0	0	0	0	0	1,33	1,22	1,44
197	Control		Adult	5507	Female	35	20,6					0	0	0	0	0	0	0	0	0	0	0	0	1,61	1,56	1,67
198	Control		Adult	9615	Female	32	20,5					0	0	0	0	0	0	0	0	0	0	0	0	1,22	1,33	1,11
207	Control		Adult	3671	Male	34	24,0					0	0	0	0	0	0	0	0	0	0	0	0	1,94	1,89	2,00
211	Control		Adult	7163	Female	36	21,3					0	0	0	0	0	0	0	0	0	0	0	0	1,50	0,89	2,11
214	Control		Adult	12422	Male	45	22,6					0	0	0	0	0	0	0	0	0	0	0	0	1,44	2,33	0,56
218	Control		Adult	2626	Female	42	23,4					0	0	0	0	0	0	0	0	0	0	0	0	1,50	2,00	1,00
230	Control		Adult	5759	Female	46	21,4					0	0	0	0	0	0	0	0	0	0	0	0	1,78	1,56	2,00
232	Control		Adult	10070	Male	46	20,8					0	0	0	0	0	0	0	0	0	0	0	0	1,00	1,22	0,78
235	Control		Adult	3371	Female	27	19,0					0	0	0	0	0	0	0	0	0	0	0	0	1,28	1,33	1,22
237	Control		Adult	1328	Male	44	33,2					0	0	0	0	0	0	0	0	0	0	0	0	0,94	1,00	0,89
250	Control		Adult	4081	Male	25	23,0					0	0	0	0	0	0	0	0	0	0	0	0	#N/A	#N/A	#N/A
263	Control		Adult	2373	Female	47	23,0					0	0	0	0	0	0	0	0	0	0	0	0	1,28	1,22	1,33
276	Control		Adult	15727	Female	49	25,6					0	0	0	0	0	0	0	0	0	0	0	0	#N/A	#N/A	#N/A
282	Control		Adult	2711	Male	43	22,2					0	0	0	0	0	0	0	0	0	0	0	0	2,06	2,22	1,89
284	Control		Adult	4217	Female	42	21,5					0	0	0	0	0	0	0	0	0	0	0	0	1,56	1,11	2,00
287	Control		Adult	2752	Female	44	23,4					0	0	0	0	0	0	0	0	0	0	0	0	1,00	0,67	1,33
300	Control		Adult	14813	Female	43	21,7					0	0	0	0	0	0	0	0	0	0	0	0	0,89	1,11	0,67
303	Control		Adult	9454	Male	43	25,6					0	0	0	0	0	0	0	0	0	0	0	0	1,94	2,11	1,78
308	Control		Adult	18710	Female	43	37,6					0	0	0	0	0	0	0	0	0	0	0	0	1,33	1,33	1,33
309	Control		Adult	6064	Male	43	29,9					0	0	0	0	0	0	0	0	0	0	0	0	0,94	1,33	0,56
327	Control		Adult	3452	Male	41	30,7					0	0	0	0	0	0	0	0	0	0	0	0	0,33	0,44	0,22
329	Control		Adult	12719	Female	43	21,3					0	0	0	0	0	0	0	0	0	0	0	0	1,39	2,00	0,78
331	Control		Adult	2898	Female	38	34,3					0	0	0	0	0	0	0	0	0	0	0	0	0,94	1,44	0,44
334	Control		Adult	9625	Female	32	24,0					0	0	0	0	0	0	0	0	0	0	0	0	0,72	0,78	0,67
335	Control		Adult	2974	Female	44	23,6					0	0	0	0	0	0	0	0	0	0	0	0	1,61	1,78	1,44
351	Control		Adult	5134	Female	35	35,3					0	0	0	0	0	0	0	0	0	0	0	0	0,78	1,00	0,56
341	Control		Adult	6617	Male	42	23,7					0	0	0	0	0	0	0	0	0	0	0	0	0,94	1,33	0,56

3:Used last 3 months,
2:Ongoing treatment,
1:Sometime the last 2 years,
0/NA:no drug

Fig2B

Cell types	Groups	Experiments	CLDN5	bactin	CLDN5 (Norm by bactin)	CLDN5 (Relative value of NC)
HBEC	NC	Exp1	16035	12254	1,31	1,00
		Exp2	21151	26939	0,79	1,00
		Exp3	30195	26426	1,14	1,00
	Mean				1,08	1,00
	SD				0,27	0,00
	10µg/L	Exp1	31290	23077	1,36	1,04
		Exp2	24138	25792	0,94	1,19
		Exp3	31133	26539	1,17	1,03
	Mean				1,15	1,08
	SD				0,21	0,09
	50µg/L	Exp1	20699	32231	0,64	0,49
		Exp2	15482	32142	0,48	0,61
		Exp3	28702	26208	1,10	0,96
	Mean				0,74	0,69
	SD				0,32	0,24
	50µM	Exp1	18265	26712	0,68	0,52
		Exp2	13826	31670	0,44	0,56
		Exp3	28346	27769	1,02	0,89
	Mean				0,71	0,66
	SD				0,29	0,21
	100µM	Exp1	5930	29710	0,20	0,15
		Exp2	15326	28317	0,54	0,69
		Exp3	15498	27463	0,56	0,49
	Mean				0,44	0,45
	SD				0,20	0,27
HAEC	NC	Exp1	25041	26649	0,94	1,00
		Exp2	27408	21399	1,28	1,00
		Exp3	26209	15910	1,65	1,00
	Mean				1,29	1,00
	SD				0,35	0,00
	10µg/L	Exp1	29285	30515	0,96	1,02
		Exp2	31692	26241	1,21	0,94
		Exp3	30000	28454	1,05	0,64
	Mean				1,07	0,87
	SD				0,13	0,20
	50µg/L	Exp1	24356	32806	0,74	0,79
		Exp2	30347	24537	1,24	0,97
		Exp3	21742	21388	1,02	0,62
	Mean				1,00	0,79
	SD				0,25	0,17
	50µM	Exp1	25651	30943	0,83	0,88
		Exp2	29483	26525	1,11	0,87
		Exp3	19323	22204	0,87	0,53
	Mean				0,94	0,76
	SD				0,15	0,20
	100µM	Exp1	24041	31957	0,75	0,80
		Exp2	21469	32535	0,66	0,52
		Exp3	24230	32101	0,75	0,46
	Mean				0,72	0,59
	SD				0,05	0,18

Fig2D

Cell types	Groups	Experiments	Cell number	DAPI total intensity	Claudin5 total intensity	Claudin5 total intensity / cell number	Claudin5 Relative value of NC
HBEC	NC	Exp1	84	36706130	30833848	367070	1,00
		Exp2	80	37458710	14100673	176258	1,00
		Exp3	83	39643549	17263726	207997	1,00
		Exp4	84	34551621	23163681	275758	1,00
	Mean		83	37090003	21340482	256771	1,00
	SD		2	2101371	7359412	84434	0,00
	10ug/L MPH	Exp1	87	43802898	26974735	310054	0,84
		Exp2	92	41907589	21045799	228759	1,30
		Exp3	93	38910050	21646910	232762	1,12
		Exp4	97	46410648	25112453	258891	0,94
	Mean		92	42757796	23694974	257617	1,05
	SD		4	3160357	2827176	37425	0,20
	50ug/L MPH	Exp1	84	40901659	24089062	286775	0,78
		Exp2	93	41330509	17770696	191083	1,08
		Exp3	93	43445688	18335087	197151	0,95
		Exp4	75	38062700	15475244	206337	0,75
	Mean		86	40935139	18917522	220336	0,89
	SD		9	2214438	3662811	44734	0,16
	50uM MPH	Exp1	96	45580938	19858338	206858	0,56
		Exp2	89	42797419	9175868	103100	0,58
		Exp3	79	39013730	10456570	132362	0,64
		Exp4	84	39872419	12280331	146194	0,53
	Mean		87	41816127	12942777	147128	0,58
	SD		7	2987093	4783118	43685	0,04
	100uM MPH	Exp1	79	39693589	14823194	187635	0,51
		Exp2	94	44212848	5579555	59357	0,34
		Exp3	72	36024110	8377208	116350	0,56
		Exp4	91	41530489	14184103	155869	0,57
	Mean		84	40365259	10741015	129803	0,49
	SD		10	3437946	4499900	55280	0,11
HAEC	NC	Exp1	158	12956594	6793694	42998	1,00
		Exp2	175	12041190	11022579	62986	1,00
		Exp3	157	12431255	8766579	55838	1,00
	Mean		163	12476346	8860951	53941	1,00
	SD		10	459365	2116021	10128	0,00
	10ug/L MPH	Exp1	145	10835186	2485492	17141	0,40
		Exp2	155	12541452	10756529	69397	1,10
		Exp3	147	10592799	6763060	46007	0,82
	Mean		149	11323146	6668360	44182	0,77
	SD		5	1062022	4136332	26176	0,35
	50ug/L MPH	Exp1	177	13980415	5787081	32695	0,76
		Exp2	160	12401698	8713150	54457	0,86
		Exp3	150	10277857	4960850	33072	0,59
	Mean		162	12219990	6487027	40075	0,74
	SD		14	1857955	1971645	12457	0,14
	50uM MPH	Exp1	163	10770314	1970961	12092	0,28
		Exp2	157	12004605	4958459	31583	0,50
		Exp3	163	10589694	2319525	14230	0,25
	Mean		161	11121538	3082982	19302	0,35
	SD		3	770073	1633535	10689	0,14
	100uM MPH	Exp1	157	10697337	1179434	7512	0,17
		Exp2	158	12594194	3532002	22354	0,35
		Exp3	158	12149740	1993465	12617	0,23
	Mean		158	11813757	2234967	14161	0,25
	SD		1	992058	1194733	7541	0,09

Fig2G-H

Cell types	Groups	Experiments	Live cells (%)	CLDN5$^+$ cells(%)	CLDN5+ cells (Relative value of NC)	Medium FI	Medium FI (Relative value of NC)
HBEC	NC	Exp1	95,60	81,15	1,00	2346,00	1,00
		Exp2	97,20	58,00	1,00	1369,00	1,00
		Exp3	95,30	92,10	1,00	3800,00	1,00
	Mean			77,08	1,00	2505,00	1,00
	SD			17,41	0,00	1223,27	0,00
	10μg/L	Exp1	96,60	76,97	0,95	2095,00	0,89
		Exp2	98,20	44,30	0,76	1035,00	0,76
		Exp3	93,10	92,53	1,00	3942,00	1,04
	Mean			71,27	0,91	2357,33	0,90
	SD			24,62	0,13	1471,15	0,14
	50μg/L	Exp1	93,80	76,03	0,94	2048,00	0,87
		Exp2	99,10	51,90	0,89	1220,00	0,89
		Exp3	92,10	86,28	0,94	3197,00	0,84
	Mean			71,40	0,92	2155,00	0,87
	SD			17,65	0,02	992,83	0,03
	50μM	Exp1	91,30	73,38	0,90	1950,00	0,83
		Exp2	97,30	50,30	0,87	1180,00	0,86
		Exp3	94,20	66,04	0,72	1434,00	0,38
	Mean			63,24	0,83	1521,33	0,69
	SD			11,79	0,10	392,36	0,27
	100μM	Exp1	95,90	70,88	0,87	1639,00	0,70
		Exp2	97,80	45,30	0,78	1019,00	0,74
		Exp3	95,90	64,60	0,70	1463,00	0,39
	Mean			60,26	0,79	1373,67	0,61
	SD			13,33	0,09	319,51	0,20
HAEC	NC	Exp1	98,40	73,90	1,00	1928,00	1,00
		Exp2	95,60	98,00	1,00	3761,00	1,00
		Exp3	94,30	64,60	1,00	1366,00	1,00
	Mean			78,83	1,00	2351,67	1,00
	SD			17,24	0,00	1252,45	0,00
	10μg/L	Exp1	99,40	72,80	0,99	1950,00	1,01
		Exp2	97,40	96,00	0,98	3863,00	1,03
		Exp3	96,20	56,90	0,88	1056,00	0,77
	Mean			75,23	0,95	2289,67	0,94
	SD			19,66	0,06	1434,00	0,14
	50μg/L	Exp1	98,70	69,30	0,94	1588,00	0,82
		Exp2	96,90	91,10	0,93	2270,00	0,60
		Exp3	98,40	52,80	0,82	929,00	0,68
	Mean			71,07	0,89	1595,67	0,70
	SD			19,21	0,07	670,53	0,11
	50μM	Exp1	98,20	58,60	0,79	1435,00	0,74
		Exp2	99,30	47,60	0,49	1199,00	0,32
		Exp3	95,00	55,00	0,85	989,00	0,72
	Mean			53,73	0,71	1207,67	0,60
	SD			5,61	0,20	223,13	0,24
	100μM	Exp1	97,80	57,60	0,78	1501,00	0,78
		Exp2	98,30	77,30	0,79	1285,00	0,34
		Exp3	99,10	54,40	0,84	978,00	0,72
	Mean			63,10	0,80	1254,67	0,61
	SD			12,40	0,03	262,82	0,24

Fig3A

Cell types	Groups	Experiments	Con. of FITC-Dextran (mg/ml)	Relative value of NC
HBEC	NC	Exp1	0,007	1,00
		Exp2	0,016	1,00
		Exp3	0,039	1,00
	Mean		0,021	1,00
	SD		0,017	0,00
	10μg/L	Exp1	0,007	1,04
		Exp2	0,016	1,02
		Exp3	0,038	0,97
	Mean		0,021	1,01
	SD		0,016	0,03
	50μg/L	Exp1	0,007	0,96
		Exp2	0,016	1,05
		Exp3	0,033	0,85
	Mean		0,019	0,95
	SD		0,013	0,10
	50μM	Exp1	0,010	1,41
		Exp2	0,020	1,25
		Exp3	0,043	1,10
	Mean		0,024	1,26
	SD		0,017	0,16
	100μM	Exp1	0,010	1,38
		Exp2	0,026	1,67
		Exp3	0,054	1,37
	Mean		0,030	1,47
	SD		0,022	0,17
HAEC	NC	Exp1	0,040	1,00
		Exp2	0,022	1,00
		Exp3	0,039	1,00
	Mean		0,034	1,00
	SD		0,010	0,00
	10μg/L	Exp1	0,017	0,43
		Exp2	0,023	1,01
		Exp3	0,047	1,22
	Mean		0,029	0,89
	SD		0,016	0,41
	50μg/L	Exp1	0,021	0,52
		Exp2	0,025	1,13
		Exp3	0,036	0,94
	Mean		0,028	0,86
	SD		0,008	0,31
	50μM	Exp1	0,036	0,89
		Exp2	0,037	1,66
		Exp3	0,061	1,57
	Mean		0,044	1,37
	SD		0,014	0,42
	100μM	Exp1	0,038	0,95
		Exp2	0,039	1,77
		Exp3	0,057	1,47
	Mean		0,045	1,40
	SD		0,011	0,42

Fig 3C-E

Cell types	Groups	Experiments	Raw AUC	Relative AUC
HBEC	NC	Exp1	27,94	1,00
		Exp2	27,46	1,00
		Exp3	29,13	1,00
		Exp4	77,93	1,00
		Exp5	51,22	1,00
		Exp6	52,92	1,00
	Mean		44,43	1,00
	SD		20,17	0,00
	10μg/L	Exp1	27,47	0,98
		Exp2	27,12	0,99
		Exp3	28,52	0,98
		Exp4	67,3	0,86
		Exp5	51,36	1,00
		Exp6	51,14	0,97
	Mean		42,15	0,96
	SD		16,89	0,05
	50μg/L	Exp1	26,90	0,96
		Exp2	26,93	0,98
		Exp3	28,30	0,97
		Exp4	69,06	0,89
		Exp5	50,62	0,99
		Exp6	49,12	0,93
	Mean		41,82	0,95
	SD		17,32	0,04
	50μM	Exp1	25,84	0,92
		Exp2	26,71	0,97
		Exp3	26,65	0,91
		Exp4	66,52	0,85
		Exp5	49,19	0,96
		Exp6	48,68	0,92
	Mean		40,60	0,92
	SD		16,83	0,04
	100μM	Exp1	25,78	0,92
		Exp2	25,95	0,95
		Exp3	27,25	0,94
	Mean		30,85	0,78
	SD		11,59	0,36
HAEC	NC	Exp1	28,60	1,00
		Exp2	29,55	1,00
		Exp3	27,76	1,00
		Exp4	59,54	1,00
		Exp5	64,77	1,00
		Exp6	62,72	1,00
	Mean		45,49	1,00
	SD		18,55	0,00
	10μg/L	Exp1	27,12	0,95
		Exp2	28,48	0,96
		Exp3	28,38	1,02
		Exp4	57,33	0,96
		Exp5	64,39	0,99
		Exp6	60,98	0,97
	Mean		44,45	0,98
	SD		18,17	0,03
	50μg/L	Exp1	27,69	0,97
		Exp2	28,59	0,97
		Exp3	27,81	1,00
		Exp4	53,48	0,90
		Exp5	65,7	1,01
		Exp6	60,61	0,97
	Mean		43,98	0,97
	SD		17,90	0,04
	50μM	Exp1	27,54	0,96
		Exp2	27,59	0,93
		Exp3	25,44	0,92
		Exp4	49,77	0,84
		Exp5	60,23	0,93
		Exp6	58,24	0,93
	Mean		41,47	0,92
	SD		16,41	0,04
	100μM	Exp1	26,82	0,94
		Exp2	27,08	0,92
		Exp3	26,19	0,94
	Mean		26,70	0,93
	SD		0,46	0,01

FigS1

HBEC

Confluence normalized to 0h(%)

	NC					10ug/L					50ug/L					50uM (13488ug/l)					200000ug/L			
Elapsed	Exp1	Exp2	Exp3	MEAN	SD	Exp1	Exp2	Exp3	MEAN	SD	Exp1	Exp2	Exp3	MEAN	SD	Exp1	Exp2	Exp3	MEAN	SD	Exp1	Exp2	MEAN	SD
0h	100,00	100,00	100,00	100,00	0,00	100,00	100,00	100,00	100,00	0,00	100,00	100,00	100,00	100,00	0,00	100,00	100,00	100,00	100,00	0,00	100,00	100,00	66,67	0,00
4h	120,18	117,99	115,99	118,05	2,10	121,75	119,18	115,69	118,87	3,04	120,53	114,88	108,34	114,58	6,10	117,02	116,14	110,00	114,39	3,82	104,15	102,59	70,19	1,10
8h	130,05	125,22	120,50	125,26	4,77	122,50	129,05	120,25	123,93	4,57	121,52	119,05	126,46	122,35	3,77	124,72	119,95	112,74	119,14	6,03	108,39	105,84	73,42	1,81
12h	133,33	129,74	126,95	130,01	3,20	126,54	132,33	125,95	128,27	3,52	125,40	124,82	129,69	126,64	2,66	132,22	125,30	114,66	124,06	8,85	112,19	108,95	76,66	2,29
16h	138,18	134,65	131,52	134,78	3,33	132,19	137,18	130,87	133,41	3,33	131,92	129,96	134,58	132,15	2,32	131,56	128,85	120,30	126,90	5,88	111,50	111,06	76,15	0,31
20h	139,87	138,36	136,75	138,33	1,56	135,62	138,87	136,55	137,01	1,67	134,81	135,73	134,71	135,08	0,56	129,56	131,77	125,60	128,98	3,13	115,31	112,82	77,08	1,76
24h	148,01	144,13	141,85	144,67	3,12	139,06	147,01	139,92	142,00	4,36	139,06	138,96	142,75	140,26	2,16	136,20	138,50	130,50	135,07	4,12	120,39	115,80	80,10	3,24
28h	153,27	149,26	145,54	149,36	3,86	145,49	152,27	144,86	147,54	4,11	148,30	143,92	147,55	146,59	2,34	138,29	140,64	140,56	139,83	1,33	123,86	117,05	80,75	4,81
32h	153,25	149,41	143,08	148,58	5,14	144,05	152,25	147,18	147,83	4,14	143,50	149,20	146,92	146,54	2,87	140,43	139,41	142,90	140,91	1,79	120,50	116,68	79,66	2,70
36h	154,44	151,16	144,98	150,19	4,80	145,83	153,44	149,46	149,58	3,80	146,90	148,90	147,46	147,75	1,03	140,92	139,72	143,80	141,48	2,10	121,26	117,51	80,29	2,65
40h	154,32	150,55	145,08	149,98	4,64	147,48	152,32	149,36	149,72	2,44	144,53	148,00	147,91	146,81	1,98	141,54	138,31	143,90	141,25	2,81	119,53	117,21	79,85	1,64
44h	158,11	153,25	148,09	153,15	5,01	149,73	156,11	148,20	151,35	4,20	149,73	145,30	147,23	147,42	2,22	141,20	140,73	139,50	140,47	0,88	122,24	115,82	79,64	4,54

HBEC

Dead cells normalized to 0h, to NC(%)

	NC					10ug/L					50ug/L					50uM (13488ug/l)					200000ug/L				
Elapsed	Exp1	Exp2	Exp3	MEAN	SD	Exp1	Exp2	Exp3	MEAN	SD	Exp1	Exp2	Exp3	MEAN	SD	Exp1	Exp2	Exp3	MEAN	SD	Exp1	Exp2	Exp3	MEAN	SD
0h	100,00	100,00	100,00	100,00	0,00	102,88	110,45	129,63	114,32	13,79	75,96	85,51	91,36	84,28	7,78	91,72	84,85	99,88	92,15	7,52	123,66	111,74	123,49	119,63	6,83
4h	109,72	110,71	116,30	112,25	3,55	126,02	118,71	143,80	129,51	12,91	89,10	90,26	111,31	96,89	12,50	135,44	121,39	134,25	130,36	7,79	169,83	131,99	136,40	146,07	20,69
8h	129,17	141,67	130,44	133,76	6,88	150,89	171,02	152,70	158,20	11,14	194,27	122,33	130,21	148,94	39,46	169,83	155,56	138,55	154,65	15,66	212,11	193,27	161,10	188,83	25,79
12h	137,50	148,81	160,87	149,06	11,69	144,02	195,88	184,78	174,89	27,31	251,24	180,52	142,81	191,52	55,04	256,54	214,49	201,91	224,31	28,61	282,34	241,59	200,84	241,59	40,75
16h	180,56	178,57	189,13	182,75	5,61	241,42	242,28	208,73	230,81	19,12	286,30	218,53	168,02	224,28	59,35	247,23	247,49	186,88	227,20	34,92	287,35	278,13	225,54	263,67	33,35
20h	245,83	235,72	201,09	227,55	23,47	330,58	264,84	226,31	273,91	52,72	353,49	275,53	221,57	283,53	66,32	279,47	242,77	220,17	247,47	29,93	476,53	414,83	364,09	418,48	56,31
24h	293,06	314,29	275,00	294,11	19,66	410,13	344,41	288,93	347,83	60,68	474,72	359,85	353,89	396,15	68,11	303,17	400,69	381,22	361,69	51,61	667,87	533,86	512,30	571,34	84,28
28h	501,39	442,26	361,96	435,20	69,98	526,73	385,39	373,52	428,54	85,24	547,76	476,24	413,74	479,25	67,06	560,38	557,43	484,37	534,06	43,06	846,30	727,14	732,47	768,63	67,31
32h	608,33	623,81	544,57	592,24	42,00	711,90	551,66	527,32	596,96	100,29	715,74	616,38	536,60	622,91	89,75	719,46	705,92	663,73	696,37	29,07	1056,98	914,52	964,45	978,65	72,28
36h	805,56	760,72	613,05	726,44	100,73	805,18	765,43	691,01	753,87	57,96	904,17	770,78	630,06	768,34	137,07	933,72	826,13	704,54	821,47	114,66	1288,44	1084,22	1070,78	1147,81	121,97
40h	1008,34	904,77	772,83	895,31	118,04	1016,42	907,36	748,13	890,64	134,92	1118,89	862,23	783,38	921,50	175,43	1041,21	944,57	889,27	958,35	76,90	1647,45	1371,78	1288,80	1436,01	187,75
44h	1222,23	1058,93	938,05	1073,07	142,61	1227,66	1050,47	1016,19	1098,10	113,50	1316,08	1140,13	971,35	1142,52	172,38	1272,67	1222,11	1068,63	1187,80	106,26	1906,14	1592,16	1516,49	1671,60	206,61

HAEC

Confluence normalized to 0h(%)

Elapsed	NC					10ug/L					50ug/L					50uM (13488ug/l)					200000ug/L				
	Exp1	Exp2	Exp3	MEAN	SD	Exp1	Exp2	Exp3	MEAN	SD	Exp1	Exp2	Exp3	MEAN	SD	Exp1	Exp2	Exp3	MEAN	SD	Exp1	Exp2	Exp3	MEAN	SD
0h	100,00	100,00	100,00	100,00	0,00	100,00	100,00	100,00	100,00	0,00	100,00	100,00	100,00	100,00	0,00	100,00	100,00	100,00	100,00	0,00	100,00	100,00	100,00	100,00	0,00
4h	122,84	111,73	117,61	117,39	5,56	124,35	113,94	111,10	116,46	6,97	119,69	115,38	114,47	116,51	2,79	126,45	116,49	115,43	119,46	6,08	120,51	105,28	107,41	111,06	8,25
8h	132,35	120,87	134,09	129,11	7,18	130,87	124,68	121,77	125,77	4,64	130,23	121,82	121,17	124,41	5,05	132,52	118,76	120,91	124,06	7,40	123,62	109,23	109,96	114,27	8,10
12h	136,94	128,97	143,16	136,36	7,11	137,06	129,17	128,43	131,55	4,79	132,41	126,22	132,64	130,42	3,64	136,93	127,62	127,95	130,83	5,28	123,22	112,49	115,27	116,99	5,57
16h	138,29	137,97	149,45	141,90	6,54	141,91	135,18	130,48	135,86	5,74	139,63	133,31	129,57	134,17	5,08	137,05	126,87	134,55	132,82	5,30	123,90	112,67	114,94	117,17	5,94
20h	140,59	138,55	150,80	143,31	6,57	143,51	136,51	131,34	137,12	6,11	144,41	136,08	134,22	138,24	5,43	140,58	128,54	135,57	134,90	6,05	125,46	116,43	118,32	120,07	4,76
24h	142,48	142,20	155,12	146,60	7,38	150,16	141,52	140,72	144,13	5,24	148,36	142,58	136,43	142,46	5,97	146,09	141,12	136,14	141,12	4,98	127,85	117,95	116,56	120,79	6,16
28h	143,62	147,28	154,92	148,61	5,77	151,81	141,81	146,08	146,57	5,02	150,52	139,98	141,18	143,89	5,77	144,99	143,08	137,59	141,89	3,84	128,33	116,09	118,40	120,94	6,50
32h	151,80	149,15	157,76	152,90	4,41	158,20	145,66	149,43	151,10	6,44	154,84	142,36	149,53	148,91	6,26	148,03	144,44	141,12	144,53	3,46	129,65	117,26	118,86	121,93	6,74
36h	145,08	151,44	156,84	151,12	5,88	161,29	151,49	153,82	155,53	5,12	160,55	150,62	146,19	152,45	7,36	148,99	150,03	142,34	147,12	4,17	130,39	117,89	118,86	122,38	6,96
40h	151,12	162,98	161,21	158,44	6,40	160,22	153,76	153,21	155,73	3,90	162,39	155,12	152,81	156,78	5,00	158,50	158,28	146,81	154,53	6,69	130,63	118,95	128,70	126,09	6,26
44h	158,45	162,92	163,96	161,78	2,92	161,37	158,08	158,99	159,48	1,70	160,00	154,35	157,05	157,13	2,82	165,39	156,73	153,46	158,53	6,16	135,23	117,96	118,87	124,02	9,72

HAEC

Dead cells normalized to 0h, to NC(%)

Elapsed	NC					10ug/L					50ug/L					50uM (13488ug/l)					200000ug/L				
	Exp1	Exp2	Exp3	Mean	SD	Exp1	Exp2	Exp3	Mean	SD	Exp1	Exp2	Exp3	Mean	SD	Exp1	Exp2	Exp3	Mean	SD	Exp1	Exp2	Exp3	Mean	SD
0h	100,00	100,00	100,00	100,00	0,00	105,30	103,60	88,11	99,00	9,47	88,25	109,72	96,55	98,17	10,82	109,78	103,04	89,62	100,81	10,26	76,56	106,09	106,33	96,33	17,12
4h	97,34	111,76	90,80	99,97	10,73	106,85	97,54	79,18	94,52	14,08	85,29	108,08	90,18	94,52	12,00	107,07	109,60	79,20	98,62	16,87	89,92	99,02	101,03	96,66	5,92
8h	73,94	80,39	68,71	74,35	5,85	88,26	86,98	72,03	82,43	9,02	81,74	103,17	77,88	87,59	13,62	103,00	82,43	70,34	85,26	16,51	93,57	88,80	97,97	93,45	4,59
12h	74,47	72,79	70,55	72,60	1,96	78,46	76,66	62,51	72,54	8,73	73,45	78,60	72,41	74,82	3,32	96,22	88,05	72,95	85,74	11,81	104,12	88,02	103,80	98,65	9,21
16h	81,91	64,71	72,39	73,00	8,62	75,88	75,13	60,13	70,38	8,89	76,41	79,42	90,18	82,00	7,24	81,99	86,18	77,11	81,76	4,54	101,86	95,09	97,20	98,05	3,46
20h	77,66	64,71	75,46	72,61	6,93	79,49	74,97	55,96	70,14	12,49	78,18	73,69	94,28	82,05	10,82	90,80	88,05	85,97	88,28	2,42	115,20	121,65	123,16	120,00	4,23
24h	91,49	83,82	94,48	89,93	5,50	94,97	85,38	61,91	80,76	17,01	92,40	85,15	101,11	92,89	7,99	98,26	93,67	108,90	100,28	7,81	129,78	133,91	152,96	138,88	12,36
28h	101,06	87,75	83,84	90,88	9,03	114,59	104,25	75,61	98,15	20,19	101,88	97,43	112,49	103,93	7,74	136,21	111,47	114,63	120,77	13,46	190,54	178,23	215,28	194,68	18,87
32h	120,74	100,98	117,18	112,97	10,53	130,07	122,23	89,89	114,06	21,30	129,72	148,20	131,62	136,51	10,17	142,10	136,58	132,71	137,13	4,72	289,21	255,25	283,26	275,90	18,13
36h	160,64	127,94	127,61	138,73	18,97	174,46	149,22	109,54	144,41	32,73	165,85	192,41	163,05	173,77	16,21	193,33	178,73	151,47	174,51	21,25	374,27	316,86	345,57	345,57	28,71
40h	214,89	172,06	163,19	183,38	27,65	225,56	197,81	153,00	192,12	36,61	221,52	252,18	195,38	223,03	28,43	230,53	241,12	203,05	224,90	19,65	444,02	429,08	414,15	429,08	14,94
44h	267,55	202,94	184,05	218,18	43,79	258,60	238,32	198,84	231,92	30,39	263,58	307,86	220,43	263,96	43,72	290,91	295,07	232,60	272,86	34,93	575,01	568,18	525,80	556,33	26,66

FigS4

		CAV1		CLDN5		EDN1		ICAM1		IL6	
		Abosulte mRNA value	Relative value of NC	Abosulte mRNA value	Relative value of NC	Abosulte mRNA value	Relative value of NC	Abosulte mRNA value	Relative value of NC	Abosulte mRNA value	Relative value of NC
	Exp1	6072	1,00	1651	1,00	4174	1,00	643	1,00	140	1,00
NC	Exp2	7032	1,00	2196	1,00	4299	1,00	644	1,00	81	1,00
	Exp3	6945	1,00	2717	1,00	2837	1,00	624	1,00	100	1,00
	**Mean**	6683	1,00	2188	1,00	3770	1,00	637	1,00	107	1,00
	**SD**	531	0,00	533	0,00	811	0,00	11	0,00	30	0,00
	Exp1	6117	1,01	3800	2,30	5792	1,39	598	0,93	112	0,80
50ug/L MPH	Exp2	5607	0,80	4170	1,90	4899	1,14	858	1,33	76	0,94
	Exp3	5793	0,83	5036	1,85	2487	0,88	487	0,78	92	0,91
	**Mean**	5839	0,88	4335	2,02	4393	1,13	647	1,01	93	0,88
	**SD**	258	0,11	634	0,25	1710	0,26	190	0,29	18	0,07
	Exp1	5835	0,96	3505	2,12	5179	1,24	717	1,12	109	0,78
50uM MPH	Exp2	5837	0,83	4197	1,91	5040	1,17	854	1,33	60	0,74
	Exp3	6009	0,87	5488	2,02	2358	0,83	457	0,73	80	0,80
	**Mean**	5894	0,89	4397	2,02	4192	1,08	676	1,06	83	0,77
	**SD**	100	0,07	1006	0,11	1591	0,22	201	0,30	25	0,03

		IL8		JAM_A		MCP		NOX1		OCLN	
		Abosulte mRNA value	Relative value of NC	Abosulte mRNA value	Relative value of NC	Abosulte mRNA value	Relative value of NC	Abosulte mRNA value	Relative value of NC	Abosulte mRNA value	Relative value of NC
	Exp1	800	1,00	770	1,00	3616	1,00	31	1,00	96	1,00
NC	Exp2	315	1,00	1140	1,00	4238	1,00	38	1,00	100	1,00
	Exp3	657	1,00	990	1,00	3857	1,00	39	1,00	139	1,00
	**Mean**	591	1,00	967	1,00	3904	1,00	36	1,00	112	1,00
	**SD**	249	0,00	186	0,00	314	0,00	4	0,00	24	0,00
	Exp1	378	0,47	1139	1,48	3998	1,11	27	0,87	106	1,10
50ug/L MPH	Exp2	307	0,98	1085	0,95	3673	0,87	43	1,15	172	1,72
	Exp3	429	0,65	1493	1,51	2855	0,74	54	1,41	180	1,30
	**Mean**	372	0,70	1239	1,31	3509	0,90	42	1,15	153	1,37
	**SD**	61	0,26	222	0,31	589	0,19	14	0,27	41	0,32
	Exp1	380	0,47	1116	1,45	3883	1,07	34	1,09	109	1,14
50uM MPH	Exp2	228	0,72	1090	0,96	4089	0,96	53	1,40	162	1,62
	Exp3	429	0,65	1525	1,54	2953	0,77	56	1,45	169	1,22
	**Mean**	345	0,62	1244	1,31	3642	0,93	47	1,31	147	1,33
	**SD**	105	0,13	244	0,31	605	0,16	12	0,19	33	0,26

		PAI_1		PECAM1		RAC1		SELE		SELP	
		Abosulte mRNA value	Relative value of NC	Abosulte mRNA value	Relative value of NC	Abosulte mRNA value	Relative value of NC	Abosulte mRNA value	Relative value of NC	Abosulte mRNA value	Relative value of NC
	Exp1	30785	1,00	11003	1,00	3251	1,00	68	1,00	470	1,00
NC	Exp2	28848	1,00	13484	1,00	3181	1,00	75	1,00	437	1,00
	Exp3	21052	1,00	13477	1,00	2867	1,00	43	1,00	274	1,00
	**Mean**	26895	1,00	12655	1,00	3100	1,00	62	1,00	394	1,00
	**SD**	5152	0,00	1431	0,00	205	0,00	17	0,00	105	0,00
	Exp1	25138	0,82	15605	1,42	3045	0,94	32	0,48	674	1,44
50ug/L MPH	Exp2	23277	0,81	18423	1,37	2781	0,87	94	1,25	746	1,71
	Exp3	10461	0,50	19822	1,47	2967	1,04		0,00	79	0,29
	**Mean**	19625	0,71	17950	1,42	2931	0,95	63	0,58	500	1,14
	**SD**	7991	0,18	2148	0,05	136	0,08	44	0,63	366	0,75
	Exp1	24262	0,79	14538	1,32	3034	0,93	53	0,78	673	1,43
50uM MPH	Exp2	23137	0,80	19572	1,45	2894	0,91	104	1,38	874	2,00
	Exp3	9799	0,47	20769	1,54	2815	0,98	41	0,97	105	0,38
	**Mean**	19066	0,69	18293	1,44	2914	0,94	66	1,04	551	1,27
	**SD**	8045	0,19	3307	0,11	111	0,04	33	0,31	399	0,82

		TGFb		VCAM1		ZO1		eNOS		tPA		vWF	
		Abosulte mRNA value	Relative value of NC	Abosulte mRNA value	Relative value of NC	Abosulte mRNA value	Relative value of NC	Abosulte mRNA value	Relative value of NC	Abosulte mRNA value	Relative value of NC	Abosulte mRNA value	Relative value of NC
	Exp1	6176	1,00	766	1,00	923	1,00	695	1,00	4078	1,00	9746	1,00
NC	Exp2	6715	1,00	629	1,00	1179	1,00	880	1,00	3689	1,00	11988	1,00
	Exp3	7024	1,00	532	1,00	932	1,00	825	1,00	4674	1,00	7563	1,00
	**Mean**	6638	1,00	642	1,00	1012	1,00	800	1,00	4147	1,00	9766	1,00
	**SD**	429	0,00	117	0,00	145	0,00	95	0,00	496	0,00	2213	0,00
	Exp1	5456	0,88	408	0,53	1134	1,23	909	1,31	3747	0,92	15015	1,54
50ug/L MPH	Exp2	5757	0,86	465	0,74	1004	0,85	714	0,81	3043	0,83	15494	1,29
	Exp3	5807	0,83	75	0,14	1166	1,25	636	0,77	4103	0,88	16332	2,16
	**Mean**	5673	0,86	316	0,47	1102	1,11	753	0,96	3631	0,87	15613	1,66
	**SD**	190	0,03	211	0,30	86	0,22	141	0,30	539	0,05	667	0,45
	Exp1	4988	0,81	488	0,64	1047	1,13	799	1,15	3946	0,97	13932	1,43
50uM MPH	Exp2	6028	0,90	486	0,77	965	0,82	795	0,90	3024	0,82	16906	1,41
	Exp3	5818	0,83	60	0,11	1226	1,32	662	0,80	4045	0,87	16752	2,22
	**Mean**	5611	0,84	344	0,51	1079	1,09	752	0,95	3672	0,88	15863	1,68
	**SD**	550	0,05	247	0,35	134	0,25	78	0,18	563	0,08	1674	0,46

FigS5

Cell types	Group	Experiments	tPA		vWF	
			Abosulte mRNA value	Conc. (ng/ml)	Abosulte mRNA value	Conc. (ng/ml)
HBEC	NC	Exp1	4176	9,55	9459	66,61
		Exp2	6022	9,40	14983	70,99
		Exp3	6510	9,64	8358	77,75
	10ug/L MPH	Exp1	3114	32,28	18225	169,44
		Exp2	3322	23,52	24454	286,93
		Exp3	4577	27,95	8595	261,45
	50ug/L MPH	Exp1	3100	30,74	18035	175,62
		Exp2	3652	22,80	22380	283,31
		Exp3	4863	28,07	9662	243,73
	50uM MPH	Exp1	3030	31,05	18103	171,97
		Exp2	3308	22,98	24244	266,29
		Exp3	4482	27,13	10578	227,12
HAEC	NC	Exp1	4078	9,73	9746	49,60
		Exp2	3689	2,56	11988	64,31
		Exp3	4674	4,27	7563	54,70
	50ug/L MPH	Exp1	3747	33,38	15015	174,96
		Exp2	3043	8,42	15494	219,96
		Exp3	4103	9,35	16332	216,29
	50uM MPH	Exp1	3946	34,05	13932	186,37
		Exp2	3024	9,58	16906	227,11
		Exp3	4045	8,81	16752	227,44

FigS6								
ID	Group	Sex	Age	tPA (ng/ml)	vWF (ng/ml)	CRP (pg/ml)	ICAM1 (pg/ml)	VCAM1 (pg/ml)
336	MPH ongoing	Male	18	1,78	11131	171867	551962	602501
24	MPH ongoing	Male	13	1,54	13680	1170147	1855564	1786821
52	MPH ongoing	Female	15	1,23	26954	684516	867326	1031437
210	MPH ongoing	Male	12	0,76	12049	998977	519165	611392
283	MPH ongoing	Male	15	0,94	7238	2219517	851162	937667
295	MPH ongoing	Male	14	5,26	11038	189287	563373	507921
348	MPH ongoing	Female	18	2,62	17808	5075991	460321	418378
23	MPH ongoing	Female	11	3,08	3825	266694	1544563	1360519
31	MPH ongoing	Male	14	1,37	13935	1660080	2265905	3558995
34	MPH ongoing	Male	14	1,11	13758	1709368	928274	1463140
130	MPH ongoing	Male	11	2,14	10889	347036	487117	588599
158	MPH ongoing	Male	16	1,34	10693	182205	367656	358066
54	MPH ongoing	Male	8	0,88	7625	72153	888454	985284
36	MPH ongoing	Female	8	0,73	5550	438094	1370974	1255856
51	MPH ongoing	Male	9	4,48	14814	51478	399021	520559
48	MPH ongoing	Male	13	2,02	8740	3092387	914517	965338
53	MPH ongoing	Male	8	1,24	7427	42196	940878	986720
202	Non-MPH ongoing	Male	14	1,75	6876	247973	656246	590941
281	Non-MPH ongoing	Male	13	0,59	12862	757771	610521	563951
20	Non-MPH ongoing	Male	11	1,34	9803	847221	1813180	1463566
30	Non-MPH ongoing	Female	14	1,80	11212	553976	1516056	2039694
32	Non-MPH ongoing	Male	8	0,73	8148	3360379	2536883	2425647
39	Non-MPH ongoing	Male	8	3,50	9806	251817	1716800	1550236
55	Non-MPH ongoing	Male	14	0,91	7832	1352481	647550	671565
57	Non-MPH ongoing	Female	13	1,79	8977	745053	536634	539637
131	Non-MPH ongoing	Male	12	1,99	10248	317664	638453	595018
146	Non-MPH ongoing	Male	16	0,29	11131	260354	453737	637960
160	Non-MPH ongoing	Male	13	1,03	8292	619103	736469	973565
41	Non-MPH ongoing	Male	13	2,66	2002	352421	888628	1322788
45	Non-MPH ongoing	Male	13	2,71	3159	3628572	1694366	950027
179	Non-MPH ongoing	Male	14	1,97	5936	155958	748487	699989
121	Drug-naïve	Female	11	1,26	3561	486004	606203	760512
311	Drug-naïve	Male	15	2,55	11667	2752110	609574	460643
343	Drug-naïve	Female	16	1,21	3039	1032100	471534	426335
317	Drug-naïve	Male	11	1,34	8163	190462	610040	705178
350	Drug-naïve	Male	9	0,66	7568	115047	463143	731576
328	Drug-naïve	Male	13	1,48	8932	2955641	784032	674522
354	Drug-naïve	Male	10	0,95	8614	169386	571022	638909
17	Drug-naïve	Female	11	5,19	3719	694771	2018253	1714731
50	Drug-naïve	Male	15	1,63	7702	274770	653798	692803
80	Drug-naïve	Female	18	3,86	4688	133864	436917	360486
159	Drug-naïve	Male	10	1,18	10189	1483812	518273	463021
221	Drug-naïve	Female	15	1,45	8724	123347	428259	555336
306	Drug-naïve	Male	12	1,62	10509	436195	477150	524943
310	Drug-naïve	Male	10	0,80	12078	363664	557853	500390
137	Drug-naïve	Female	17	2,19	1819	58054	386825	469044
138	Drug-naïve	Female	14	1,41	6090	270262	293910	303166
145	Drug-naïve	Male	8	1,42	7469	703760	709945	688135

FigS8B

Cell types	Groups	Experiments	CLDN5	bactin	CLDN5 (Norm by bactin)	CLDN5 (Relative value of NC)
HBEC	NC	Exp1	22240	19829	1,12	1,00
		Exp2	27936	22507	1,24	1,00
		Exp3	28122	19398	1,45	1,00
	Mean				1,27	1,00
	SD				0,17	0,00
	10μg/L	Exp1	30120	23312	1,29	1,15
		Exp2	26982	21816	1,24	1,00
		Exp3	23246	27243	0,85	0,59
	Mean				1,13	0,91
	SD				0,24	0,29
	50μg/L	Exp1	24444	26714	0,92	0,82
		Exp2	17506	23568	0,74	0,60
		Exp3	29154	32997	0,88	0,61
	Mean				0,85	0,67
	SD				0,09	0,12
	50μM	Exp1	22046	23476	0,94	0,84
		Exp2	14084	24352	0,58	0,47
		Exp3	17590	32848	0,54	0,37
	Mean				0,68	0,56
	SD				0,22	0,25
	100μM	Exp1	8346	35979	0,23	0,21
		Exp2	16594	32095	0,52	0,42
		Exp3	20560	27581	0,75	0,51
	Mean				0,50	0,38
	SD				0,26	0,16
HAEC	NC	Exp1	31080	26355	1,18	1,00
		Exp2	30601	21009	1,46	1,00
		Exp3	22315	25492	0,88	1,00
	Mean				1,17	1,00
	SD				0,29	0,00
	10μg/L	Exp1	30993	33536	0,92	0,78
		Exp2	27106	28674	0,95	0,65
		Exp3	27392	25636	1,07	1,22
	Mean				0,98	0,88
	SD				0,08	0,30
	50μg/L	Exp1	26927	34381	0,78	0,66
		Exp2	20461	29086	0,70	0,48
		Exp3	20554	21235	0,97	1,11
	Mean				0,82	0,75
	SD				0,14	0,32
	50μM	Exp1	18428	32208	0,57	0,49
		Exp2	22886	20536	0,86	0,59
		Exp3	17890	26448	0,68	0,77
	Mean				0,70	0,62
	SD				0,15	0,15
	100μM	Exp1	14646	31095	0,47	0,40
		Exp2	7254	25579	0,28	0,19
		Exp3	14229	33431	0,43	0,49
	Mean				0,39	0,36
	SD				0,10	0,15

FigS8D

Cell types	Groups	Experiments	Cell number	DAPI	Claudin5		
				total intensity	total intensity	total intensity / cell number	Relative value of NC
HBEC	NC	Exp1	91	38557530	25625160	281595	1,00
		Exp2	92	41254830	28742310	312416	1,00
		Exp3	97	43361910	32973750	339936	1,00
		Exp4	94	42660450	34171290	363524	1,00
	Mean		94	41458680	30378128	324368	1,00
	SD		3	2123265	3932427	35346	0,00
	10ug/L MPH	Exp1	101	47342430	31912830	315969	1,12
		Exp2	96	47972790	30275640	315371	1,01
		Exp3	89	46285560	29701530	333725	0,98
		Exp4	94	45966960	36409410	387334	1,07
	Mean		95	46891935	32074853	338100	1,04
	SD		5	929957	3037788	33909	0,06
	50ug/L MPH	Exp1	90	44317440	19519110	216879	0,77
		Exp2	91	43370100	24573330	270037	0,86
		Exp3	77	36549900	24697800	320751	0,94
		Exp4	88	43673130	30617910	347931	0,96
	Mean		87	41977643	24852038	288899	0,88
	SD		6	3639993	4538240	57856	0,09
	50uM MPH	Exp1	96	37609470	19442160	202523	0,72
		Exp2	76	37449810	16189470	213019	0,68
		Exp3	76	36227790	16752060	220422	0,65
		Exp4	77	35849700	22601610	293527	0,81
	Mean		81	36784193	18746325	232373	0,71
	SD		10	876924	2936098	41426	0,07
	100uM MPH	Exp1	83	37137690	12917340	155631	0,55
		Exp2	86	40358970	15137460	176017	0,56
		Exp3	94	36205830	17421570	185336	0,55
		Exp4	91	37965510	18847800	207119	0,57
	Mean		89	37917000	16081043	181026	0,56
	SD		5	1779606	2604568	21365	0,01
HAEC	NC	Exp1	152	12214265	11208963	73743	1,00
		Exp2	136	10928430	8727333	64172	1,00
		Exp3	143	10750408	6469590	45242	1,00
	Mean		109	11297701	8801962	61052	1,00
	SD		70	798743	2370568	14504	0,00
	10ug/L MPH	Exp1	136	10798306	10125001	74449	1,01
		Exp2	172	14689605	12547083	72948	1,14
		Exp3	139	8799812	9748352	70132	1,55
	Mean		129	11429241	10806812	72510	1,23
	SD		43	2995159	1518840	2191	0,28
	50ug/L MPH	Exp1	164	11615259	13986016	85281	1,16
		Exp2	148	10735186	6879806	46485	0,72
		Exp3	144	11231090	2841734	19734	0,44
	Mean		125	11193845	7902519	50500	0,77
	SD		55	441217	5642093	32957	0,36
	50uM MPH	Exp1	135	13693365	5487883	40651	0,55
		Exp2	147	12563358	2215421	15071	0,23
		Exp3	144	10463902	2020031	14028	0,31
	Mean		120	11513630	2117726	14549	0,27
	SD		44	1484540	138162	737	0,05
	100uM MPH	Exp1	145	11473699	4645847	32040	0,43
		Exp2	133	11885652	4088912	30744	0,48
		Exp3	139	10257753	2499649	17983	0,40
	Mean		115	11071703	3294281	24363	0,44
	SD		48	1151098	1123779	9023	0,06

FigS9B

Cell types	Groups	Experiments	PECAM1	bactin	PECAM1 (Norm by bactin)	PECAM1 (Relative value of NC)
HBEC	NC	Exp1	25346	25381	1,00	1,00
		Exp2	22961	24851	0,92	1,00
		Exp3	23006	29810	0,77	1,00
		Exp4	27017	26939	1,00	1,00
		Exp5	24594	26426	0,93	1,00
	Mean				0,93	1,00
	SD				0,09	0,00
	10μg/L	Exp1	22746	29730	0,77	0,77
		Exp2	22955	28577	0,80	0,87
		Exp3	19135	30106	0,64	0,82
		Exp4	23862	25792	0,93	0,92
		Exp5	25832	26539	0,97	1,05
	Mean				0,82	0,89
	SD				0,13	0,11
	50μg/L	Exp1	23134	30125	0,77	0,77
		Exp2	25695	34123	0,75	0,81
		Exp3	18457	30024	0,61	0,80
		Exp4	24979	32142	0,78	0,77
		Exp5	26770	26208	1,02	1,10
	Mean				0,79	0,85
	SD				0,15	0,14
	50μM	Exp1	22649	26594	0,85	0,85
		Exp2	27097	30674	0,88	0,96
		Exp3	18640	20756	0,90	1,16
		Exp4	22973	31670	0,73	0,72
		Exp5	26698	27769	0,96	1,03
	Mean				0,86	0,95
	SD				0,09	0,17
	100μM	Exp1	22028	29279	0,75	0,75
		Exp2	27471	26695	1,03	1,11
		Exp3	18859	20326	0,93	1,20
		Exp4	23610	28317	0,83	0,83
		Exp5	24366	27463	0,89	0,95
	Mean				0,89	0,97
	SD				0,10	0,19
HAEC	NC	Exp1	20559	26649	0,77	1,00
		Exp2	27402	21399	1,28	1,00
		Exp3	24588	15910	1,55	1,00
	Mean				1,20	1,00
	SD				0,39	0,00
	10μg/L	Exp1	22736	30515	0,75	0,97
		Exp2	25264	26241	0,96	0,75
		Exp3	23761	28454	0,84	0,54
	Mean				0,85	0,75
	SD				0,11	0,21
	50μg/L	Exp1	22390	32806	0,68	0,88
		Exp2	27316	24537	1,11	0,87
		Exp3	20076	21388	0,94	0,61
	Mean				0,91	0,79
	SD				0,22	0,16
	50μM	Exp1	25720	30943	0,83	1,08
		Exp2	25026	26525	0,94	0,74
		Exp3	21196	22204	0,95	0,62
	Mean				0,91	0,81
	SD				0,07	0,24
	100μM	Exp1	26276	31957	0,82	1,07
		Exp2	23444	30535	0,77	0,60
		Exp3	29235	32101	0,91	0,59
	Mean				0,83	0,75
	SD				0,07	0,27

FigS9D

Cell types	Groups	Experiments	PECAM1	bactin	PECAM1 (Norm by bactin)	PECAM1 (Relative value of NC)
HBEC	NC	Exp1	22811	19829	1,15	1,00
		Exp2	26802	22507	1,19	1,00
		Exp3	23200	19398	1,20	1,00
		Exp4	23927	29049	0,82	1,00
	Mean		24185	22696	1,09	1,00
	SD		1805	4453	0,18	0,00
	10μg/L	Exp1	26094	23312	1,12	0,97
		Exp2	24764	21816	1,14	0,95
		Exp3	29851	27243	1,10	0,92
		Exp4	22967	23383	0,98	1,19
	Mean		25919	23938	1,08	1,01
	SD		2918	2318	0,07	0,12
	50μg/L	Exp1	27038	26714	1,01	0,88
		Exp2	22334	23568	0,95	0,80
		Exp3	28119	32997	0,85	0,71
		Exp4	22972	31170	0,74	0,89
	Mean		25116	28612	0,89	0,82
	SD		2890	4275	0,12	0,08
	50μM	Exp1	28200	23476	1,20	1,04
		Exp2	22863	24352	0,94	0,79
		Exp3	24971	32848	0,76	0,64
		Exp4	20601	27980	0,74	0,89
	Mean		24159	27164	0,91	0,84
	SD		3232	4262	0,21	0,17
	100μM	Exp1	23636	35979	0,66	0,57
		Exp2	26936	32095	0,84	0,70
		Exp3	23397	27581	0,85	0,71
		Exp4	27774	31321	0,89	1,08
	Mean		25436	31744	0,81	0,77
	SD		2244	3444	0,10	0,22
HAEC	NC	Exp1	19457	26355	0,74	1,00
		Exp2	26142	21009	1,24	1,00
		Exp3	26051	30814	0,85	1,00
	Mean		23883	26060	0,94	1,00
	SD		3834	4909	0,27	0,00
	10μg/L	Exp1	22422	33536	0,67	0,91
		Exp2	21791	28674	0,76	0,61
		Exp3	26857	30166	0,89	1,05
	Mean		23690	30792	0,77	0,86
	SD		2761	2490	0,11	0,23
	50μg/L	Exp1	22915	34381	0,67	0,90
		Exp2	27790	29086	0,96	0,77
		Exp3	22866	28729	0,80	0,94
	Mean		24524	30732	0,81	0,87
	SD		2829	3165	0,14	0,09
	50μM	Exp1	24720	32208	0,77	1,04
		Exp2	26385	20536	1,28	1,03
		Exp3	26075	28260	0,92	1,09
	Mean		25726	27002	0,99	1,05
	SD		885	5937	0,27	0,03
	100μM	Exp1	26696	31095	0,86	1,16
		Exp2	27786	25579	1,09	0,87
		Exp3	28183	27268	1,03	1,22
	Mean		27555	27981	0,99	1,09
	SD		770	2826	0,12	0,19

FigS9F

				DAPI	PECAM1		
Cell types	Group	Experiments	Cell number	total intensity	total intensity	total intensity / cell number	Ratio
HBEC	NC	Exp1	71	35382600	26489700	373094	1,00
		Exp2	67	31039110	21482910	320640	1,00
		Exp3	81	36892620	23058450	284672	1,00
		Exp4	73	33205500	23079510	316158	1,00
	Mean		73	34129958	23527643	323641	1,00
	SD		6	2556650	2111530	36648	0,00
	10ug/L MPH	Exp1	82	38311200	29425500	358848	0,96
		Exp2	92	44727210	34336800	373226	1,16
		Exp3	90	39923640	29216160	324624	1,14
		Exp4	83	41179590	29591730	356527	1,13
	Mean		87	41035410	30642548	353306	1,10
	SD		5	2726874	2467624	20498	0,09
	50ug/L MPH	Exp1	90	42426540	28030680	311452	0,83
		Exp2	79	42128190	28426320	359827	1,12
		Exp3	84	37661670	28649250	341063	1,20
		Exp4	82	41658300	30291120	369404	1,17
	Mean		84	40968675	28849343	345436	1,08
	SD		5	2227234	994638	25532	0,17
	50uM MPH	Exp1	85	37930680	25609950	301294	0,81
		Exp2	93	39240630	31575240	339519	1,06
		Exp3	82	38786400	30839310	376089	1,32
		Exp4	85	37460970	26544510	312288	0,99
	Mean		86	38354670	28642253	332297	1,04
	SD		5	806181	3001381	33324	0,21
	100uM MPH	Exp1	83	40731840	30190950	363746	0,97
		Exp2	95	39065580	29529270	310834	0,97
		Exp3	81	40541490	23492700	290033	1,02
		Exp4	79	40244760	27892530	353070	1,12
	Mean		85	40145918	27776363	329421	1,02
	SD		7	747592	3014751	34806	0,07
HAEC	NC	Exp1	193	7900518	1990023	10311	1,00
		Exp2	168	5996282	1751336	10425	1,00
		Exp3	170	6043975	1764213	10378	1,00
		Exp4	177	6626790	2087643	11795	1,00
	Mean		177	6641891	1898304	10727	1,00
	SD		11	886695	167174	713	0,00
	10ug/L MPH	Exp1	151	6243431	1269573	8408	0,82
		Exp2	151	5988977	1099787	7283	0,70
		Exp3	130	6026440	1330738	10236	0,99
		Exp4	139	5168464	1522236	10951	0,93
	Mean		143	5856828	1305584	9220	0,86
	SD		10	472419	174375	1677	0,13
	50ug/L MPH	Exp1	148	5297615	1256190	8488	0,82
		Exp2	156	5516741	1607478	10304	0,99
		Exp3	142	5085270	1353772	9534	0,92
		Exp4	152	4948223	1427115	9389	0,80
	Mean		150	5211962	1411139	9429	0,88
	SD		6	248888	148441	745	0,09
	50uM MPH	Exp1	164	6102762	1895333	11557	1,12
		Exp2	144	4866665	1448016	10056	0,96
		Exp3	127	4309896	1030322	8113	0,78
		Exp4	133	4221195	1207540	9079	0,77
	Mean		142	4875130	1395303	9701	0,91
	SD		16	866847	374733	1470	0,17
	100uM MPH	Exp1	147	4642856	1367998	9306	0,90
		Exp2	144	4666372	1478242	10266	0,98
		Exp3	127	3975225	1143662	9005	0,87
		Exp4	141	4976371	1530068	10852	0,92
	Mean		140	4565206	1379993	9857	0,92
	SD		9	421663	171437	853	0,05

FigS9H

Cell types	Group	Experiments	Cell number	total intensity	total intensity	total intensity / cell number	Ratio
HBEC	NC	Exp1	82	34529850	29512980	359914	1,00
		Exp2	85	36116820	25745760	302891	1,00
		Exp3	80	32558130	28339020	354238	1,00
	Mean		82	34401600	27865920	339014	1,00
	SD		3	1782808	1927655	31412	0,00
	10ug/L MPH	Exp1	88	42461640	31804020	361409	1,00
		Exp2	91	40045410	30715740	337536	1,11
		Exp3	85	39062610	29555640	347713	0,98
	Mean		88	40523220	30691800	348886	1,03
	SD		3	1749165	1124381	11980	0,07
	50ug/L MPH	Exp1	82	35189280	29334240	357735	0,99
		Exp2	89	39316050	30696210	344901	1,14
		Exp3	94	38182230	30692880	326520	0,92
	Mean		88	37562520	30241110	343052	1,02
	SD		6	2132038	785374	15689	0,11
	50uM MPH	Exp1	86	41655060	34469640	400810	1,11
		Exp2	92	34898490	29688840	322705	1,07
		Exp3	89	35340390	30785760	345907	0,98
	Mean		89	37297980	31648080	356474	1,05
	SD		3	3779805	2504338	40110	0,07
	100uM MPH	Exp1	82	35725500	29246850	356669	0,99
		Exp2	90	39588750	31370220	348558	1,15
		Exp3	97	40060620	29082510	299820	0,85
	Mean		90	38458290	29899860	335016	1,00
	SD		8	2378397	1276018	30749	0,15
HAEC	NC	Exp1	136	4852531	1039177	7641	1,00
		Exp2	144	5744298	1328794	9228	1,00
		Exp3	151	4794347	1512040	10014	1,00
		Exp4	146	5615769	1174788	8046	1,00
	Mean		144	5251736	1263700	8732	1,00
	SD		6	497898	203491	1088	0,00
	10ug/L MPH	Exp1	147	5497534	981880	6679	0,87
		Exp2	155	5041504	1250501	8068	0,87
		Exp3	151	5494731	1162766	7700	0,77
		Exp4	140	5316051	1034050	7386	0,92
	Mean		148	5337455	1107299	7458	0,86
	SD		6	214791	122037	589	0,06
	50ug/L MPH	Exp1	144	4869871	1134745	7880	1,03
		Exp2	146	4948665	1333016	9130	0,99
		Exp3	145	4419724	1352966	9331	0,93
		Exp4	158	4786203	1402800	8878	1,10
	Mean		150	4718197	1362927	9113	1,01
	SD		7	270949	35943	227	0,07
	50uM MPH	Exp1	146	5283945	1026101	7028	0,92
		Exp2	145	4783478	1151871	7944	0,86
		Exp3	134	4015106	1150508	8586	0,86
		Exp4	161	5917115	1305928	8111	1,01
	Mean		147	4999911	1158602	7917	0,91
	SD		11	803870	114560	652	0,07
	100uM MPH	Exp1	148	5020098	800752	5410	0,71
		Exp2	115	3873490	1199156	10427	1,13
		Exp3	144	5211038	1770983	12298	1,23
		Exp4	112	3870843	735748	6569	0,82
	Mean		136	4701542	1256964	9379	0,97
	SD		18	723441	487692	3562	0,25

FigS10

Cell types	Groups	Relative value of NC
HBEC	NC	1,00
		1,00
		1,00
	Mean	1,00
	SD	0,00
	10μg/L	0,91
		1,12
		0,92
	Mean	0,98
	SD	0,12
	50μg/L	1,15
		0,75
		0,87
	Mean	0,92
	SD	0,21
	100μM	1,30
		1,35
		1,28
	Mean	1,31
	SD	0,04
HAEC	NC	1,00
		1,00
		1,00
	Mean	1,00
	SD	0,00
	10μg/L	0,95
		1,05
		0,96
	Mean	0,99
	SD	0,06
	50μg/L	0,93
		0,98
		0,77
	Mean	0,89
	SD	0,11
	100μM	0,88
		0,95
		1,23
	Mean	1,02
	SD	0,19

FigS11

	Concentrations of vWF (ng/ml)				Concentrations of tPA (ng/ml)			
	Sample1	Sample2	Sample3	Sample4	Sample1	Sample2	Sample3	Sample4
Never thaw	11109,8	19204,3	21254,0	22601,5	7,69	7,69	6,23	7,16
Thaw one times	11764,6	19934,9	21263,5	24736,6	7,46	8,31	6,47	6,93
